# *TCGA Workflow*: Analyze cancer genomics and epigenomics data using Bioconductor packages

**DOI:** 10.12688/f1000research.8923.2

**Published:** 2016-12-28

**Authors:** Tiago C. Silva, Antonio Colaprico, Catharina Olsen, Fulvio D'Angelo, Gianluca Bontempi, Michele Ceccarelli, Houtan Noushmehr

**Affiliations:** 1 Department of Genetics, Ribeirao Preto Medical School, University of Sao Paulo, Ribeirao Preto, Brazil; 2 Department of Biomedical Sciences, Cedars-Sinai, Los Angeles, CA, USA; 3 Interuniversity Institute of Bioinformatics in Brussels, Brussels, Belgium; 4 Machine Learning Group, ULB, Brussels, Belgium; 5 Department of Science and Technology, University of Sannio, Benevento, Italy; 6 Biogem, Istituto di Ricerche Genetiche Gaetano Salvatore, Avellino, Italy; 7 Qatar Computing Research Institute (QCRI), HBKU, Doha, Qatar; 8 Department of Neurosurgery, Henry Ford Hospital, Detroit, MI, USA

**Keywords:** Epigenomics, Genomics, Cancer, non-coding, TCGA, ENCODE, Roadmap, Bioinformatics

## Abstract

Biotechnological advances in sequencing have led to an explosion of publicly available data via large international consortia such as The Cancer Genome Atlas (TCGA), The Encyclopedia of DNA Elements (ENCODE), and The NIH Roadmap Epigenomics Mapping Consortium (Roadmap). These projects have provided unprecedented opportunities to interrogate the epigenome of cultured cancer cell lines as well as normal and tumor tissues with high genomic resolution. The Bioconductor project offers more than 1,000 open-source software and statistical packages to analyze high-throughput genomic data. However, most packages are designed for specific data types (e.g. expression, epigenetics, genomics) and there is no one comprehensive tool that provides a complete integrative analysis of the resources and data provided by all three public projects. A need to create an integration of these different analyses was recently proposed. In this workflow, we provide a series of biologically focused integrative analyses of different molecular data. We describe how to download, process and prepare TCGA data and by harnessing several key Bioconductor packages, we describe how to extract biologically meaningful genomic and epigenomic data. Using Roadmap and ENCODE data, we provide a work plan to identify biologically relevant functional epigenomic elements associated with cancer. To illustrate our workflow, we analyzed two types of brain tumors: low-grade glioma (LGG) versus high-grade glioma (glioblastoma multiform or GBM). This workflow introduces the following Bioconductor packages: AnnotationHub, ChIPSeeker, ComplexHeatmap, pathview, ELMER, GAIA, MINET, RTCGAToolbox, TCGAbiolinks.

## Introduction

Cancer is a complex genetic disease spanning multiple molecular events such as point mutations, structural variations, translocations and activation of epigenetic and transcriptional signatures and networks. The effects of these events take place at different spatial and temporal scales with interlayer communications and feedback mechanisms creating a highly complex dynamic system. To gain insight into the biology of tumors most of the research in cancer genomics is aimed at the integration of the observations at multiple molecular scales and the analysis of their interplay. Even if many tumors share similar recurrent genomic events, their relationships with the observed phenotype are often not understood. For example, although we know that the majority of the most aggressive form of brain tumors such as glioma harbor the mutation of a single gene (IDH), the mechanistic explanation of the activation of its characteristic epigenetic and transcriptional signatures are still far to be well characterized. Moreover, network-based strategies have recently emerged as an effective framework for the discovery functional disease drivers that act as main regulators of cancer phenotypes.

Indeed, recent technological developments allowed the deposition of large amounts of genomic and epigenomic data, such as gene expression, DNA methylation, and genomic localization of transcription factors, into freely available public international consortia like The Cancer Genome Atlas ( TCGA), The Encyclopedia of DNA Elements ( ENCODE), and The NIH Roadmap Epigenomics Mapping Consortium ( Roadmap) ^1^. An overview of the three consortia is described below:

- **The Cancer Genome Atlas (TCGA):** The TCGA consortium, which is a National Institute of Health (NIH) initiative, makes publicly available molecular and clinical information for more than 30 types of human cancers including exome (variant analysis), single nucleotide polymorphism (SNP), DNA methylation, transcriptome (mRNA), microRNA (miRNA) and proteome. Sample types available at TCGA are: primary solid tumors, recurrent solid tumors, blood derived normal and tumor, metastatic, and solid tissue normal ^2^.
- **The Encyclopedia of DNA Elements (ENCODE):** Found in 2003 by the National Human Genome Research Institute (NHGRI), the project aims to build a comprehensive list of functional elements that have an active role in the genome, including regulatory elements that govern gene expression. Biosamples includes immortalized cell lines, tissues, primary cells and stem cells ^3^.
- **The NIH Roadmap Epigenomics Mapping Consortium:** This was launched with the goal of producing a public resource of human epigenomic data in order to analyze biology and disease-oriented research. Roadmap maps DNA methylation, histone modifications, chromatin accessibility, and small RNA transcripts in stem cells and primary *ex vivo* tissues ^4, 5^.

Briefly, these three consortia provide large scale epigenomic data onto a variety of microarrays and next-generation sequencing (NGS) platforms. Each consortium encompasses specific types of biological information on specific type of tissue or cell and when analyzed together, it provides an invaluable opportunity for research laboratories to better understand the developmental progression of normal cells to cancer state at the molecular level and importantly, correlate these phenotypes with tissue of origins.

Although there exists a wealth of possibilities ^6^ in accessing cancer associated data, Bioconductor represent the most comprehensive set of open source, updated and integrated professional tools for the statistical analysis of large scale genomic data. Thus, we propose our workflow within Bioconductor to describe how to download, process, analyze and integrate cancer data to understand specific cancer-related specific questions. However, there is no tool that solves the issue of integration in a comprehensive sequence and mutation information, epigenomic state and gene expression within the context of gene regulatory networks to identify oncogenic drivers and characterize altered pathways during cancer progression. Therefore, our workflow presents several Bioconductor packages to work with genomic and epigenomics data.

## Methods

### Access to the data

TCGA data is accessible via the the NCI Genomic Data Commons (GDC) data portal, GDC Legacy Archive and the Broad Institute’s GDAC Firehose. The GDC Data Portal provides access to the subset of TCGA data that has been harmonized against GRCh38 (hg38) using GDC Bioinformatics Pipelines which provides methods to the standardization of biospecimen and clinical data, the re-alignment of DNA and RNA sequence data against a common reference genome build GRCh38, and the generation of derived data. Whereas the GDC Legacy Archive provides access to an unmodified copy of data that was previously stored in CGHub ^7^ and in the TCGA Data Portal hosted by the TCGA Data Coordinating Center (DCC), in which uses as references GRCh37 (hg19) and GRCh36 (hg18).

The previously stored data in CGHub, TCGA Data Portal and Broad Institute’s GDAC Firehose, were provided as different levels or tiers that were defined in terms of a specific combination of both processing level (raw, normalized, integrated) and access level (controlled or open access). Level 1 indicated raw and controlled data, level 2 indicated processed and controlled data, level 3 indicated Segmented or Interpreted Data and open access and level 4 indicated region of interest and open access data. While the TCGA data portal provided level 1 to 3 data, Firehose only provides level 3 and 4. An explanation of the different levels can be found at TCGA Wiki. However, the GDC data portal no longer uses this based classification model in levels. Instead a new data model was created, its documentation can be found in GDC documentation. In this new model, data can be open or controlled access. While the GDC open access data does not require authentication or authorization to access it and generally includes high level genomic data that is not individually identifiable, as well as most clinical and all biospecimen data elements, the GDC controlled access data requires dbGaP authorization and eRA Commons authentication and generally includes individually identifiable data such as low level genomic sequencing data, germline variants, SNP6 genotype data, and certain clinical data elements. The process to obtain access to controlled data is found in GDC web site.

Finally, the data provided by GDC data portal and GDC Legacy Archive can be accessed using Bioconductor package TCGAbiolinks, while the data provided by Firehose can be accessed by Bioconductor package RTCGAToolbox.

The next steps describes how one could use TCGAbiolinks & RTCGAToolbox to download clinical, genomics, transcriptomics, epigenomics data, as well as subtype information and GISTIC results (i.e., identified genes targeted by somatic copy-number alterations (SCNAs) that drive cancer growth). All the data used in this workflow has as reference the Genome Reference Consortium human genome (build 37 - hg19).

***Downloading data from TCGA data portal.*** The Bioconductor package TCGAbiolinks ^8^ has three main functions *GDCquery*, *GDCdownload* and *GDCprepare* that should sequentially be used to respectively search, download and load the data as an R object. *GDCquery* uses GDC API to search the data for a given project and data category and filters the results by samples, sample type, file type and others features if requested by the user. This function returns a object with a summary table with the results found (samples, files and other useful information) and the arguments used in the query. The most important *GDCquery* arguments are *project* which receives a GDC project (TCGA-USC, TCGA-LGG, TARGET-AML, etc), *data.category* which receives a data category (Transcriptome Profiling, Copy Number Variation, DNA methylation, Gene expression, etc), *data.type* which receives a data type (Gene expression quantification, Isoform Expression Quantification, miRNA Expression Quantification, Copy Number Segment, Masked Copy Number Segment, etc), *workflow.type*, which receives a GDC workflow type (HTSeq - Counts, HTSeq - FPKM-UQ, HTSeq - FPKM), *legacy*, which selects to use the legacy database or the harmonized database, *file.type*, which receives a file type for the searches in the legacy database (hg18.seg, hg19.seg, nocnv_,hg18.seg, nocnv_hg19.seg, rsem.genes.results, rsem.genes.normalized_results, etc) and *platform*, which receives a the platform for the searches in the legacy database (HumanMethylation27, Genome_Wide_SNP_6, IlluminaHiSeq_RNASeqV2, etc). A complete list of possible entries for arguments can be found in the TCGAbiolinks vignette. Listing 1 shows an example of this function.

After the search step, the user will be able to download the data using the *GDCdownload* function which can use either the GDC API to download the samples, or the gdc client tools. The downloaded data will be saved in a directory with the project name and a sub-folder with the data.category, for example “TCGA-GBM/DNA_methylation”.

Finally, *GDCprepare* transforms the downloaded data into a summarizedExperiment object ^9^ or a data frame. If *SummarizedExperiment* is set to TRUE, TCGAbiolinks will add to the object sub-type information, which was defined by The Cancer Genome Atlas (TCGA) Research Network reports (the full list of papers can be seen in TCGAquery_subtype section in TCGAbiolinks vignette), and clinical information. Listing 1 shows how to use these functions to download DNA methylation and gene expression data from the GDC legacy database and 2 shows how to download copy number variation from harmonized data portal. Other examples, that access the harmonized data can be found in the TCGAbiolinks vignette.


 
                        1 
                        library
                        (TCGAbiolinks)
 
                        2
 
                        3 
                        # Obs: The data in the legacy database has been aligned to hg19
 
                        4 
                        query.met.gbm 
                        <– 
                        GDCquery(project = 
                        "TCGA–GBM"
                        ,
 
                        5                         
                        legacy = TRUE,
 
                        6                         
                        data
                        .
                        category 
                        = 
                        "DNA methylation"
                        ,
 
                        7                         
                        platform = 
                        "Illumina Human Methylation 450"
                        ,
 
                        8                         
                        barcode = 
                        c
                        (
                        "TCGA–76–4926–01B–01D–1481–05"
                        , 
                        "TCGA–28–5211–01C–11D–1844–05"
                        ))
 
                        9 GDCdownload(query.met.gbm)

                        10

                        11 met.gbm.450 
                        <– 
                        GDCprepare(query = query.met.gbm,

                        12                         
                        save 
                        = TRUE,

                        13                         
                        save
                        .filename = 
                        "gbmDNAmet450k.rda"
                         ,

                        14                         
                        summarizedExperiment = TRUE)

                        15 query.met.lgg 
                        <– 
                        GDCquery(project = 
                        "TCGA–LGG"
                        ,

                        16                              
                        legacy = TRUE,

                        17                              
                        data
                        .
                        category 
                        = 
                        "DNA methylation "
                        ,

                        18                              
                        platform = 
                        "Illumina Human Methylation 450"
                        ,

                        19                              
                        barcode = 
                        c
                        (
                        "TCGA–HT–7879–01A–11D–2399–05"
                        , 
                        "TCGA–HT–8113–01A–11D–2399–05"
                         )
        
                        )

                        20 GDCdownload(query.met.lgg)

                        21 met.lgg.450 
                        <– 
                        GDCprepare(query = query.met.lgg,

                        22                              
                        save 
                        = TRUE,

                        23                              
                        save
                        .filename = 
                        "lggDNAmet450k.rda"
                        ,

                        24                              
                        summarizedExperiment = TRUE)

                        25 met.gbm.lgg 
                        <– 
                        SummarizedExperiment::
                        cbind
                        (met.lgg.450, met.gbm.450)

                        26

                        27

                        28 query.
                        exp
                        .lgg 
                        <– 
                        GDCquery(project = 
                        "TCGA–LGG"
                        ,

                        29                         
                        legacy = TRUE,

                        30                         
                        data
                        .
                        category 
                        = 
                        "Gene expression"
                        ,

                        31                         
                        data
                        .type = 
                        "Gene expression quantification"
                        ,

                        32                         
                        platform = 
                        "Illumina HiSeq"
                        ,

                        33                         
                        file
                        .type = 
                        "results"
                        ,

                        34                         
                        sample
                        .type = 
                        "Primary solid Tumor"
                        )

                        35 GDCdownload(query.
                        exp
                        .lgg)

                        36 
                        exp
                        .lgg 
                        <– 
                        GDCprepare(query = query.
                        exp
                        .lgg, 
                        save 
                        = TRUE, 
                        save
                        .filename = 
                        "lggExp.rda"
                        )

                        37

                        38 query.
                        exp
                        .gbm 
                        <– 
                        GDCquery(project = 
                        "TCGA–GBM"
                        ,

                        39                         
                        legacy = TRUE,

                        40                         
                        data
                        .
                        category 
                        = 
                        "Gene expression"
                        ,

                        41                         
                        data
                        .type = 
                        "Gene expression quantification"
                        ,

                        42                         
                        platform = 
                        "Illumina HiSeq"
                        ,

                        43                         
                        file
                        .type = 
                        "results"
                        ,

                        44                         
                        sample
                        .type = 
                        "Primary solid Tumor"
                        )

                        45 GDCdownload(query.
                        exp
                        .gbm)

                        46 
                        exp
                        .gbm 
                        <– 
                        GDCprepare(query = query.
                        exp
                        .gbm, 
                        save 
                        = TRUE, 
                        save
                        .filename = 
                        "gbmExp.rda"
                        )

                        47 
                        exp
                        .gbm.lgg 
                        <– 
                        SummarizedExperiment::
                        cbind
                        (
                        exp
                        .lgg, 
                        exp
                        .gbm)
                    


**Listing 1. Downloading TCGA DNA methylation and gene expression data from GDC legacy database with TCGAbiolinks**



 
                        1 
                        library
                        (TCGAbiolinks)
 
                        2 
                        #——————————————————————————————————————————————————————————————————————————
 
                        3 
                        #               Data.category: Copy number variation aligned to hg38
 
                        4 
                        #——————————————————————————————————————————————————————————————————————————
 
                        5 
                        query 
                        <– 
                        GDCquery(project = 
                        "TCGA–ACC"
                        ,
 
                        6                     
                        data
                        .
                        category 
                        = 
                        "Copy Number Variation"
                        ,
 
                        7                     
                        data
                        .type = 
                        "Copy Number Segment"
                        ,
 
                        8                     
                        barcode = 
                        c
                        ( 
                        "TCGA–OR–A5KU–01A–11D–A29H–01"
                        , 
                        "TCGA–OR–A5JK–01A–11D–A29H–01"
                        ))
 
                        9 GDCDownload(query)

                        10 
                        data 
                        <– 
                        GDCPrepare(query)

                        11

                        12 
                        query 
                        <– 
                        GDCquery(
                        "TCGA–ACC"
                        ,

                        13                     
                        "Copy Number Variation"
                        ,

                        14                     
                        data
                        .type = 
                        "Masked Copy Number Segment"
                        ,

                        15                     
                        sample
                        .type = 
                        c
                        (
                        "Primary solid Tumor"
                        )) 
                        # see the barcodes with query
                        $
                        results[[1]]
                        $
                        cases

                        16 
                        GDCDownload(query)

                        17 
                        data 
                        <– 
                        GDCPrepare(query)
                    


**Listing 2. Downloading TCGA copy number variation data from GDC harmonized database with TCGAbiolinks**

If a summarizedExperiment object was chosen, the data can be accessed with three different accessors: *assay* for the data information, *rowRanges* to gets the range of values in each row and *colData* to get the sample information (patient, batch, sample type, etc) ^9, 10^. An example is shown in Listing 3.



                        1 
                        library
                        (summarizedExperiment)
2 
                        # get expression matrix

                        3 
                        data <– 
                        assay(
                        exp
                        .gbm.lgg)
4
5 
                        # get genes information

                        6 genes.info 
                        <– 
                        rowRanges(
                        exp
                        .gbm.lgg)
7
8 
                        # get sample information

                        9 
                        sample
                        .info 
                        <– 
                        colData(
                        exp
                        .gbm.lgg)



**Listing 3. summarizedExperiment accessors**

The clinical data can be obtained using TCGAbiolinks through two methods. The first one will download only the indexed GDC clinical data which includes diagnoses (vital status, days to death, age at diagnosis, days to last follow up, days to recurrence), treatments (days to treatment, treatment id, therapeutic agents, treatment intent type), demographic (gender, race, ethnicity) and exposures (cigarettes per day, weight, height, alcohol history) information. This indexed clinical data can be obtained using the function *GDCquery_clinical* which can be used as described in Listing 4. This function has two arguments *project* ("TCGA-GBM","TARGETAML", etc) and *type* ("Clinical" or "Biospecimen"). The second method will download the xml files with all clinical data for the patient and retrieve the desired information from it. This will give access to all clinical data available which includes patient (tumor tissue site, histological type, gender, vital status, days to birth, days to last follow up, etc), drug (days to drug therapy start, days to drug therapy end, therapy types, drug name), radiation (days to radiation therapy start, days to radiation therapy end, radiation type, radiation dosage), new tumor event (days to new tumor event after initial treatment, new neoplasm event type, additional pharmaceutical therapy), follow up (primary therapy outcome success, follow up treatment success, vital status, days to last follow up, date of form completion), stage event (pathologic stage, tnm categories), admin (batch number, project code, disease code, Biospecimen Core Resource).


 
                        1 
                        # get indexed clinical patient data for GBM samples
 
                        2 gbm
                        _
                        clin 
                        <– 
                        GDCquery
                        _
                        clinic(project = 
                        "TCGA–GBM"
                        , type = 
                        "Clinical"
                        )
 
                        3
 
                        4 
                        # get indexed clinical patient data for LGG samples
 
                        5 lgg
                        _
                        clin 
                        <– 
                        GDCquery
                        _
                        clinic(project = 
                        "TCGA–LGG"
                        , type = 
                        "Clinical"
                        )
 
                        6
 
                        7 
                        # Bind the results, as the columns might not be the same,
 
                        8 
                        # we will will plyr rbind.fill, to have all columns from both files
 
                        9 clinical 
                        <– 
                        plyr::
                        rbind
                        .fill(gbm
                        _
                        clin,lgg
                        _
                        clin)
10
11 
                        # if barcode is not set, it will consider all samples.

                        12 
                        # We only set it to make the example faster

                        13 query.clin
                         <– 
                        GDCquery(project = 
                        "TCGA–GBM"
                        ,
14 			     
                        data
                        .
                        category 
                        = 
                        "Clinical"
                        ,
15 		          barcode = 
                        c
                        (
                        "TCGA–08–0516"
                        ,
                        "TCGA–02–0317"
                        ))

                        16 GDCDownload(query.clin)
17 clinical.patient 
                        <– 
                        GDCPrepare
                        _
                        clinic(query,
                        "patient"
                        )
18 clinical.drug 
                        <– 
                        GDCPrepare
                        _
                        clinic(query,
                        "drug"
                        )
19 clinical.radiation 
                        <– 
                        GDCPrepare
                        _
                        clinic(query,
                        "radiation"
                        )
20 clinical.admin 
                        <– 
                        GDCPrepare
                        _
                        clinic(query,
                        "admin"
                        )
21 clinical.followup 
                        <– 
                        GDCPrepare
                        _
                        clinic(query,
                        "follow
                        _
                        up"
                        )
22 clinical.nte 
                        <– 
                        GDCPrepare
                        _
                        clinic(query, 
                        "new
                        _
                        tumor
                        _
                        event"
                        )
23 clinical.stage 
                        <– 
                        GDCPrepare
                        _
                        clinic(query,
                        "stage
                        _
                        event"
                        )
                    


**Listing 4. Downloading clinical data with TCGAbiolinks**

Mutation information is stored in two types of Mutation Annotation Format (MAF): Protected and Somatic (or Public) MAF files, which are derived from the GDC annotated VCF files. Annotated VCF files often have variants reported on multiple transcripts whereas the protected MAF (*protected.maf) only reports the most critically affected one and the Somatic MAFs (*somatic.maf) are further processed to remove low quality and potential germline variants. To download Somatic MAFs data using TCGAbiolinks, *GDCquery_maf* function is provided (see Listing 5).


 
                        1 
                        mutation 
                        <– 
                        GDCquery
                        _
                        Maf(tumor = 
                        "ACC"
                        , pipelines = 
                        "mutect2"
                        )
                    


**Listing 5. Downloading mutation data with TCGAbiolinks**

Finally, the Cancer Genome Atlas (TCGA) Research Network has reported integrated genome-wide studies of various diseases (ACC ^11^, BRCA ^12^, COAD ^13^, GBM ^14^, HNSC ^15^, KICH ^16^, KIRC ^17^, KIRP ^18^, LGG ^14^, LUAD ^19^, LUSC ^20^, PRAD ^21^, READ ^13^, SKCM ^22^, STAD ^23^, THCA ^24^ and UCEC ^23^) which classified them in different subtypes. This classification can be retrieved using the *TCGAquery_subtype* function or by accessing the samples information in the SummarizedExperiment object that created by the *GDCprepare* function.


 
                        1 
                        gbm.subtypes 
                        <– 
                        TCGAquery
                        _
                        subtype(tumor = 
                        "gbm"
                        )
 
                        2 
                        brca.subtypes 
                        <– 
                        TCGAquery
                        _
                        subtype(tumor = 
                        "brca"
                        )
                    


**Listing 6. Accessing subtype information retrieved from TCGA papers**

***Downloading data from Broad TCGA GDAC.*** The Bioconductor package RTCGAToolbox ^25^ provides access to Firehose Level 3 and 4 data through the function *getFirehoseData*. The following arguments allows users to select the version and tumor type of interest:

• dataset - Tumor to download. A complete list of possibilities can be viewed with *getFirehoseDatasets* function.
• runDate - Stddata run dates. Dates can be viewed with *getFirehoseRunningDates* function.
• gistic2_Date - Analyze run dates. Dates can viewed with *getFirehoseAnalyzeDates* function.

These arguments can be used to select the data type to download: RNAseq_Gene, Clinic, miRNASeq_Gene, ccRNAseq2_Gene_Norm, CNA_SNP, CNV_SNP, CNA_Seq, CNA_CGH, Methylation, Mutation, mRNA_Array, miRNA_Array, and RPPA.

By default, RTCGAToolbox allows users to download up to 500 MB worth of data. To increase the size of the download, users are encouraged to use *fileSizeLimit* argument. An example is found in Listing 7. The *getData* function allow users to access the downloaded data (see lines 22–24 of Listing 7) as a S4Vector object.


 
                        1 
                        library
                        (RTCGAToolbox)
 
                        2
 
                        3 
                        # Get the last run dates
 
                        4 lastRunDate 
                        <- 
                        getFirehoseRunningDates()[1]
 
                        5 lastAnalyseDate 
                        <- 
                        getFirehoseAnalyzeDates(1)
 
                        6
 
                        7 
                        # get DNA methylation data, RNAseq2 and clinical data for LGG
 
                        8 lgg.
                        data <- 
                        getFirehoseData(dataset = 
                        "LGG"
                        ,
 
                        9			       gistic2
                        _
                        Date = getFirehoseAnalyzeDates(1), runDate = lastRunDate,
10			       Methylation = TRUE, RNAseq2
                        _
                        Gene
                        _
                        Norm = TRUE, Clinic = TRUE,
11			       Mutation = T,
12			       fileSizeLimit = 10000)
13
14 
                        # get DNA methylation data, RNAseq2 and clinical data for GBM

                        15 gbm.
                        data <- 
                        getFirehoseData(dataset = 
                        "GBM"
                        ,
16			       runDate = lastDate, gistic2
                        _
                        Date = getFirehoseAnalyzeDates(1),
17			       Methylation = TRUE, Clinic = TRUE, RNAseq2
                        _
                        Gene
                        _
                        Norm = TRUE,
18			       fileSizeLimit = 10000)
19
20 
                        # To access the data you should use the getData function

                        21 
                        # or simply access with @ (for example gbm.data@Clinical)

                        22 gbm.mut 
                        <- 
                        getData(gbm.
                        data
                        ,
                        "Mutations"
                        )
23 gbm.clin 
                        <- 
                        getData(gbm.
                        data
                        ,
                        "Clinical"
                        )
24 gbm.gistic 
                        <- 
                        getData(gbm.
                        data
                        ,
                        "GISTIC"
                        )
                    


**Listing 7. Downloading TCGA data files with RTCGAToolbox**

Finally, using RTCGAToolbox the user can retrieve CNV level 4 data, including the amplified or deleted genes identified by GISTIC which rates each segment based on the frequency of occurrence combined with the amplitude of aberration, using a permutation test to assess the statistical significance. Among GISTIC results there are two tables that can be accessed by RTCGAToolbox:

- A gene-level table of copy number values for all samples. The copy number values in the table are in units of (copy number -2), so that no amplification or deletion is 0, genes with amplifications have positive values, and genes with deletions are negative values. The data are converted from marker level to gene level using the extreme method: a gene is assigned the greatest amplification or the least deletion value among the markers it covers.
- A gene-level table of discrete amplification and deletion indicators for all samples. A table value of 0 means no amplification or deletion above the threshold (diploid normal copy). Amplifications are positive numbers: 1 means amplification above the amplification threshold (low-level gain, 1 extra copy); 2 means amplifications larger to the arm level amplifications observed for the sample (high-level amplification, 2 or more extra copies). Deletions are represented by negative table values: -1 represents deletion beyond the threshold (possibly a heterozygous deletion); -2 means deletions greater than the minimum arm-level deletion observed for the sample (possibly a homozygous deletion).

More details about the GISTIC algorithm and its use are described in 26– 28. (see Listing 8).


 
                        1 
                        # Download GISTIC results
 
                        2 gistic 
                        <- 
                        getFirehoseData(
                        "GBM"
                        ,gistic2
                        _
                        Date =
                        "20141017" 
                        )
 
                        3
 
                        4 
                        # get GISTIC results
 
                        5 gistic.allbygene 
                        <- 
                        gistic@GISTIC@AllByGene
 
                        6 gistic.thresholedbygene 
                        <- 
                        gistic@GISTIC@ThresholedByGene
                    


**Listing 8. Using RTCGAToolbox to get the GISTIC results**

### Genomic analysis

Copy number variations (CNVs) have a critical role in cancer development and progression. A chromosomal segment can be deleted or amplified as a result of genomic rearrangements, such as deletions, duplications, insertions and translocations. CNVs are genomic regions greater than 1 kb with an alteration of copy number between two conditions (e.g., Tumor *versus* Normal).

TCGA collects copy number data and allows the CNV profiling of cancer. Tumor and paired-normal DNA samples were analyzed for CNV detection using microarray and sequencing-based technologies. Level 3 processed data are the aberrant regions along the genome resulting from CNV segmentation, and they are available for all copy number technologies.

In this section, we will show how to analyze CNV level 3 data from TCGA to identify recurrent alterations in cancer genome. We analyzed GBM and LGG segmented CNV from SNP array (Affymetrix Genome-Wide Human SNP Array 6.0).

***Pre-Processing data.*** The only CNV platform available for both LGG and GBM in TCGA is "Affymetrix Genome-Wide Human SNP Array 6.0". Using TCGAbiolinks, we queried for CNV SNP6 level 3 data for primary solid tumor samples in the legacy database. Data for selected samples were downloaded and prepared in two separate rse objects (RangedSummarizedExperiment).


 
 
                        1 
                        #############################
 
                        2 
                        ## CNV data pre–processing ##
 
                        3 
                        #############################
 
                        4 
                        library
                        (TCGAbiolinks)
 
                        5
 
                        6 
                        query.lgg.nocnv 
                        <– 
                        GDCquery(project = 
                        "TCGA–LGG"
                        ,
 
                        7                                
                        data
                        .
                        category 
                        = 
                        "Copy number variation"
                        ,
 
                        8                                
                        legacy = TRUE,
 
                        9                                
                        file
                        .type = 
                        "nocnv
                        _
                        hg19.seg"
                        ,

                        10                                
                        sample
                        .type = 
                        c
                        (
                        Primary solid Tumor"
                        ))

                        11 
                        GDCdownload(query.lgg.nocnv)

                        12 
                        lgg.nocnv 
                        <– 
                        GDCprepare(query.lgg.nocnv, 
                        save 
                        = TRUE, 
                        save
                        .filename = 
                        "LGGnocnvhg19.rda"
                        )

                        13

                        14 
                        query.gbm.nocnv 
                        <– 
                        GDCquery(project = 
                        "TCGA–GBM"
                        ,

                        15                                
                        data
                        .
                        category 
                        = 
                        "Copy number variation"
                        ,

                        16                                
                        legacy = TRUE,

                        17                                
                        file
                        .
                        type = 
                        "nocnv
                        _
                        hg19.seg"
                        ,

                        18                                
                        sample
                        .type = 
                        c
                        (
                        "Primary solid Tumor"
                        ))

                        19 
                        GDCdownload(query.gbm.nocnv)

                        20 
                        gbm.nocnv 
                        <– 
                        GDCprepare (query.gbm.nocnv, 
                        save 
                        = TRUE, 
                        save
                        .file name = 
                        "GBMnocnvhg19.rda"
                        )



**Listing 9. Searching, downloading and preparing CNV data with TCGAbiolinks**

***Identification of recurrent CNV in cancer.*** Cancer related CNV have to be present in many of the analyzed genomes. The most significant recurrent CNV were identified using GAIA ^29^, an iterative procedure where a statistical hypothesis framework is extended to take into account within-sample homogeneity. GAIA is based on a conservative permutation test allowing the estimation of the probability distribution of the contemporary mutations expected for non-driver markers. Segmented data retrieved from TCGA were used to generate a matrix including all needed information about the observed aberrant regions. Furthermore, GAIA requires genomic probes metadata (specific for each CNV technology), that can be downloaded from broadinstitute website.



  
                        1 
                        ###############################
  
                        2 
                        ## CNV  data  pre–processing ##
  
                        3 
                        ###############################
  
                        4 
                        library
                        (TCGAbiolinks)
  
                        5 
                        library
                        (downloader)
  
                        6 
                        library
                        (readr)
  
                        7 
                        library
                        (gaia)
  
                        8
  
                        9 gaiaCNVplot 
                        <–  function
                        (calls, cancer=NULL, threshold=0.01)
 
                        10 {
 
                        11     Calls 
                        <– 
                        calls[
                        order
                        (calls[,
                        "Region Start[bp]"
                        ]),]
 
                        12     Calls 
                        <– 
                        Calls[
                        order
                        (Calls[,
                        "Chromosome"
                        ]),]
 
                        13      
                        rownames
                        (Calls) 
                        <– 
                        NULL
 
                        14     Chromo 
                        <– 
                        Calls[,
                        "Chromosome"
                        ]
 
                        15     Gains 
                        <–  apply
                        (Calls,1, 
                        function
                        (x) 
                        ifelse
                        (x[
                        "Aberration Kind"
                        ]==1, x[
                        "score"
                        ], 0))
 
                        16     Losses 
                        <– apply
                        (Calls,1,
                        function
                        (x) 
                        ifelse
                        (x[
                        "Aberration Kind"
                        ]==0, x[
                        "score"
                        ], 0))
 
                        17      
                        plot
                        (Gains, ylim = 
                        c
                        (–
                        max
                        (Calls[,
                        "score"
                        ]+2), 
                        max
                        (Calls[,
                        "score"
                        ]+2)), type  = 
                        "h"
                        ,
 
                        18           
                        col 
                         = 
                        "red"
                        , xlab = 
                        "Chromosome"
                        , ylab = 
                        "Score"
                        ,
 
                        19           
                        #main = paste("Recurrent Copy Number Variations",cancer, sep =" – "),
 
                        20	     xaxt = 
                        "n"
                        )
 
                        21	 
                        points
                        (–(Losses), type = 
                        "h"
                        , 
                        col 
                        = 
                        "blue"
                        )
 
                        22	 
                        abline
                        (h = 0, cex = 4)
 
                        23	 
                        abline
                        (h = –
                        log10
                        (threshold), 
                        col 
                        = 
                        "orange"
                        , 
                        cex = 4, main=
                        "test"
                        )
 
                        24	 
                        abline
                        (h = 
                        log10
                        (threshold), 
                        col 
                        = 
                        "orange"
                        , cex = 4, main=
                        "test"
                        )
 
                        25     uni.chr 
                        <– unique
                        (Chromo)
 
                        26     temp 
                        <– rep
                        (0, 
                        length
                        (uni.chr))
 
                        27	 
                        for 
                        (i in 1 :
                        length
                        (uni.chr)) {
 
                        28         temp[i]   
                        <– max
                        (
                        which
                        (uni.chr[i] == Chromo)) 
 
                        29     }
 
                        30	 
                        for 
                        (i in 1:
                        length
                        (temp)) {
 
                        31	      
                        abline
                        (v = temp[i], 
                        col 
                        = 
                        "black"
                        , lty = 
                        "dashed"
                        , )
 
                        32     }
 
                        33     nChroms 
                        <– length
                        (uni.chr)
 
                        34     begin 
                        <– c
                        ()
 
                        35	 
                        for 
                        (d in 1: nChroms) {
 
                        36	     chrom 
                        <– sum
                        (Chromo == uni.chr[d])
 
                        37          begin 
                        <– append
                        (begin, chrom)
 
                        38     }
 
                        39     temp2 
                        <– rep
                        (0, nChroms)
 
                        40	 
                        for
                        (i in 1:nChroms) {
 
                        41         
                        if 
                        (i == 1) {
 
                        42	       temp2[1] 
                        <–
                        (begin[1] 
                        * 
                        0.5)
 
                        43	   }
 
                        44	     
                        else  if
                        (i > 1) {
 
                        45	        temp2[i] 
                        <– 
                        temp[i – 1] + (begin[i] 
                        * 
                        0.5)
 
                        46
                        	   }
 
                        47	}
 
                        48	uni.chr[uni.chr ==23] 
                        <– 
                        "X"
 
                        49	uni.chr[uni.chr ==24] 
                        <– 
                        "Y"
 
                        50	 
                        for 
                        (i in 1:
                        length
                        (temp)) {
 
                        51	      
                        axis
                        (1, at = temp2[i], 
                        labels 
                        = uni.chr[i], cex.
                        axis 
                        = 1) 
 
                        52     }
 
                        53	 
                        legend
                        (x=1, y=
                        max
                        (Calls[,
                        "score"
                        ]+2), y.intersp=0.8, 
                        c
                        (
                        "Amp" 
                        ), pch=15, 
                        col
                        =
                        c
                        (
                        "red"
                        ),  
                        text
                        .font=3)
 
                        54	 
                        legend
                        (x=1,y=–
                        max
                        (Calls[,
                        "score"
                        ]+0.5), y.intersp=0.8, 
                        c
                        (
                        "Del"
                        ), pch=15, 
                        col
                        =
                        c
                        (
                        "blue"
                        ), 
                        text
                        .font=3)
 
                        55 }
 
                        56
 
                        57
 
                        58 
                        for
                        (cancer in 
                        c
                        (
                        "LGG"
                        , 
                        "GBM"
                        )){
 
                        59	message(paste0(
                        "Starting"
                        , cancer))
 
                        60	 
                        # Prepare CNV matrix
 
                        61	cnvMatrix 
                        <– get
                        (
                        load
                        (paste0 (cancer,
                        "nocnvhg19.rda"
                        )))
 
                        62
 
                        63	 
                        # Add label (0 for loss, 1 for gain)
 
                        64	cnvMatrix 
                        <– cbind
                        (cnvMatrix, Label=NA)
 
                        65	cnvMatrix[cnvMatrix[,
                        "Segment
                        _
                        Mean"
                        ] < –0.3, 
                        "Label" 
                        ] 
                        <– 
                        0
 
                        66	cnvMatrix[cnvMatrix[,
                        "Segment
                        _
                        Mean"
                        ] > 0.3,
                        "Label"
                        ] 
                        <– 
                        1
 
                        67	cnvMatrix 
                        <– 
                        cnvMatrix[
                        !is
                        .
                        na
                        (cnvMatrix
                        $
                        Label),]
 
                        68
 
                        69	 
                        # Remove " Segment
                        _
                        Mean" and change col.names
 
                        70	cnvMatrix 
                        <–
                        cnvMatrix[,–6]
 
                        71	 
                        colnames
                        (cnvMatrix) 
                        <– c
                        ( 
                        "Sample.Name"
                        , 
                        "Chromosome"
                        , 
                        "Start"
                        , 
                        "End"
                        , 
                        "Num.of.Markers"
                        , 
                        "Aberration"
                        )
 
                        72
 
                        73	 
                        # Substitute Chromosomes "X" and "Y" with "23" and "24"
 
                        74	xidx 
                        <– which
                        (cnvMatrix
                        $
                        Chromosome==
                        "X"
                        )
 
                        75	yidx 
                        <– which
                        (cnvMatrix
                        $
                        Chromosome==
                        "Y"
                        )
 
                        76     cnvMatrix[xidx,
                        "Chromosome"
                        ] 
                        <– 
                        23
 
                        77     cnvMatrix[yidx,
                        "Chromosome"
                        ] 
                        <- 
                        24
 
                        78     cnvMatrix
                        $
                        Chromosome 
                        <- sapply
                        (cnvMatrix
                        $
                        Chromosome,
                        as
                        .
                        integer
                        )
 
                        79
 
                        80      
                        # Recurrent CNV identification with GAIA
 
                        81
 
                        82      
                        # Retrieve probes meta file from broadinstitute website
 
                        83      
                        # Recurrent CNV identification with GAIA
 
                        84     gdac.root 
                        <- 
                        "ftp:
                        //
                        ftp.broadinstitute.org
                        /
                        pub
                        /
                        GISTIC2.0
                        /
                        hg19
                        _
                        support
                        /
                        "
 
                        85      
                        file <- 
                        paste0(gdac.root, 
                        "genome.info.6.0
                        _
                        hg19.na31
                        _
                        minus
                        _
                        frequent
                        _
                        nan
                        _
                        probes
                        _
                        sorted
                        _
                        2.1.txt"
                        )
 
                        86      
                        # Retrieve probes meta file from broadinstitute website
 
                        87      
                        if
                        (
                        !file.
                        exists
                        (
                        basename
                        (
                        file
                        ))) 
                        download
                        (
                        file
                        , 
                        basename
                        (
                        file
                        ))
 
                        88     markersMatrix 
                        <- 
                        readr::
                        read_
                        tsv(
                        basename
                        (
                        file
                        ), 
                        col_names 
                        = FALSE, 
                        col_
                        types = 
                        "ccn"
                        , progress = TRUE)
 
                        89      
                        colnames
                        (markersMatrix) 
                        <- c
                        (
                        "Probe.Name"
                        , 
                        "Chromosome"
                        , 
                        "Start"
                        )
 
                        90      
                        unique
                        (markersMatrix
                        $
                        Chromosome)

                         91     xidx 
                        <- which
                        (markersMatrix
                        $
                        Chromosome==
                        "X"
                        )
 
                        92     yidx 
                        <- which
                        (markersMatrix
                        $
                        Chromosome==
                        "Y"
                        )
 
                        93     markersMatrix[xidx,
                        "Chromosome"
                        ] 
                        <- 
                        23
 
                        94     markersMatrix[yidx,
                        "Chromosome"
                        ] 
                        <- 
                        24
 
                        95     markersMatrix
                        $
                        Chromosome 
                        <- sapply
                        (markersMatrix
                        $
                        Chromosome,
                        as
                        .
                        integer
                        )
 
                        96     markerID 
                        <- apply
                        (markersMatrix,1,
                        function
                        (x) paste0(x[2],
                        ":",
                        x[3]))
 
                        97      
                        print
                        (
                        table
                        (
                        duplicated
                        (markerID)))
 
                        98      
                        ## FALSE    TRUE
 
                        99      
                        ## 1831041     186

                        100      
                        # There are 186 duplicated markers

                        101      
                        print
                        (
                        table
                        (
                        duplicated
                        (markersMatrix
                        $
                        Probe.Name)))

                        102      
                        ## FALSE

                        103      
                        ## 1831227

                        104      
                        #  ... with different names
                        !

                        105      
                        # Removed duplicates

                        106     markersMatrix 
                        <- 
                        markersMatrix[-
                        which
                        (
                        duplicated
                        (markerID)),]

                        107      
                        # Filter markersMatrix for common CNV

                        108     markerID 
                        <- apply
                        (markersMatrix,1,
                        function
                        (x) paste0(x[2],
                        ":",
                        x[3]))

                        109

                        110      
                        file <- 
                        paste0(gdac.root, 
                        "CNV.hg19.bypos.111213.txt"
                        )

                        111      
                        if
                        (
                        !file.
                        exists
                        (
                        basename
                        (
                        file
                        ))) 
                        download
                        (
                        file, 
                        basename
                        (
                        file
                        ))

                        112     commonCNV 
                        <- 
                        readr::
                        read_
                        tsv(
                        basename
                        (
                        file
                        ), progress = TRUE)

                        113     commonID 
                        <- 
                        apply
                        (commonCNV,1,
                        function
                        (x) paste0(x[2],
                        ":",
                        x[3]))

                        114      
                        print
                        (
                        table
                        (commonID %in% markerID))

                        115      
                        print
                        (
                        table
                        (markerID %in% commonID))

                        116     markersMatrix
                        _
                        fil 
                        <- 
                        markersMatrix[
                        !
                        markerID %in% commonID,]

                        117

                        118     markers_
                        obj 
                        <– load_
                        markers(
                        as
                        .
                        data
                        .
                        frame
                        (markersMatrix
                        _
                        fil))

                        119     nbsamples 
                        <– length
                        (
                        get
                        (paste0(
                        "query."
                        ,tolower(cancer),
                        ".nocnv"
                        ))
                        $
                        results[[1]]
                        $
                        cases)

                        120     cnv
                        _
                        obj 
                        <– load_
                        cnv(cnvMatrix, markers
                        _
                        obj, nbsamples)

                        121     results 
                        <– 
                        runGAIA(cnv
                        _
                        obj,

                        122   			   markers
                        _
                        obj,

                        123   			   output
                        _file_
                        name=paste0(
                        "GAIA
                        _
                        "
                        ,cancer,
                        "
                        _
                        flt.txt"
                        ),

                        124   	        aberrations = –1,

                        125   			   chromosomes = –1,

                        126   	        num
                        _
                        iterations = 10,

                        127   	        threshold = 0.25)

                        128

                        129      
                        # Set q–value threshold

                        130     threshold 
                        <– 
                        0.0001

                        131

                        132      
                        # Plot the results

                        133     RecCNV 
                        <– t
                        (
                        apply
                        (results,1,
                        as
                        .
                        numeric
                        ))

                        134      
                        colnames
                        (RecCNV) 
                        <– colnames
                        (results)

                        135     RecCNV 
                        <– cbind
                        (RecCNV, score=0)

                        136     minval 
                        <– format
                        (
                        min
                        (RecCNV[RecCNV[,
                        "q–value"
                        ]
                        !=
                        0,
                        "q–value"
                        ]),scientific =FALSE)

                        137     minval 
                        <– substring
                        (minval,1, 
                        nchar
                        (minval)–1)

                        138     RecCNV[RecCNV[,
                        "q–value"
                        ]==0,
                        "q–value"
                        ] 
                        <– as
                        .
                        numeric
                        (minval)

                        139     RecCNV[,
                        "score"
                        ] 
                        <– sapply
                        (RecCNV[,
                        "q–value"
                        ],
                        function
                        (x) –
                        log10
                        (
                        as
                        .
                        numeric
                        (x)))

                        140     RecCNV[RecCNV[,
                        "q–value"
                        ]==
                        as
                        .
                        numeric
                        (minval),]

                        141

                        142     gaiaCNVplot(RecCNV,cancer,threshold)

                        143      
                        save
                        (results, RecCNV, threshold, 
                        file 
                        = paste0(cancer,
                        "
                        _
                        CNV
                        _
                        results.rda"
                        ))

                        144     message(paste0(
                        "Results saved as:"
                        , cancer,
                        "
                        _
                        CNV
                        _
                        results.rda"
                        ))

                        145 }



**Listing 10. Recurrent CNV identification in cancer with GAIA**

**Figure 1.**
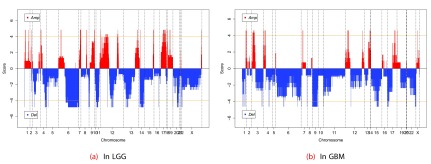
Recurrent CNV (|score threshold| = 4).

Recurrent amplifications and deletions were identified for both LGG ( Figure 1a) and GBM ( Figure 1b), and represented in chromosomal overview plots by a statistical score (– *log*_10_ *corrected p-value* for amplifications and *log*_10_ *corrected p-value* for deletions). Genomic regions identified as significantly altered in copy number ( *corrected p-value* < 10 ^–4^) were then annotated to report amplified and deleted genes potentially related with cancer.

***Gene annotation of recurrent CNV.*** The aberrant recurrent genomic regions in cancer, as identified by GAIA, have to be annotated to verify which genes are significantly amplified or deleted. Using biomaRt we retrieved the genomic ranges of all human genes and we compared them with significant aberrant regions to select full length genes. An example of the result is shown in Table 1.

**Table 1.** Chromosome 20 recurrent deleted genes in LGG.

	GeneSymbol	Aberration	q-value	AberrantRegion	GeneRegion
1	EIF4E2P1	Del	5.74967741935484e-05	20:20540891-21005246	20:20659710-20659964
2	LLPHP1	Del	5.74967741935484e-05	20:20540891-21005246	20:20721187-20721879
3	RN7SL607P	Del	5.74967741935484e-05	20:20540891-21005246	20:20738433-20738731
4	MRPS11P1	Del	5.74967741935484e-05	20:20540891-21005246	20:20854121-20854642
5	RPL24P2	Del	5.74967741935484e-05	20:21091497-21220212	20:21114723-21115197


 
                        1 
                        ##############################
 
                        2 
                        ## Recurrent CNV annotation ##
 
                        3 
                        ##############################
 
                        4 
                        library
                        (biomaRt)
 
                        5 
                        library
                        (GenomicRanges)
 
                        6
 7 mart 
                        <– 
                        useMart(biomart=
                        "ensembl"
                        , dataset=
                        "hsapiens
                        _
                        gene
                        _
                        ensembl"
                        )
 8 genes 
                        <– 
                        getBM(
                        attributes 
                        = 
                        c
                        (
                        "hgnc
                        _
                        symbol"
                        , 
                        "chromosome
                        _
                        name"
                        ,
                        "start
                        _
                        position"
                        ,
                        "end
                        _
                        position"
                        ),
 9	    mart=mart)
10 genes 
                        <– 
                        genes[genes[,1]
                        !=
                        "" 
                        & 
                        genes[,2]%in%
                        c
                        (1:22,
                        "X"
                        ,
                        "Y"
                        ),]
11 xidx 
                        <– which
                        (genes[,2]==
                        "X"
                        )
12 yidx 
                        <– which
                        (genes[,2]==
                        "Y"
                        
                        )

                        13 genes[xidx, 2] 
                        <– 
                        23
14 genes[yidx, 2] 
                        <– 
                        24
15 genes[,2] 
                        <– sapply
                        (genes[,2],
                        as
                        .
                        integer
                        )
16 genes 
                        <– 
                        genes[
                        order
                        (genes[,3]),]
17 genes 
                        <– 
                        genes[
                        order
                        (genes[,2]),]
18 
                        colnames
                        (genes) 
                        <– c
                        (
                        "GeneSymbol"
                        ,
                        "Chr"
                        ,
                        "Start"
                        ,
                        "End"
                        )
19 genes
                        _
                        GR 
                        <– 
                        makeGRangesFromDataFrame(genes,keep.extra.columns = TRUE)

                        20
21 
                        for
                        (cancer in 
                        c
                        (
                        "LGG"
                        ,
                        "GBM"
                        )){
22      
                        load
                        (paste0(cancer,
                        "
                        _
                        CNV
                        _
                        results.rda"
                        ))

                        23     sCNV 
                        <– 
                        RecCNV[RecCNV[,
                        "q–value"
                        ]<=threshold,
                        c
                        (1:4,6)]
24     sCNV 
                        <– 
                        sCNV[
                        order
                        (sCNV[,3]),]
25     sCNV 
                        <– 
                        sCNV[
                        order
                        (sCNV[,1]),]
26      
                        colnames
                        (sCNV) 
                        <– c
                        (
                        "Chr"
                        ,
                        "Aberration"
                        ,
                        "Start"
                        ,
                        "End"
                        ,
                        "q–value"
                        )
27     sCNV
                        _
                        GR 
                        <– 
                        makeGRangesFromDataFrame(sCNV,keep.extra.columns = TRUE)
28
29     hits 
                        <– 
                        findOverlaps(genes
                        _
                        GR, sCNV
                        _
                        GR, type=
                        "within"
                        )
30     sCNV
                        _
                        ann 
                        <– cbind
                        (sCNV[subjectHits(hits),],genes[queryHits(hits),])
31     AberrantRegion 
                        <– 
                        paste0(sCNV
                        _
                        ann[,1],
                        ":"
                        ,sCNV
                        _
                        ann[,3],
                        "–"
                        ,sCNV
                        _
                        ann[,4])
32     GeneRegion 
                        <– 
                        paste0(sCNV
                        _
                        ann[,7],
                        ":"
                        ,sCNV
                        _
                        ann[,8],
                        "–"
                        ,sCNV
                        _
                        ann[,9])
33     AmpDel
                        _
                        genes 
                        <– cbind
                        (sCNV
                        _
                        ann[,
                        c
                        (6,2,5)],AberrantRegion,GeneRegion)
34     AmpDel
                        _
                        genes[AmpDel
                        _
                        genes[,2]==0,2] 
                        <– 
                        "Del"

                        35     AmpDel
                        _
                        genes[AmpDel
                        _
                        genes[,2]==1,2] 
                        <– 
                        "Amp"

                        36      
                        rownames
                        (AmpDel
                        _
                        genes) 
                        <– 
                        NULL
37
38      
                        save
                        (RecCNV, AmpDel
                        _
                        genes, 
                        file 
                        = paste0(cancer,
                        "
                        _
                        CNV
                        _
                        results.rda"
                        ))
39 }
                    


**Listing 11. Gene annotation of recurrent CNV**

**Figure 2.**
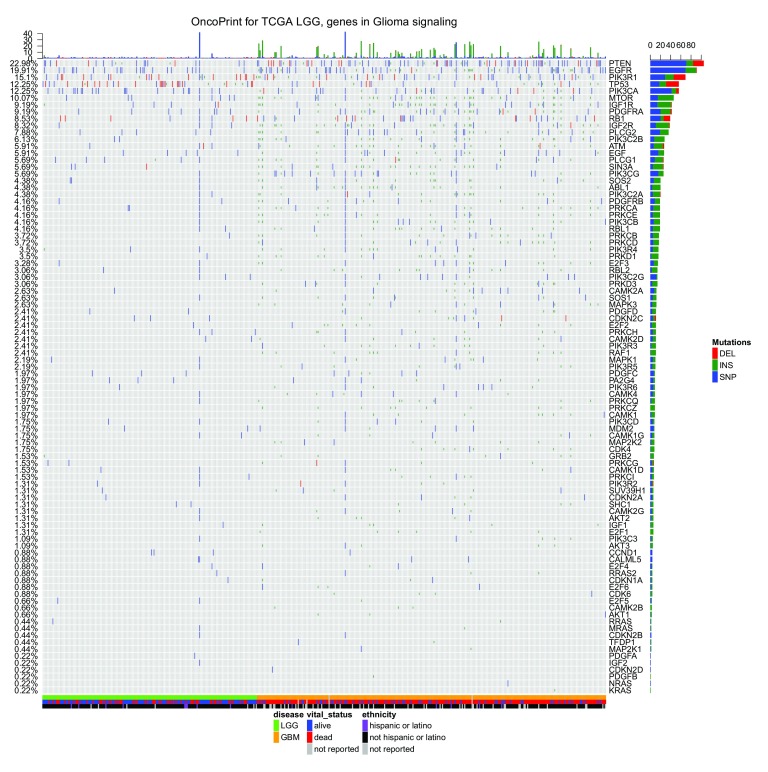
Oncoprint for LGG samples. Blue defines SNP, green defines insertions and red defines deletions. The upper barplot shows the number of these genetic mutations for each patient, while the right barplot shows the number of genetic mutations for each gene. The bottom bar shows the group of each sample.

***Visualizing multiple genomic alteration events***. In order to visualize multiple genomic alteration events we recommend using OncoPrint plot which is provided by Bioconductor package complexHeatmap ^30^. The Listing 12 shows how to download mutation data using *GDCquery_maf* (line 4), then we filtered the genes to obtain genes with mutations found among glioma specific pathways (lines 6 – 12). The following steps prepared the data into a matrix to fit *oncoPrint* function. We defined SNPs as blue, insertions as green and deletions as red. The upper barplot indicates the number of genetic mutation per patient, while the right barplot shows the number of genetic mutations per gene. Also, it is possible to add annotations to rows or columns. For the columns, an insertion made at the top will remove the barplot. The final result for adding the annotation to the bottom is highlighted in Figure 2.


 
                        1 
                        library
                        (ComplexHeatmap)
 
                        2 
                        library
                        (TCGAbiolinks)
 3
 4 LGGmut 
                        <– 
                        GDCquery
                        _
                        Maf(tumor = 
                        "LGG"
                        , pipelines = 
                        "mutect2"
                        )
 5 GBMmut 
                        <– 
                        GDCquery
                        _
                        Maf(tumor = 
                        "GBM"
                        , pipelines = 
                        "mutect2"
                        )
 
                        6
 
                        7 mut 
                        <– 
                        plyr::
                        rbind
                        .fill(LGGmut,GBMmut)
 8 
 
                        9 
                        # Filtering mutations in gliomas 

                        10 
                        EA
                        _
                        pathways 
                        <– 
                        TCGAbiolinks:::listEA
                        _
                        pathways

                        11 Glioma
                        _
                        pathways 
                        <– 
                        EA
                        _
                        pathways[
                        grep
                        (
                        "glioma"
                        , tolower(EA
                        _
                        pathways
                        $
                        Pathway)),]
12 Glioma
                        _
                        signaling 
                        <– 
                        Glioma
                        _
                        pathways[Glioma
                        _
                        pathways
                        $
                        Pathway == 
                        "Glioma Signaling"
                        ,]
13 Glioma
                        _
                        signaling
                        _
                        genes 
                        <– unlist
                        (
                        strsplit
                        (
                        as
                        .
                        character
                        (Glioma
                        _
                        signaling
                        $
                        Molecules),
                        ","
                        ))
14

                        15 mut 
                        <– 
                        mut[mut
                        $
                        Hugo
                        _
                        Symbol %in% Glioma
                        _
                        signaling
                        _
                        genes,]
16

                        17 samples 
                        <– unique
                        (mut
                        $
                        Tumor
                        _
                        Sample
                        _
                        Barcode)
18 genes 
                        <– unique
                        (mut
                        $
                        Hugo
                        _
                        Symbol)
19 
                        mat <– matrix
                        (0,
                        length
                        (genes),
                        length
                        (samples))
20 
                        colnames
                        (
                        mat
                        ) 
                        <– 
                        samples
21 
                        rownames
                        (
                        mat
                        ) 
                        <– 
                        genes
22
23 pb 
                        <– 
                        txtProgressBar(
                        min 
                        = 0, 
                        max 
                        = 
                        nrow
                        (
                        mat
                        ), style = 3)
24
25 
                        for
                        (i in 1:
                        nrow
                        (
                        mat
                        )) {
26     curGene 
                        <– rownames
                        (
                        mat
                        )[i]
27     setTxtProgressBar(pb, i)
28      
                        for
                        (j in 1:
                        ncol
                        (
                        mat
                        )) {
29         curSample 
                        <– colnames
                        (
                        mat
                        )[j]
30
31          
                        if
                        (
                        length
                        (
                        intersect
                        (mut
                        $
                        Tumor
                        _
                        Sample
                        _
                        Barcode, curSample))==1){
32             mat1 
                        <– 
                        mut[mut
                        $
                        Tumor
                        _
                        Sample
                        _
                        Barcode == curSample,]
33               
                        if
                        (
                        length
                        (
                        intersect
                        (mat1
                        $
                        Hugo
                        _
                        Symbol, curGene))==1){
34                 mat3 
                        <– 
                        mat1[mat1
                        $
                        Hugo
                        _
                        Symbol == curGene,]
35                   
                        mat
                        [curGene,curSample]
                        <– as
                        .
                        character
                        (mat3
                        $
                        Variant
                        _
                        Type)[1]
36             }
37         }
38    }
39 }
40 
                        close
                        (pb)
41
42 
                        mat
                        [
                        mat
                        ==0] 
                        <– 
                        ""

                        43 
                        colnames
                        (
                        mat
                        ) 
                        <– substr
                        (
                        colnames
                        (
                        mat
                        ),1,12)
44
45 
                        mat
                        [
                        is
                        .
                        na
                        (
                        mat
                        )] = 
                        ""

                        46 
                        mat
                        [1:3, 1:3]
47
48 alter
                        _
                        fun = 
                        list
                        (
49     background = 
                        function
                        (x, y, w, h) {
50          
                        grid
                        .
                        rect
                        (x, y, w–unit(0.5, 
                        "mm"
                        ), h–unit(0.5, 
                        "mm"
                        ), gp = gpar(fill = 
                        "#CCCCCC"
                        , 
                        col 
                        = NA))
51     },
52     SNP = 
                        function
                        (x, y, w, h) {
53          
                        grid
                        .
                        rect
                        (x, y, w–unit(0.5, 
                        "mm"
                        ), h–unit(0.5, 
                        "mm"
                        ), gp = gpar(fill = 
                        "blue"
                        , 
                        col 
                        = NA))
54     },
55     DEL = 
                        function
                        (x, y, w, h) {
56          
                        grid
                        .
                        rect
                        (x, y, w-unit(0.5, 
                        "mm"
                        ), h-unit(0.5, 
                        "mm"
                        ), gp = gpar(fill = 
                        "red"
                        , 
                        col 
                        = NA))
57     },
58     INS = 
                        function
                        (x, y, w, h) {
59          
                        grid
                        .
                        rect
                        (x, y, w-unit(0.5, 
                        "mm"
                        ), h
                        ∗
                        0.33, gp = gpar(fill = 
                        "#008000"
                        , 
                        col 
                        = NA))
60     }
61 )
62
63 
                        col 
                        = 
                        c
                        (
                        "INS" 
                        = 
                        "#008000"
                        , 
                        "DEL" 
                        = 
                        "red"
                        , 
                        "SNP" 
                        = 
                        "blue"
                        )
64
65 clin.gbm 
                        <– 
                        GDCquery
                        _
                        clinic(
                        "TCGA–GBM", 
                        "Clinical"
                        )
66 clin.lgg 
                        <– 
                        GDCquery
                        _
                        clinic(
                        "TCGA–LGG"
                        , 
                        "Clinical"
                        )
67 clinical 
                        <– 
                        plyr::
                        rbind
                        .fill(clin.lgg,clin.gbm)
68 annotation 
                        <– 
                        clinical[
                        match
                        (
                        colnames
                        (
                        mat
                        ),clinical
                        $
                        bcr
                        _
                        patient
                        _
                        barcode),

                        69			    
                        c
                        (
                        "disease"
                        ,
                        "vital
                        _
                        status"
                        ,
                        "ethnicity"
                        )]
70 annotation 
                        <– 
                        HeatmapAnnotation(annotation
                        _
                        height = 
                        rep
                        (unit(0.3, 
                        "cm"
                        ),
                        ncol
                        (annotation)),
71	                               
                        df 
                        = annotation,
72      	                       
                        col 
                        = 
                        list
                        (disease = 
                        c(
                        "LGG"
                        =
                        "green"
                        ,

                        73				      
                        "GBM"
                        =
                        "orange"
                        ),
74                                     	      vital
                        _status 
                        = 
                        c
                        (
                        "alive"
                        =
                        "blue"
                        ,
75				     
                        "dead"
                        =
                        "red"
                        ,
76				     
                        "not reported"
                        =
                        "grey"
                        ),
77 	       ethnicity = 
                        c
                        (
                        "hispanic or latino"
                        =
                        "purple"
                        ,

                        78				
                        "not hispanic or latino"
                        =
                        "black"
                        ,

                        79		   
                        "not reported" 
                        = 
                        "grey"
                        )),
80       	                 annotation
                        _legend_
                        param = 
                        list
                        (
                        title_
                        gp = gpar(fontsize = 16,
       fontface = 
                        "bold"
                        ),
81                                                                     
                        labels_
                        gp = gpar(fontsize = 16), 
                        #
        
                        size labels

                        82                                                                     
                        grid_
                        height = unit(8, 
                        "mm"
                        )))
83
84 pdf(
                        "LGG
                        _
                        GBM
                        _
                        oncoprint.pdf"
                        ,width = 20,height = 20)
85 p 
                        <– 
                        oncoPrint(
                        mat
                        , 
                        get
                        _
                        type = 
                        function
                        (x) 
                        strsplit
                        (x, 
                        ";"
                        )[[1]],
86            
                        remove
                        _
                        empty
                        _
                        columns = FALSE,
87           column
                        _order 
                        = NULL, 
                        # Do not sort the columns

                        88           alter
                        _
                        fun = alter
                        _
                        fun, 
                        col 
                        = 
                        col
                        ,
89            
                        row_names_
                        gp = gpar(fontsize = 16),  
                        # set size for row names

                        90           pct
                        _
                        gp = gpar(fontsize = 16), 
                        # set size for percentage labels

                        91            
                        axis_
                        gp = gpar(fontsize = 16),
                        # size of axis

                        92           column
                        _title 
                        = 
                        "OncoPrint for TCGA LGG, genes in Glioma signaling"
                        ,
93           column
                        _title_
                        gp = gpar(fontsize = 22),
94           pct
                        _
                        digits = 2,
95            
                        row_barplot_
                        width = unit(4, 
                        "cm"
                        ), 
                        #size barplot

                        96           bottom
                        _
                        annotation = annotation,
97           heatmap
                        _legend_
                        param = 
                        list
                        (
                        title 
                        = 
                        "Mutations"
                        , at = 
                        c
                        (
                        "DEL"
                        , 
                        "INS"
                        , 
                        "SNP"
                        ),
98                                            
                        labels 
                        = 
                        c
                        (
                        "DEL"
                        , 
                        "INS"
                        , 
                        "SNP"
                        ),
99                                            
                        title_
                        gp = gpar(fontsize = 16, fontface = 
                        "bold"
                        ),
100                                           
                        labels_
                        gp = gpar(fontsize = 16), 
                        # size labels

                        101                                           
                        grid_
                        height = unit(8, 
                        "mm"
                        )
102          )
103 )
104 draw(p, annotation
                        _legend_
                        side = 
                        "bottom"
                        )
105 
                        dev
                        .
                        off
                        ()
                    


**Listing 12. Oncoprint**

**Overview of genomic alterations by circos plot**

**Figure 3.**
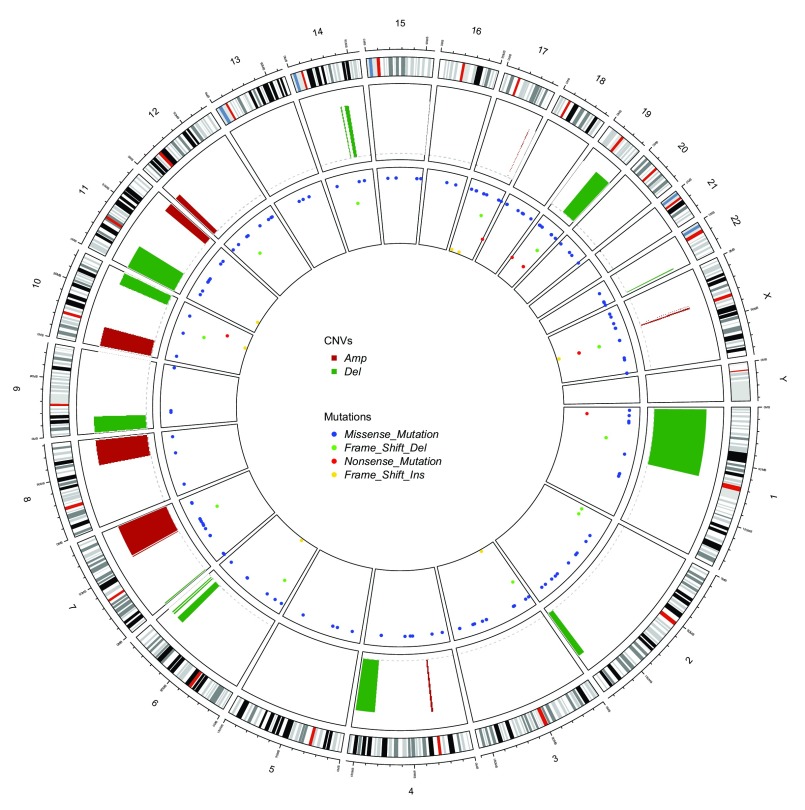
Circos plot of recurrent CNVs and mutations in LGG.

**Figure 4.**
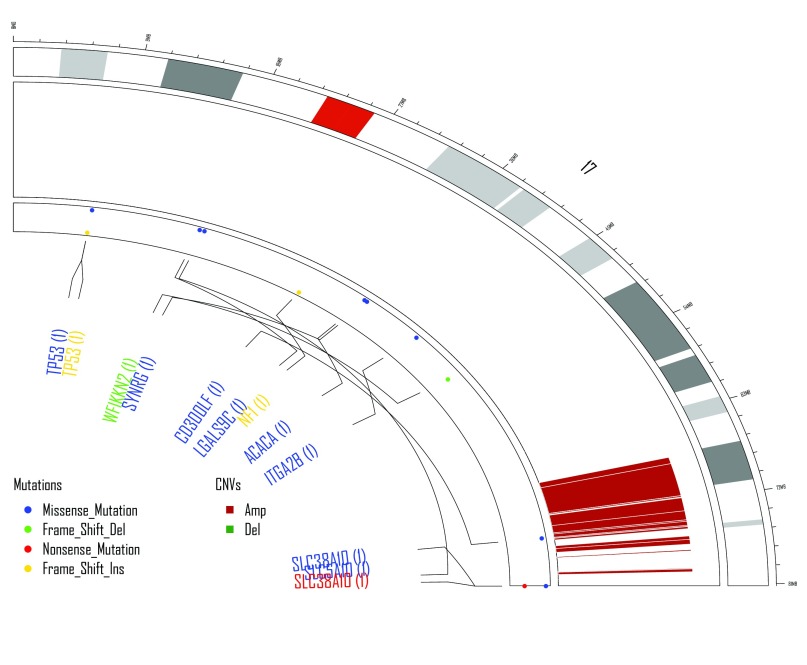
Circos plot of chromosome 17 recurrent CNVs and mutations in LGG.



Genomic alterations in cancer, including CNV and mutations, can be represented in an effective overview plot named circos. We used circlize CRAN package to represent significant CNV (resulting from GAIA analysis) and recurrent mutations (selecting curated genetic variations retrieved from TCGA that are identified in at least two tumor samples) in LGG. Circos plot can illustrate molecular alterations genome-wide or only in one or more selected chromosomes. The Figure 3 shows the resulting circos plot for all chromosomes, while the Figure 4 shows the plot for only the chromosome 17.


  
                        1 
                        ###############################################
  
                        2 
                        ## Genomic aberration overview – Circos plot ##
  
                        3 
                        ###############################################
  
                        4
  
                        5 
                        # Retrieve curated mutations for selected cancer (e.g. "LGG")
  
                        6 
                        library(
                        TCGAbiolinks)
  
                        7 mut 
                        <- 
                        GDCquery
                        _
                        Maf(tumor = 
                        "LGG")
  
                        8 
                        # Select only potentially damaging mutations
  
                        9 mut 
                        <- 
                        mut[mut
                        $
                        Variant
                        _ 
                        Classification %in% 
                        c
                        (
                        "Missense
                        _
                        Mutation",
                        "Nonsense
                        _
                        Mutation",
                        "Nonstop
                        _
          
                        Mutation",
                        "Frame
                        _
                        Shift
                        _
                        Del",
                        "Frame
                        _
                        Shift
                        _
                        Ins"
                        ),]
 10 
                        # Select recurrent mutations (identified in at least two samples)
 
                        11 mut.id 
                        <- 
                        paste0(mut
                        $
                        Chromosome,
                        ":"
                        ,mut
                        $
                        Start
                        _
                        position,
                        "–"
                        ,mut
                        $
                        End
                        _
                        position,
                        "|"
                        ,mut
                        $
                        Reference
                        _
                        Allele)
 12 mut 
                        <- cbind
                        (mut.id, mut)
 13 numSamples 
                        <- table
                        (mut.id)
 14 s.mut 
                        <- names
                        (
                        which
                        (numSamples>=2))
 15 
                        # Prepare selected mutations data for circos plot
 
                        16 s.mut 
                        <- 
                        mut[mut
                        $
                        mut.id %in% s.mut,]
 17 s.mut 
                        <- 
                        s.mut[,
                        c
                        (
                        "Chromosome",
                        "Start
                        _
                        position",
                        "End
                        _
                        position",
                        "Variant
                        _
                        Classification",
                        "Hugo
                        _
                        Symbol"
          
                        )]
 18 s.mut 
                        <- unique
                        (s.mut)
 19 s.mut[,1] 
                        <- as
                        .
                        character
                        (s.mut[,1])
 20 s.mut[,4] 
                        <- as
                        .
                        character
                        (s.mut[,4])
 21 s.mut[,5] 
                        <- as
                        .
                        character
                        (s.mut[,5])
 22 typeNames 
                        <- unique
                        (s.mut[,4])
 23 type 
                        <- c
                        (4:1)
 24 
                        names
                        (type) 
                        <- 
                        typeNames[1:4]
 25 Type 
                        <- 
                        type[s.mut[,4]]
 26 s.mut 
                        <- cbind
                        (s.mut,Type)
 27 s.mut 
                        <- 
                        s.mut[,
                        c
                        (1:3,6,4,5)]
 28
 29 
                        # Load recurrent CNV data for selected cancer (e.g. "LGG")
 
                        30 
                        load
                        (
                        "LGG
                        _
                        CNV
                        _
                        results.rda"
                        )
 31 
                        # Prepare selected sample CNV data for circos plot
 
                        32 s.cnv 
                        <- as
                        .
                        data
                        .
                        frame
                        (RecCNV[RecCNV[,
                        "q—value"
                        ]<=10^—4,
                        c
                        (1:4,6)])
 33 s.cnv 
                        <- 
                        s.cnv[,
                        c
                        (1,3,4,2)]
 34 xidx 
                        <- which
                        (s.cnv
                        $
                        Chromosome==23)
 35 yidx 
                        <- which
                        (s.cnv
                        $
                        Chromosome==24)
 36 
                        s.cnv[xidx,
                        "Chromosome"
                        ] 
                        <- 
                        "X"
 
                        37 
                        s.cnv[yidx,
                        "Chromosome"
                        ] 
                        <- 
                        "Y"
 
                        38 Chromosome 
                        <- sapply
                        (s.cnv[,1],
                        function
                        (x) paste0(
                        "chr"
                        ,x))
 39 s.cnv 
                        <- cbind
                        (Chromosome, s.cnv[,-1])
 40 s.cnv[,1] 
                        <- as
                        .
                        character
                        (s.cnv[,1])
 41 s.cnv[,4] 
                        <- as
                        .
                        character
                        (s.cnv[,4])
 42 s.cnv 
                        <- cbind
                        (s.cnv,CNV=1)
 43 
                        colnames
                        (s.cnv) 
                        <- c
                        (
                        "Chromosome",
                        "Start
                        _
                        position",
                        "End
                        _
                        position",
                        "Aberration
                        _
                        Kind",
                        "CNV"
                        )
 44
 45 
                        # Draw genomic circos plot
 
                        46 
                        library
                        (circlize)
 47 pdf(
                        "CircosPlot.pdf"
                        ,width=15,height=15)
 48 
                        par
                        (mar=
                        c
                        (1,1,1,1), cex=1)
 49 
                        circos.initializeWithIdeogram()
 
                        50 
                        # Add CNV results
 
                        51 
                        colors <- c
                        (
                        "forestgreen",
                        "firebrick"
                        )
 52 
                        names
                        (
                        colors
                        )
                          <- c
                        (0,1)
 53 
                        circos.genomicTrackPlotRegion(s.cnv
                        ,  ylim = 
                        c
                        (0,1.2),
 54                                   
                        panel
                        .fun = 
                        function
                        (region, value, ...) {
 55				      circos.genomicRect(region, value, ytop.column = 2, ybottom = 0,
 56   								
                        col 
                        = 
                        colors
                        [value[[1]]],
 
                        57						         border=
                        "white"
                        )
 58				      cell.xlim = 
                        get
                        .cell.meta.
                        data
                        (
                        "cell.xlim"
                        )
 59				      circos.
                        lines
                        (cell.xlim, 
                        c
                        (0, 0), lty = 2, 
                        col 
                        = 
                        "#00000040"
                        )
 60				  })
 61 
                        # Add mutation results
 
                        62 
                        colors <- c
                        (
                        "blue",
                        "green",
                        "red",
                        "gold"
                        )
 63 
                        names
                        (
                        colors
                        )  
                        <- 
                        typeNames[1:4]
 
                        64
                         circos.genomicTrackPlotRegion(s.mut, ylim = 
                        c
                        (1.2,4.2),
 65				      
                        panel
                        .fun = 
                        function
                        (region, value, ...) {
 66				      circos.genomicPoints(region, value, cex = 0.8, pch = 16, 
                        col 
                        = 
          
                        colors
                        [value[[2]]], ...)
 67				  })
 68
 69 circos.clear()
 70
 71 
                        legend
                        (−0.2, 0.2, bty=
                        "n"
                        , y.intersp=1, 
                        c
                        (
                        "Amp",
                        "Del"
                        ), pch=15, 
                        col
                        =
                        c
                        (
                        "firebrick",
                        "forestgreen"
                        ), 
          
                        title
                        =
                        "CNVs", 
                        text
                        .font=3, cex=1.2, 
                        title
                        .adj=0)
 72 
                        legend
                        (–0.2, 0, bty=
                        "n"
                        , y.intersp=1, 
                        names
                        (
                        colors
                        ), pch=16, 
                        col=
                        colors, 
                        title
                        =
                        "Mutations", 
                        text
                        .font
         =3, cex=1.2, 
                        title
                        .adj=0)
 73 
                        dev
                        .
                        off
                        ()
 
                        74
 75 
                        # Draw single chromosome circos plot (e.g. "Chr 17")
 
                        76 pdf(
                        "CircosPlotChr17.pdf"
                        ,width=18,height=13)
 77 
                        par
                        (mar=
                        c
                        (1,1,1,1),
                        cex=1.5)
 78 circos.
                        par
                        (
                        "start.degree" 
                        = 90, canvas.xlim = 
                        c
                        (0, 1), canvas.ylim = 
                        c
                        (0, 1),
 79            gap.degree = 270, cell.padding = 
                        c
                        (0, 0, 0, 0), track.
                        margin 
                        = 
                        c
                        (0.005, 0.005))
 
                        80 circos.initializeWithIdeogram(chromosome.
                        index 
                        = 
                        "chr17"
                        )
 81 circos.
                        par
                        (cell.padding = 
                        c
                        (0, 0, 0, 0))
 82 
                        # Add CNV results
 
                        83 
                        colors <- c
                        (
                        "forestgreen",
                        "firebrick"
                        )
 84 
                        names
                        (
                        colors
                        )  
                        <- c
                        (0,1)
 
                        85 
                        circos.genomicTrackPlotRegion(s.cnv,  ylim = 
                        c
                        (0,1.2),
 
                        86				      
                        panel
                        .fun = 
                        function
                        (region, value, ...) {
 87                                   circos.genomicRect(region, value, ytop.column = 2, ybottom = 0,
 88							  	
                        col 
                        = 
                        colors
                        [
                        value
                        [[
                        1
                        ]]],
 
                        89						         border=
                        "white"
                        )
 90                                   cell.xlim = 
                        get
                        .cell.meta.
                        data
                        (
                        "cell.xlim"
                        )
 91                                   circos.
                        lines
                        (cell.xlim, 
                        c
                        (0, 0), lty = 2, 
                        col 
                        = 
                        "#00000040"
                        )
 92				  })
 93
 94 
                        # Add mutation results representing single genes
 
                        95 genes.mut 
                        <- 
                        paste0
                        (s.mut
                        $
                        Hugo
                        _
                        Symbol,
                        "–"
                        ,s.mut
                        $
                        Type)
 96 s.mutt 
                        <- cbind
                        (s.mut,genes.mut)
 97 n.mut 
                        <- table
                        (genes.mut)
 98 idx 
                        <- !duplicated
                        (s.mutt
                        $
                        genes.mut)
 99 s.mutt 
                        <- 
                        s.mutt[idx,]
100 s.mutt 
                        <- cbind
                        (s.mutt,num=n.mut[s.mutt
                        $
                        genes.mut])
101 genes.num 
                        <- 
                        paste0(s.mutt
                        $
                        Hugo
                        _
                        Symbol,
                        " ("
                        ,s.mutt
                        $
                        num.Freq,
                        ")"
                        )
102 s.mutt 
                        <- cbind
                        (s.mutt[,–
                        c
                        (6:8)],genes.num)
103 s.mutt[,6] 
                        <- as
                        .
                        character
                        (s.mutt[,6])
104 s.mutt[,4] 
                        <- 
                        s.mutt[,4]
                        /
                        2
105 s.mutt
                        $
                        num.Freq 
                        <- 
                        NULL
106 
                        colors <- c
                        (
                        "blue",
                        "green",
                        "red",
                        "gold"
                        )
107 
                        names
                        (
                        colors
                        )  
                        <- 
                        typeNames[
                        1:4
                        ]

                        108 circos.genomicTrackPlotRegion(s.mutt, ylim = 
                        c
                        (0.3,2.2), track.height = 0.05,
109                                   
                        panel
                        .fun = 
                        function
                        (region, value, ...) {
110                                   circos.genomicPoints(region, value, cex = 0.8, pch = 16, 
                        col 
                        = 
          
                        colors
                        [value[[2]]], ...)
111                               })
112
113 circos.genomicTrackPlotRegion(s.mutt, ylim = 
                        c
                        (0, 1), track.height = 0.1, bg.border = NA)
114 i
                        _
                        track = 
                        get
                        .cell.meta.
                        data
                        (
                        "track.index"
                        )
115
116 circos.genomicTrackPlotRegion(s.mutt, ylim = 
                        c
                        (0,1),
117                                   
                        panel
                        .fun = 
                        function
                        (region, value, ...) {
118                                   circos.genomicText(region, value,
119                                                      y = 1,
120                                                             
                        labels
                        .column = 3,
121                                                             
                        col 
                        = 
                        colors
                        [value[[2]]],
122                                                      facing = 
                        "clockwise"
                        , adj = 
                        c
                        (1, 0.5),
123                                                      posTransform = posTransform.
                        text
                        , cex = 1.5,
         niceFacing = T)
124                               }, track.height = 0.1, bg.border = NA)
125
126 circos.genomicPosTransformLines(s.mutt,
127                                 posTransform = 
                        function
                        (region, value)
128                                     posTransform.
                        text
                        (region,
129
                                                                               y = 1,

                        130                                                              
                        labels 
                        = value[[3]],
131                                                       cex = 0.8, track.
                        index 
                        = i
                        _
                        track+1),
132                                 direction = 
                        "inside"
                        , track.
                        index 
                        = i
                        _
                        track)
133
134 circos.clear()
135
136 
                        legend
                        (0.25, 0.2, 
                        bty=
                        "n"
                        , y.intersp=1, 
                        c
                        (
                        "Amp",
                        "Del"
                        ), pch=15, 
                        col
                        =
                        c
                        (
                        "firebrick",
                        "forestgreen"
                        ),
          
                        title
                        =
                        "CNVs", 
                        text
                        .font=3, cex=1.3, 
                        title
                        .adj=0)
137 
                        legend
                        (0, 0.2, bty=
                        "n"
                        , y.intersp=1, 
                        names
                        (
                        colors
                        ), pch=16, 
                        col
                        =
                        colors, 
                        title
                        =
                        "Mutations", 
                        text
                        .font
         =3, cex=1.3, 
                        title
                        .adj=0)
138 
                        dev.
                        off
                        ()
                    


**Listing 13. Genomic aberration overview by circos plot**

### Transcriptomic analysis

***Pre-Processing Data.*** The LGG and GBM data used for following transcriptomic analysis were downloaded using TCGAbiolinks. We downloaded only primary solid tumor (TP) samples, which resulted in 516 LGG samples and 156 GBM samples, then prepared it in two separate rse objects ( *RangedSummarizedExperiment*) saving them as an R object with a file name including both the name of the cancer and the name of the platform used for gene expression data (see Listing 14).

                         1 
                        library
                        (TCGAbiolinks)
 2
 3 query 
                        <- 
                        GDCquery(project = 
                        "TCGA-GBM"
                        ,

                         4                     
                        data
                        .
                        category 
                        = 
                        "Gene expression"
                        ,
 5                     
                        data
                        .type = 
                        "Gene expression quantification"
                        ,

                         6                   platform = 
                        "Illumina HiSeq"
                        ,

                         7                     
                        file
                        .type = 
                        "results"
                        ,

                         8                     
                        sample
                        .type = 
                        c
                        (
                        "Primary solid Tumor"
                        ),
 9                   legacy = TRUE)
10 GDCdownload(query)
11 gbm.
                        exp <- 
                        GDCprepare(query,
12                          
                        save 
                        = TRUE,
13                       summarizedExperiment = TRUE,
14                          
                        save
                        .filename = 
                        "GBMIllumina
                        _
                        HiSeq.rda"
                        )
15
16 query 
                        <- 
                        GDCquery(project = 
                        "TCGA-LGG"
                        ,
17                     
                        data
                        .
                        category 
                        = 
                        "Gene expression"
                        ,
18                     
                        data
                        .type = 
                        "Gene expression quantification"
                        ,
19                   platform = 
                        "Illumina HiSeq"
                        ,
20                     
                        file
                        .type = 
                        "results"
                        ,
21                     
                        sample
                        .type = 
                        c
                        (
                        "Primary solid Tumor"
                        ),
22                   legacy = TRUE)
23 GDCdownload(query)
24 lgg.
                        exp <- 
                        GDCprepare(query, 
                        save 
                        = TRUE,
25                       summarizedExperiment = TRUE,
26                          
                        save
                        .filename = 
                        "LGGIllumina
                        _
                        HiSeq.rda"
                        )
                    


**Listing 14. Searching, downloading and preparing RNA-seq data with TCGAbiolinks**

To pre-process the data, first, we searched for possible outliers using the *TCGAanalyze_Preprocessing* function, which performs an Array Array Intensity correlation AAIC (lines 14–17 and 26–29 of Listing 15). The Array-array intensity correlation plot (AAIC) is a re-adaptation of the function correlationPlot from the R/Bioconductor affyQCReport package ^31^ that shows an heat map of the array-array Spearman/Pearson rank correlation coefficients. The arrays are ordered using the phenotypic data (if available) in order to place arrays with similar samples adjacent to each other. Self-self correlations are on the diagonal and by definition have a correlation coefficient of 1.0. Data from similar tissues or treatments will tend to have higher coefficients. This plot is useful for detecting outliers, failed hybridizations, or mistracked samples.

In our example, we defined a square symmetric matrix of Pearson correlation among all samples in each cancer type (LGG or GBM). This matrix found 0 samples with low correlation (cor.cut = 0.6) that can be identified as possible outliers.

Second, using the *TCGAanalyze_Normalization* function, which encompasses the functions of the EDASeq package, we normalized mRNA transcripts.

The *TCGAanalyze_Normalization* performs normalization using the following functions from EDASeq: newSeqExpressionSet, withinLaneNormalization, betweenLaneNormalization, counts. The within-lane normalization procedures to adjust for GC-content effect (or other gene-level effects) on read counts ^32^. The between-lane normalization procedures to adjust for distributional differences between lanes (e.g., sequencing depth): global-scaling and full-quantile normalization ^33^.



                         1 
                        library
                        (TCGAbiolinks)
 2
 3 rse 
                        <- get(
                        load
                        (
                        "LGGIllumina
                        _
                        HiSeq.rda"
                        ))

                         4 dataClin
                        _
                        LGG 
                        <- 
                        GDCquery
                        _
                        clinic(
                        "TCGA-LGG"
                        , 
                        "Clinical"
                        )
 5
 6 dataPrep
                        _
                        LGG 
                        <- 
                        TCGAanalyze
                        _
                        Preprocessing(object = rse,
 7                                            
                        cor
                        .
                        cut 
                        = 0.6,
 8                                       datatype = 
                        "raw
                        _
                        count"
                        ,
 9                                       filename = 
                        "LGG
                        _
                        IlluminaHiSeq
                        _
                        RNASeqV2.png"
                        )
10
11 rse 
                        <- get
                        (
                        load
                        (
                        "GBMIllumina
                        _
                        HiSeq.rda"
                        ))
12 dataClin
                        _
                        GBM 
                        <- 
                        GDCquery
                        _
                        clinic(
                        "TCGA-GBM"
                        , 
                        "Clinical"
                        )
13
14 dataPrep
                        _
                        GBM 
                        <- 
                        TCGAanalyze
                        _
                        Preprocessing(object = rse,
15                                                
                        cor
                        .
                        cut 
                        = 0.6,
16                                           datatype = 
                        "raw
                        _
                        count"
                        ,
17                                           filename = 
                        "GBM
                        _
                        IlluminaHiSeq
                        _
                        RNASeqV2.png"
                        )
18
19 dataNorm 
                        <- 
                        TCGAanalyze
                        _
                        Normalization(tabDF = 
                        cbind
                        (dataPrep
                        _
                        LGG, dataPrep
                        _
                        GBM),
20                                       geneInfo = geneInfo,
21                                       method = 
                        "gcContent"
                        )
22
23 dataFilt 
                        <- 
                        TCGAanalyze
                        _
                        Filtering(tabDF = dataNorm,
24                                   method = 
                        "quantile"
                        ,
25                                   qnt.
                        cut 
                        =  0.25)
26
27 
                        save
                        (dataFilt, 
                        file 
                        = paste0(
                        "LGG
                        _
                        GBM
                        _
                        Norm
                        _
                        IlluminaHiSeq.rda"
                        ))
28
29 dataFiltLGG 
                        <- subset
                        (dataFilt, select = 
                        substr
                        (
                        colnames
                        (dataFilt),1,12) %in% dataClin
                        _
                        LGG
                        $
                        bcr
                        _
         
                        patient
                        _
                        barcode)
30 dataFiltGBM 
                        <- subset
                        (dataFilt, select = 
                        substr
                        (
                        colnames
                        (dataFilt),1,12) %in% dataClin
                        _
                        GBM
                        $
                        bcr
                        _
         
                        patient
                        _
                        barcode)
31
32 dataDEGs 
                        <- 
                        TCGAanalyze
                        _
                        DEA(mat1 = dataFiltLGG,
33                             mat2 = dataFiltGBM,
34                             Cond1type = 
                        "LGG"
                        ,
35                             Cond2type = 
                        "GBM"
                        ,
36                             fdr.
                        cut 
                        = 0.01 ,
37                             logFC.
                        cut 
                        = 1,
38                             method = 
                        "glmLRT"
                        )
                    


**Listing 15. Normalizing mRNA transcripts and differential expression analysis with TCGAbiolinks**

Using *TCGAanalyze_DEA*, we identified 2,901 differentially expressed genes (DEG) (log fold change >=1 and FDR < 1%) between 515 LGG and 155 GBM samples.

***EA: enrichment analysis.*** In order to understand the underlying biological process of DEGs we performed an enrichment analysis using *TCGAanalyze_EA_complete* function (see Listing 16).


 
                        1 ansEA 
                        <– 
                        TCGAanalyze
                        _
                        EAcomplete(TFname=
                        "DEA genes LGG Vs GBM"
                        , RegulonList = 
                        rownames
                        (dataDEGs))
 2
 3 TCGAvisualize
                        _
                        EAbarplot(tf = 
                        rownames
                        (ansEA
                        $
                        ResBP),
 4                         GOBPTab = ansEA
                        $
                        ResBP, GOCCTab = ansEA
                        $
                        ResCC,
 5                         GOMFTab = ansEA
                        $
                        ResMF, PathTab = ansEA
                        $
                        ResPat,
 6                         nRGTab = 
                        rownames
                        (dataDEGs),
 7                         nBar = 20)
                    


**Listing 16. Enrichment analysis**

**Figure 5.**
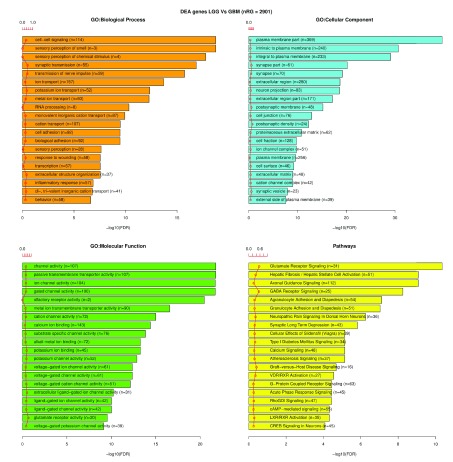
The plot shows canonical pathways significantly over-represented (enriched) by the DEGs (differentially expressed genes) with the number of genes for the main categories of three ontologies (GO:biological process, GO:cellular component, and GO:molecular function, respectively). The most statistically significant canonical pathways identified in DEGs are listed according to their p-value corrected FDR (-Log10) (colored bars) and the ratio of listed genes found in each pathway over the total number of genes in that pathway (ratio, red line).

*TCGAanalyze_EAbarplot* outputs a bar chart as shown in Figure 5 with the number of genes for the main categories of three ontologies (i.e., GO:biological process, GO:cellular component, and GO:molecular function).

The Figure 5 shows canonical pathways significantly over-represented (enriched) by the DEGs. The most statistically significant canonical pathways identified in the DEGs are ranked according to their p-value corrected FDR (-Log10) (colored bars) and the ratio of list genes found in each pathway over the total number of genes in that pathway (ratio, red line).

**Figure 6.**
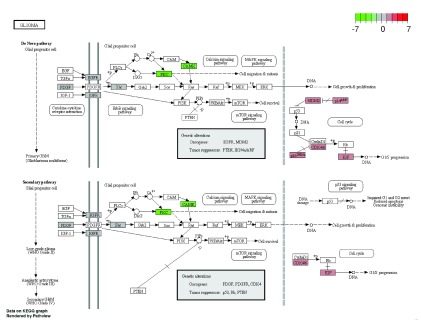
Pathways enrichment analysis : glioma pathway. Red defines genes that are up-regulated and green defines genes that are down-regulated.


 
***PEA: Pathways enrichment analysis.*** To verify if the genes found have a specific role in a pathway, the Bioconductor package pathview ^34^ can be used. Listing 17 shows an example how to use it. It can receive, for example, a named vector of gene with the expression level, the pathway.id which can be found in KEGG database, the species (’hsa’ for Homo sapiens) and the limits for the gene expression (see Figure 6).

                        1 GenelistComplete 
                        <– rownames
                        (assay(rse,1))
 2
 3 
                        # DEGs TopTable
 
                        4 dataDEGsFiltLevel 
                        <– 
                        TCGAanalyze
                        _
                        LevelTab(dataDEGs,
                        "LGG"
                        ,
                        "GBM"
                        ,
 5                                           dataFilt[,
                        colnames
                        (dataFiltLGG)],
 6                                           dataFilt[,
                        colnames
                        (dataFiltGBM)])
 7
 8 dataDEGsFiltLevel
                        $
                        GeneID 
                        <– 
                        0
 9
10 
                        # Converting Gene symbol to geneID

                        11 
                        library
                        (clusterProfiler)
12 eg = 
                        as
                        .
                        data
                        .
                        frame
                         ( bitr(dataDEGsFiltLevel
                        $
                        mRNA,
13                         fromType=
                        "SYMBOL"
                        ,
14                         toType=
                        "ENTREZID"
                        ,
15                         OrgDb=
                        "org.Hs.eg.db"
                        ))
16 eg 
                        <– 
                        eg[
                        !duplicated
                        (eg
                        $
                        SYMBOL),]
17
18 dataDEGsFiltLevel 
                        <– 
                        dataDEGsFiltLevel[dataDEGsFiltLevel
                        $
                        mRNA %in% eg
                        $
                        SYMBOL,]
19
20 dataDEGsFiltLevel 
                        <– 
                        dataDEGsFiltLevel[
                        order
                        (dataDEGsFiltLevel
                        $
                        mRNA,decreasing=FALSE),]
21 eg 
                        <– 
                        eg[
                        order
                        (eg
                        $
                        SYMBOL,decreasing=FALSE),]
22
23 
                        # table(eg
                        $
                        SYMBOL == dataDEGsFiltLevel
                        $
                        mRNA) should be TRUE

                        24 
                        all
                        (eg
                        $
                        SYMBOL == dataDEGsFiltLevel
                        $
                        mRNA)
25 dataDEGsFiltLevel
                        $
                        GeneID 
                        <– 
                        eg
                        $
                        ENTREZID
26
27 dataDEGsFiltLevel
                        _sub <– subset
                        (dataDEGsFiltLevel, select = 
                        c
                        (
                        "GeneID", "logFC"
                        ))
28 genelistDEGs 
                        <– as
                        .
                        numeric
                        (dataDEGsFiltLevel
                        _sub$
                        logFC)
29 
                        names
                        (genelistDEGs) 
                        <– 
                        dataDEGsFiltLevel
                        _sub$
                        GeneID
30
31 
                        require
                        (
                        "pathview"
                        )
32 
                        # pathway.id: hsa05214 is the glioma pathway

                        33 
                        # limit: sets the limit for gene expression legend and color
34 
                        hsa05214 
                        <–  
                        pathview(gene.
                        data  
                        = 
                        genelistDEGs,
35                      pathway.id = 
                        "hsa05214"
                        ,
36                      species    = 
                        "hsa"
                        ,
37                      limit      = 
                        list
                        (gene=
                        as
                        .
                        integer
                        (
                        max
                        (
                        abs
                        (genelistDEGs)))))
                    


**Listing 17. Pathways enrichment analysis with pathview package**

The red genes are up-regulated and the green genes are down-regulated in the LGG samples compared to GBM.

***Inference of gene regulatory networks.*** Starting with the set of differentially expressed genes, we infer gene regulatory networks using the following state-of-the art inference algorithms: ARACNE ^35^, CLR ^36^, MRNET ^37^ and C3NET ^38^. These methods are based on mutual inference and use different heuristics to infer the edges in the network. These methods have been made available via Bioconductor/CRAN packages ( MINET ^39^ and c3net ^38^, respectively).

Many gene regulatory interactions have been experimentally validated and published. These ‘known’ interactions can be accessed using different tools and databases such as BioGrid ^40^ or GeneMANIA ^41^. However, this knowledge is far from complete and in most cases only contains a small subset of the real interactome. The quality assessment of the inferred networks can be carried out by comparing the inferred interactions to those that have been validated. This comparison results in a confusion matrix as presented in Table 2.

**Table 2.** Confusion matrix, comparing inferred network to network of validated interactions.

	validated	not validated/non-existing
inferred	TP	FP
not inferred	FN	TN

Different quality measures can then be computed such as the false positive rate

$$fpr=\frac{FP}{FP+TN},$$

the true positive rate (also called recall)

$$tpr=\frac{TP}{TP+FN}$$

and the precision

$$p=\frac{TP}{TP+FP}.$$

The performance of an algorithm can then be summarized using ROC (false positive rate versus true positive rate) or PR (precision versus recall) curves.

A weakness of this type of comparison is that an edge that is not present in the set of known interactions can either mean that an experimental validation has been tried and did not show any regulatory mechanism or (more likely) has not yet been attempted.

In the following, we ran the nce on i) the 2,901 differentially expressed genes identified in Section “Transcriptomic analysis”.

**Retrieving known interactions**

As previously stated, different sources for protein-protein interactions are available (e.g. I2D, BioGrid database ). In this example, we obtained a set of known interactions from the BioGrid database, but the users can chose their preferred database.



                         1 
                        get
                        .adjacency.biogrid 
                        <– function
                        (tmp.biogrid, 
                        names
                        .genes = NULL){
 2
 3   
                        if
                        (
                        is
                        .
                        null
                        (
                        names
                        .genes)){
 4      
                        names
                        .genes 
                        <– sort
                        (
                        union
                        (
                        unique
                        (tmp.biogrid[,
                        "Official.Symbol.Interactor.A"
                        ]),
 5      
                        unique
                        (tmp.biogrid[,
                        "Official.Symbol.Interactor.B"
                        ])))
 6     ind 
                        <– seq
                        (1,
                        nrow
                        (tmp.biogrid))
 7   }
                        else
                        {
 8     ind.A 
                        <– which
                        (tmp.biogrid[,
                        "Official.Symbol.Interactor.A"
                        ]%in%
                        names
                        .genes)
 9     ind.B 
                        <– which
                        (tmp.biogrid[,
                        "Official.Symbol.Interactor.B"
                        ]%in%
                        names
                        .genes)
10
11     ind 
                        <– intersect
                        (ind.A, ind.B)
12   }
13
14    
                        mat
                        .biogrid 
                        <– matrix
                        (0, 
                        nrow
                        =
                        length
                        (
                        names
                        .genes), 
                        ncol
                        =
                        length
                        (
                        names
                        .genes), 
                        dimnames
                        =
                        list
                        (
                        names
                        .
         genes, 
                        names
                        .genes))
15
16    
                        for
                        (i in ind){
17       
                        mat
                        .biogrid[tmp.biogrid[i,
                        "Official.Symbol.Interactor.A"
                        ], tmp.biogrid[i,
                        "Official.Symbol.
         Interactor.B"
                        ]] 
                        <– mat
                        .biogrid[tmp.biogrid[i,
                        "Official.Symbol.Interactor.B"
                        ], tmp.biogrid[i,
                        "
         Official.Symbol.Interactor.A"
                        ]] 
                        <– 
                        1
18   }
19    
                        diag
                        (
                        mat
                        .biogrid) 
                        <– 
                        0
20
21    
                        return
                        (
                        mat
                        .biogrid)
22 }
                    


There are 3,941 unique interactions between the 2,901 differentially expressed genes.

**Using differentially expressed genes from TCGAbiolinks workflow**

We start this analysis by inferring two gene regulatory networks (the corresponding number of edges are presented in Table 3) for the GBM data set and one gene set for the LGG data.

**Table 3.** Number of edges in the inferred gene regulatory networks; first two lines: networks inferred using 2,901 differentially expressed genes.

gene set	inference algorithm	aracne	c3net	clr	mrnet
DE	GBM	5,903	2,678	1,718,328	1,682,334
	LGG	4,443	2,684	1,939,142	1,859,121


 
                        1 
                        ### plot details (colors 
                        & 
                        symbols)
 
                        2 mycols
                        <–c
                        (
                        '#e41a1c'
                        ,
                        '#377eb8'
                        ,
                        '#4daf4a'
                        ,
                        '#984ea3'
                        ,
                        '#ff7f00'
                        ,
                        '#ffff33'
                        ,
                        '#a65628'
                        )
 
                        3
 
                        4 
                        ### load network inference libraries
 
                        5 
                        library
                        (minet)
 
                        6 
                        library
                        (c3net)
 
                        7
 
                        8 
                        ### deferentially identified genes using TCGAbiolinks
 
                        9 
                        names
                        .genes.
                        de <– rownames
                        (dataDEGs)
10
11 
                        ### read biogrid info

                        12 
                        library
                        (downloader)
13 
                        file <– 
                        "http:
                        //
                        thebiogrid.org
                        /
                        downloads
                        /
                        archives
                        /
                        Release%20Archive
                        /
                        BIOGRID–3.4.133
                        /
                        BIOGRID–ALL
        –3.4.133.tab2.zip"

                        14 
                        download
                        (
                        file
                        ,
                        basename
                        (
                        file
                        ))
15 unzip(
                        basename
                        (
                        file
                        ),junkpaths =T)
16 tmp.biogrid 
                        <– read
                        .
                        csv
                        (
                        gsub
                        (
                        "zip"
                        ,
                        "txt"
                        ,
                        basename
                        (
                        file
                        )), header=TRUE, sep=
                        "\t"
                        , stringsAsFactors=
        FALSE)
17 
18 net.biogrid.
                        de <– get
                        .adjacency.biogrid(tmp.biogrid, 
                        names
                        .genes.
                        de
                        )
19
20 
                        for
                        (cancertype in 
                        c
                        (
                        "LGG"
                        , 
                        "GBM"
                        )) {
21
22      
                        if
                        (cancertype == 
                        "GBM"
                        ){
23         mydata 
                        <– 
                        dataFiltGBM[
                        names
                        .genes.
                        de
                        , ]
24     }
                        else if
                        (cancertype == 
                        "LGG"
                        ){
25         mydata 
                        <– 
                        dataFiltLGG[
                        names
                        .genes.
                        de
                        , ]
26     }
27      
                        ### infer networks

                        28     net.aracne 
                        <– 
                        minet(
                        t
                        (mydata), method = 
                        "aracne"
                        )
29     net.mrnet 
                        <– 
                        minet(
                        t
                        (mydata))
30     net.clr 
                        <– 
                        minet(
                        t
                        (mydata), method = 
                        "clr"
                        )
31     net.c3net 
                        <– 
                        c3net(mydata)
32
33      
                        ### validate compared to biogrid network

                        34     tmp.val 
                        <– list
                        (validate(net.aracne, net.biogrid.
                        de
                        ), validate(net.mrnet, net.biogrid.
                        de
                        ),
35                     validate(net.clr, net.biogrid.
                        de
                        ), validate(net.c3net, net.biogrid.
                        de
                        ))
36
37      
                        ### plot roc and compute auc for the different networks

                        38     dev1 
                        <– show
                        .roc(tmp.val[[1]],cex=0.3,
                        col
                        =mycols[1],type=
                        "l"
                        )
39     res.auc 
                        <– 
                        auc.roc(tmp.val[[1]])
40	
                        for
                        (
                        count 
                        in 2:
                        length
                        (tmp.val)){
41          
                        show
                        .roc(tmp.val[[
                        count
                        ]],device=dev1,cex=0.3,
                        col
                        =mycols[
                        count
                        ],type=
                        "l"
                        )
42         res.auc 
                        <– c
                        (res.auc, auc.roc(tmp.val[[
                        count
                        ]]))
43     }
44
45      
                        legend
                        (
                        "bottomright"
                        , 
                        legend
                        =
                        paste
                        (
                        c
                        (
                        "aracne"
                        ,
                        "mrnet"
                        ,
                        "clr"
                        ,
                        "c3net"
                        ), 
                        signif
                        (res.auc,4), sep=
                        ": "
                        ),
46              
                        col
                        =mycols[1:
                        length
                        (tmp.val)],lty=1, bty=
                        "n" 
                        )
47      
                        dev
                        .copy2pdf(width=8,height=8,device = dev1, 
                        file 
                        = paste0(
                        "roc
                        _
                        biogrid
                        _
                        "
                        ,cancertype,
                        ".pdf"
                        ))
48      
                        save
                        (net.aracne, net.mrnet, net.clr, net.c3net, 
                        file
                        =paste0(
                        "nets
                        _
                        "
                        ,cancertype,
                        ".RData"
                        ))
49
50 }
                    


**Figure 7.**
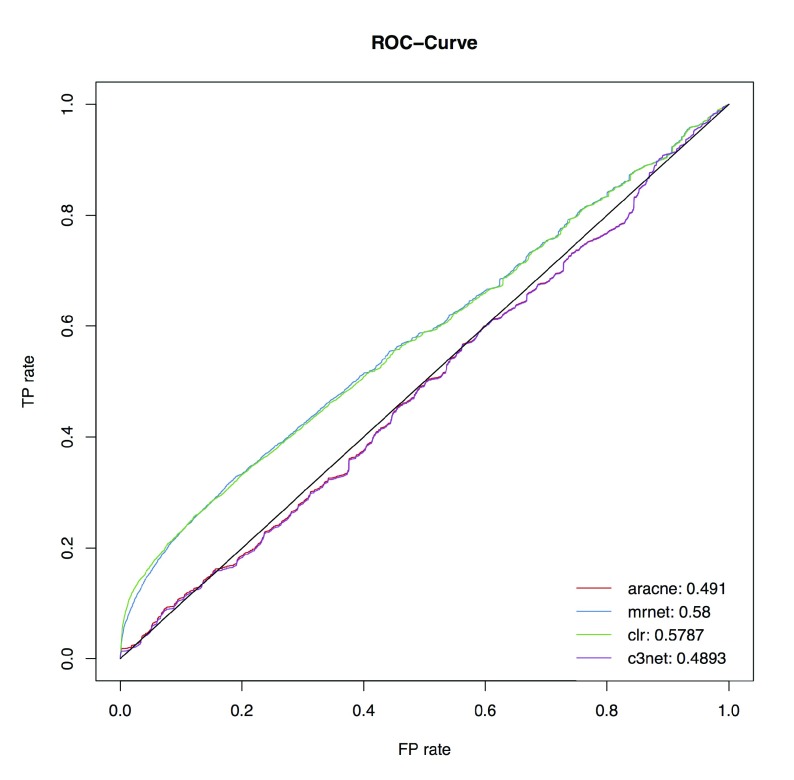
ROC with corresponding AUC for inferred GBM networks compared to BioGrid interactions using 2901 genes.

In Figure 7, the obtained ROC curve and the corresponding area under curve (AUC) are presented. It can be observed that CLR and MRNET perform best when comparing the inferred network with known interactions from the BioGrid database.

### Epigenetic analysis

The DNA methylation is an important component in numerous cellular processes, such as embryonic development, genomic imprinting, X-chromosome inactivation, and preservation of chromosome stability ^42^.

In mammals DNA methylation is found sparsely but globally, distributed in definite CpG sequences throughout the entire genome; however, there is an exception. CpG islands (CGIs) which are short interspersed DNA sequences that are enriched for GC. These islands are normally found in sites of transcription initiation and their methylation can lead to gene silencing ^43^.

Thus, the investigation of the DNA methylation is crucial to understanding regulatory gene networks in cancer as the DNA methylation represses transcription ^44^. Therefore, the DMR (Differentially Methylation Region) detection can help us investigate regulatory gene networks.

This section describes the analysis of DNA methylation using the Bioconductor package TCGAbiolinks ^8^. For this analysis, and due to the time required to perform it, we selected only 10 LGG samples and 10 GBM samples that have both DNA methylation data from Infinium HumanMethylation450 and gene expression from Illumina HiSeq 2000 RNA Sequencing Version 2 analysis (lines 1–56 of the Listing 18 describes how to make the data acquisition). We started by checking the mean DNA methylation of different groups of samples, then performed a DMR in which we search for regions of possible biological significance, (e.g., regions that are methylated in one group and unmethylated in the other). After finding these regions, they can be visualized using heatmaps.

***Visualizing the mean DNA methylation of each patient.*** It should be highlighted that some pre-processing of the DNA methylation data was done. The DNA methylation data from the 450k platform has three types of probes cg (CpG loci), ch (non-CpG loci) and rs (SNP assay). The last type of probe can be used for sample identification and tracking and should be excluded for differential methylation analysis according to the ilumina manual. Therefore, the rs probes were removed (see Listing 18 lines 68). Also, probes in chromosomes X, Y were removed to eliminate potential artifacts originating from the presence of a different proportion of males and females ^45^. The last pre-processing steps were to remove probes with at least one NA value (see Listing 18 lines 65).

After this pre-processing step and using the function *TCGAvisualize_meanMethylation* function, we can look at the mean DNA methylation of each patient in each group. It receives as argument a *summarizedExperiment* object with the DNA methylation data, and the arguments *groupCol* and *subgroupCol* which should be two columns from the sample information matrix of the *summarizedExperiment* object (accessed by the *colData* function) (see Listing 18 lines 70–74).


 
                        1 
                        #––––––––––––––––––––––––––––
 
                        2 
                        # Obtaining DNA methylation
 
                        3 
                        #––––––––––––––––––––––––––––
 
                        4 
                        library
                        (TCGAbiolinks)
 
                        5 
                        library
                        (stringr)
 
                        6 
                        # Samples
 
                        7 matched
                        _
                        met
                        _exp <– function
                        (project, n = NULL){
 
                        8      
                        # get primary solid tumor samples: DNA methylation
 
                        9     message(
                        "Download DNA methylation information"
                        )
10     met450k 
                        <- 
                        GDCquery(project = project,
11 			      
                        data
                        .
                        category 
                        = 
                        "DNA methylation"
                        ,
12			   platform = 
                        "Illumina Human Methylation 450"
                        ,
13 			   legacy = TRUE,
14 			      
                        sample
                        .type = 
                        c
                        (
                        "Primary solid Tumor"
                        ))
15     met450k.tp 
                        <-  
                        met450k
                        $
                        results[[1]]
                        $
                        cases
16
17     
                        # get primary solid tumor samples: RNAseq

                        18     message(
                        "Download gene expression information"
                        )
19     
                        exp <- 
                        GDCquery(project = project,
20 			 
                        data
                        .
                        category 
                        = 
                        "Gene expression"
                        ,
21 			 
                        data
                        .type = 
                        "Gene expression quantification"
                        ,
22                     platform = 
                        "Illumina HiSeq"
                        ,
23 			 
                        file
                        .type =  
                        "results"
                        ,
24 			 
                        sample
                        .type = 
                        c
                        (
                        "Primary solid Tumor"
                        ),
25 	               legacy = TRUE)
26     
                        exp
                        .tp 
                        <-  exp$
                        results[[1]]
                        $
                        cases
27     
                        print
                        (
                        exp
                        .tp[1:10])
28     
                        # Get patients with samples in both platforms

                        29     patients 
                        <– unique
                        (
                        substr
                        (
                        exp
                        .tp,1,15)[
                        substr
                        (
                        exp
                        .tp,1,12) %in% 
                        substr
                        (met450k.tp,1,12)])
30     
                        if
                        (
                        !is
                        .
                        null
                        (n)) patients 
                        <– 
                        patients[1:n] 
                        # get only n samples

                        31     
                        return
                        (patients)
32 }
33 lgg.samples 
                        <– 
                        matched
                        _
                        met
                        _exp
                        (
                        "TCGA–LGG"
                        , n = 10)
34 gbm.samples 
                        <– 
                        matched
                        _
                        met
                        _exp
                        (
                        "TCGA–GBM"
                        , n = 10)
35 samples 
                        <– c
                        (lgg.samples,gbm.samples)
36
37
38 
                        #–––––––––––––––––––––––––––––––––––

                        39 
                        # 1 – Methylation

                        40 
                        # ––––––––––––––––––––––––––––––––––

                        41 
                        # For methylation it is quicker in this case to download the tar.gz file

                        42 
                        # and get the samples we want instead of downloading files by files

                        43 query.lgg 
                        <- 
                        GDCquery(project = 
                        "TCGA-LGG"
                        ,
44		            
                        data
                        .
                        category 
                        = 
                        "DNA methylation"
                        ,
45		         platform = 
                        "Illumina Human Methylation 450"
                        ,
46 			 legacy = TRUE, barcode = lgg.samples)
47 GDCdownload(query.lgg)
48 met.lgg 
                        <-
                        GDCprepare(query.lgg, 
                        save 
                        = FALSE)
49
50 query.gbm 
                        <- 
                        GDCquery(project = 
                        "TCGA-GBM"
                        ,
51 			    
                        data
                        .
                        category 
                        = 
                        "DNA methylation"
                        ,
52			 platform = 
                        "Illumina Human Methylation 450"
                        ,
53 			 legacy = TRUE, barcode = gbm.samples)
54 GDCdownload(query.gbm)
55 met.gbm 
                        <- 
                        GDCprepare(query.gbm, 
                        save 
                        = FALSE)
56 met 
                        <- 
                        SummarizedExperiment::
                        cbind
                        (met.lgg, met.gbm)
57
58 
                        #––––––––––––––––––––––––––––

                        59 
                        # Mean methylation

                        60 
                        #––––––––––––––––––––––––––––

                        61 
                        # Plot a barplot for the groups in the disease column in the

                        62 
                        # summarizedExperiment object

                        63
64 
                        # remove probes with NA (similar to na.omit)

                        65 met 
                        <– subset
                        (met,
                        subset 
                        = (rowSums(
                        is
                        .
                        na
                        (assay(met))) == 0))
66
67 
                        # remove probes in chromossomes X, Y and NA

                        68 met 
                        <– subset
                        (met,
                        subset 
                        = 
                        !as
                        .
                        character
                        (seqnames(met)) %in% 
                        c
                        (
                        "chrNA"
                        ,
                        "chrX"
                        ,
                        "chrY"
                        ))
69
70 TCGAvisualize
                        _
                        meanMethylation(met,
71                               groupCol = 
                        "disease
                        _
                        type"
                        ,
72                               group.
                        legend  
                        = 
                        "Groups"
                        ,
73                               filename = 
                        "mean
                        _
                        lgg
                        _
                        gbm.png"
                        ,
74                                   
                        print
                        .pvalue = TRUE)
                    


**Listing 18. Visualizing the DNA mean methylation of groups**

**Figure 8.**
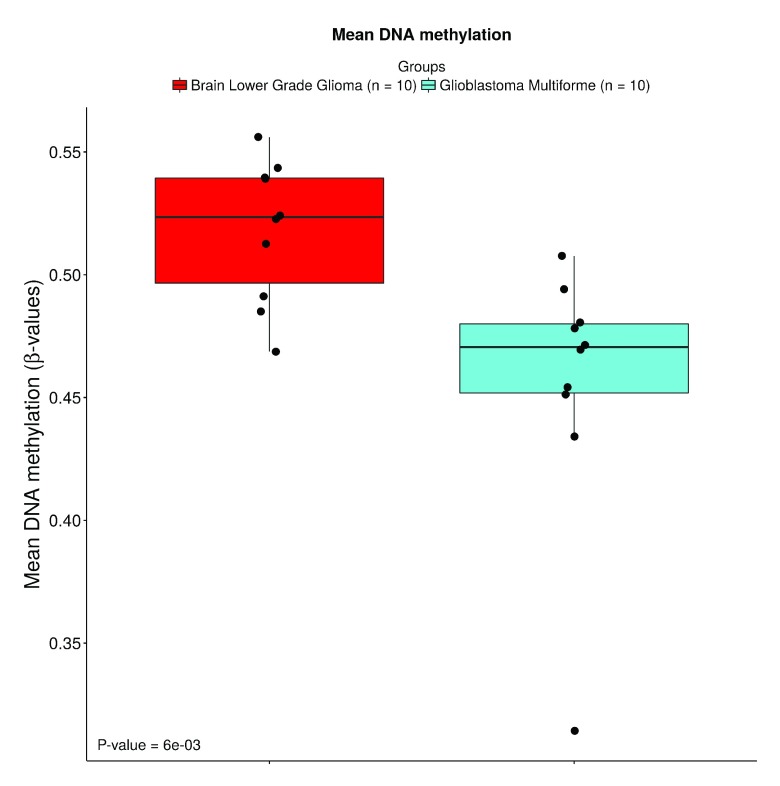
Boxplot of mean DNA methylation of each sample (black dots).

Figure 8 illustrates a mean DNA methylation plot for each sample in the GBM group (140 samples) and a mean DNA methylation for each sample in the LGG group. Genome-wide view of the data highlights a difference between the groups of tumors (p-value = 6.1x10 ^−06^).

***Searching for differentially methylated CpG sites.*** The next step is to define differentially methylated CpG sites between the two groups. This can be done using the *TCGAanalyze_DMR* function (see Listing 19). The DNA methylation data (level 3) is presented in the form of beta-values that uses a scale ranging from 0.0 (probes completely unmethylated) up to 1.0 (probes completely methylated).

To find these differentially methylated CpG sites, first, the function calculates the difference between the mean DNA methylation (mean of the beta-values) of each group for each probe. Second, it test for differential expression between two groups using the Wilcoxon test adjusting by the Benjamini-Hochberg method. Arguments of TCGAanalyze_DMR was set to require a minimum absolute beta-values difference of 0.25 and an adjusted p-value of less than 10 ^−2^.

**Figure 9.**
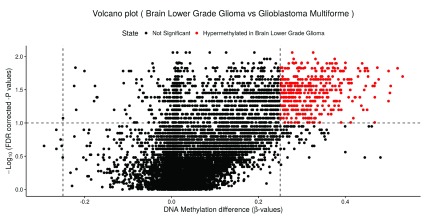
Volcano plot: searching for differentially methylated CpG sites (x-axis:difference of mean DNA methylation, y-axis: statistical significance).

After these tests, a volcano plot (x-axis: difference of mean DNA methylation, y-axis: statistical significance) is created to help users identify the differentially methylated CpG sites and return the object with the results in the rowRanges. Figure 9 shows the volcano plot produced by Listing 19. This plot aids the user in selecting relevant thresholds, as we search for candidate biological DMRs.


 
                        1 
                        # Becareful
                        ! 
                        Depending on the number of probes and samples this function might take some days.
 
                        2 
                        # To make this example faster we used only the chromosome 9
 
                        3 
                        # This should take some minutes
 
                        4 met.chr9 
                        <– subset
                        (met,
                        subset 
                        = 
                        as
                        .
                        character
                        (seqnames(met)) %in% 
                        c
                        (
                        "chr9"
                        ))
 
                        5
 
                        6 met.chr9 
                        <– 
                        TCGAanalyze
                        _
                        DMR(met.chr9,
 
                        7                             groupCol = 
                        "disease
                        _
                        type"
                        , 
                        # a column in the colData matrix
 
                        8                             group1 = 
                        "Glioblastoma Multiforme"
                        , 
                        # a type of the disease type column
 
                        9                             group2= 
                        "Brain Lower Grade Glioma"
                        , 
                        # a type of the disease column

                        10                             p.
                        cut 
                        = 10^–2,
11                             diffmean.
                        cut 
                        = 0.25,
12                                
                        legend 
                        = 
                        "State"
                        ,
13                                
                        plot
                        .filename = 
                        "LGG
                        _
                        GBM
                        _
                        metvolcano.png"
                        ,
14                             cores = 1 
                        # if set to 1 there will be a progress bar

                        15 )
                    


**Listing 19. Finding differentially methylated CpG sites**

**Figure 10.**
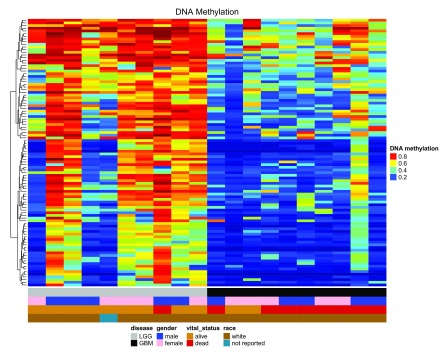
Heatmap of DNA methylation in probes. Rows are probes and columns are samples (patients). The DNA methylation values range from 0.0 (completely DNA unmethylated, blue) to 1.0 (completely DNA methylated, red). The groups of each sample were annotated in the top bar and the DNA methylation status for each probe was annotated in the right bar.

To visualize the level of DNA methylation of these probes across all samples, we use heatmaps that can be generate by the bioconductor package complexHeatmap ^30^. To create a heatmap using the complexHeatmap package, the user should provide at least one matrix with the DNA methylation levels. Also, annotation layers can be added and placed at the bottom, top, left side and right side of the heatmap to provide additional metadata description. The Listing 20 shows the code to produce the heatmap of a DNA methylation datum ( Figure 10).


 
                        1 
                        #––––––––––––––––––––––––––
 
                        2 
                        # DNA methylation heatmap
 
                        3 
                        #–––––––––––––––––––––––––
 
                        4 
                        library
                        (ComplexHeatmap)
 
                        5
 
                        6 clin.gbm 
                        <– 
                        GDCquery
                        _
                        clinic(
                        "TCGA-GBM"
                        , 
                        "Clinical"
                        )
 
                        7 clin.lgg 
                        <– 
                        GDCquery
                        _
                        clinic(
                        "TCGA-LGG"
                        , 
                        "Clinical"
                        )
 
                        8 clinical 
                        <– 
                        plyr::
                        rbind
                        .fill(clin.lgg, clin.gbm)
 
                        9

                        10 
                        # get the probes that are Hypermethylated or Hypomethylated

                        11 
                        # met is the same object of the section 'DNA methylation analysis'

                        12 sig.met 
                        <– 
                        met.chr9[values(met.chr9)[,
                        "status.Glioblastoma.Multiforme.Brain.Lower.Grade.Glioma"
                        ] %in%
	  
                        c
                        (
                        "Hypermethylated"
                        ,
                        "Hypomethylated"
                        ),]
13
14 
                        # To speed up the example, we will select no more than 100 probes

                        15 nb.probes 
                        <- ifelse
                        (
                        nrow
                        (sig.met) > 100, 100, 
                        nrow
                        (sig.met)) 
                        # If there is more than 100 get 100

                        16 sig.met.100 
                        <– 
                        sig.met[1:nb.probes,]
17
18 
                        # top annotation, which sampples are LGG and GBM

                        19 
                        # We will add clinical data as annotation of the samples

                        20 
                        # we will sort the clinical data to have the same order of the DNA methylation matrix

                        21 clinical.
                        order <– 
                        clinical[
                        match
                        (
                        substr
                        (
                        colnames
                        (sig.met.100),1,12),clinical
                        $
                        bcr
                        _
                        patient
                        _
                        barcode),]
22 ta = HeatmapAnnotation(
                        df 
                        = clinical.
                        order
                        [,
                        c
                        (
                        "disease"
                        ,
                        "gender"
                        ,
                        "vital
                        _
                        status"
                        ,
                        "race"
                        )],
23                           
                        col 
                        = 
                        list
                        (disease = 
                        c
                        (
                        "LGG" 
                        = 
                        "grey"
                        , 
                        "GBM" 
                        = 
                        "black"
                        ),
24                                   gender = 
                        c
                        (
                        "male"
                        =
                        "blue"
                        ,
                        "female"
                        =
                        "pink"
                        )))
25
26 
                        # row annotation: add the status for LGG in relation to GBM

                        27 
                        # For exmaple: status.gbm.lgg Hypomethyated means that the

                        28 
                        # mean DNA methylation of probes for lgg are hypomethylated

                        29 
                        # compared to GBM ones.

                        30 ra = rowAnnotation(
                        df 
                        = values(sig.met.100)[
                        "status.Glioblastoma.Multiforme.Brain.Lower.Grade.Glioma"
                        ],
31                      
                        col 
                        = 
                        list
                        (
                        "status.Glioblastoma.Multiforme.Brain.Lower.Grade.Glioma" 
                        = 
         
                        c
                        (
                        "Hypomethylated" 
                        = 
                        "orange"
                        ,
32                     					  
                        "Hypermethylated" 
                        = 
                        "darkgreen"
                        )),
33                    width = unit(1, 
                        "cm"
                        ))
34
35 heatmap 
                        <– 
                        Heatmap(assay(sig.met.100),
36                    name = 
                        "DNA methylation"
                        ,
37                       
                        col 
                        = matlab::jet.
                        colors
                        (200),
38                       
                        show_row_names 
                        = F,
39                    cluster
                        _
                        rows = T,
40                    cluster
                        _
                        columns = F,
41			 
                        show_
                        column
                        _names 
                        = F,
42                    bottom
                        _
                        annotation = ta,
43                    column
                        _ title 
                        = 
                        "DNA methylation"
                        ) 
44 
                        # Save to pdf

                        45 pdf(
                        "heatmap.pdf"
                        ,width = 10, height = 8)
46 draw(heatmap, annotation
                        _legend_
                        side =  
                        "bottom"
                        )
47 
                        dev
                        .
                        off
                        ()
                    


**Listing 20. Creating heatmaps for DNA methylation using ComplexHeatmap**

***Motif analysis.*** Motif discovery is the attempt to extract small sequence signals hidden within largely non-functional intergenic sequences. These small sequence nucleotide signals (6–15 bp) might have a biological significance as they can be used to control the expression of genes. These sequences are called Regulatory motifs. The bioconductor package rGADEM ^46, 47^ provides an efficient *de novo* motif discovery algorithm for large-scale genomic sequence data.

The user may be interested in looking for unique signatures in the regions defined by ‘differentially methylated’ to identify candidate transcription factors that could bind to these elements affected by the accumulation or absence of DNA methylation. For this analysis we use a sequence of 100 bases before and after the probe location (See lines 6–8 in the Listing 21). An object will be returned which contains all relevant information about your motif analysis (i.e., sequence consensus, pwm, chromosome, p-value, etc).

**Figure 11.**
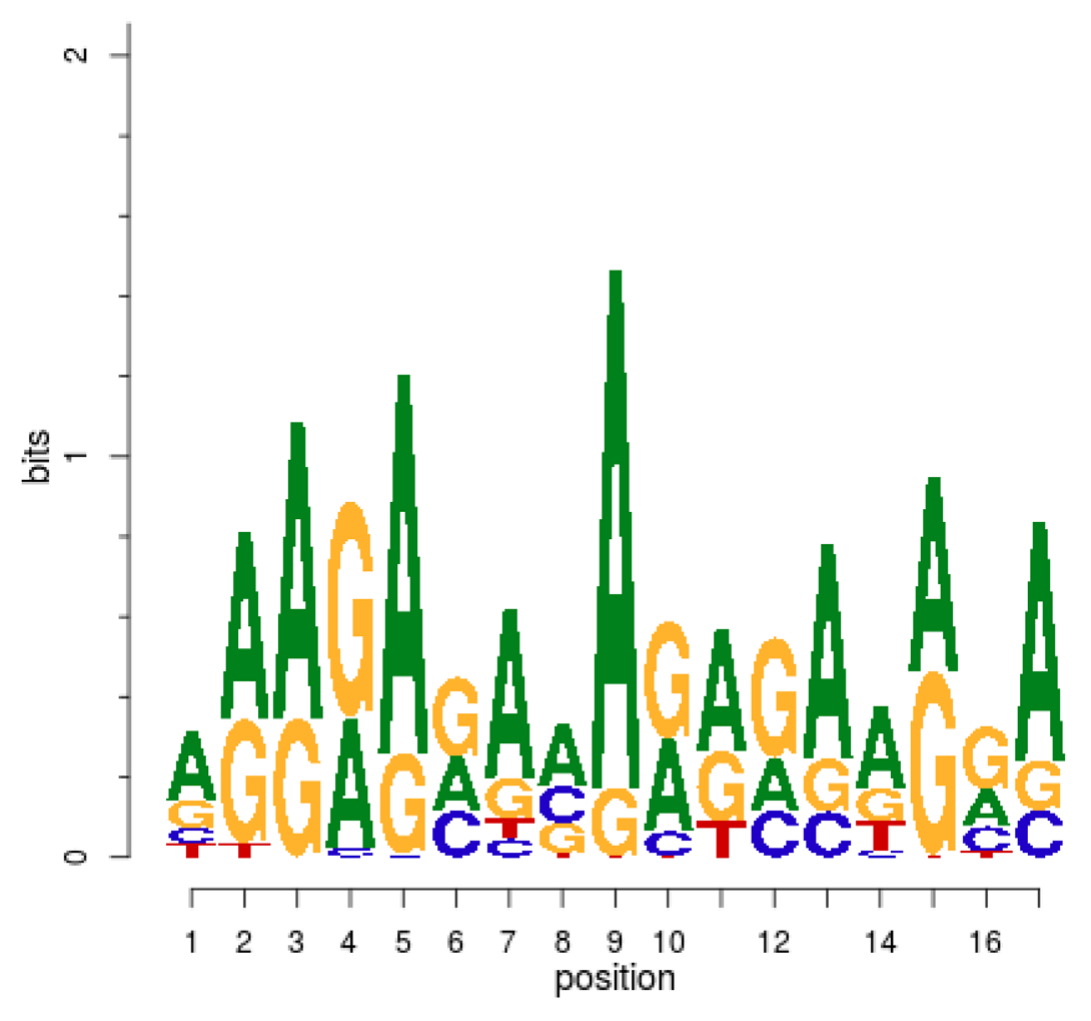
Motif logos found during *de-novo* motif analysis.

Using bioconductor package motifStack ^48^ it is possible to generate a graphic representation of multiple motifs with different similarity scores (see Figure 11).


 
                        1 
                        library
                        (rGADEM)
 
                        2 
                        library
                        (BSgenome.Hsapiens.UCSC.hg19)
 
                        3 
                        library
                        (motifStack)
 
                        4
 
                        5 probes 
                        <– 
                        rowRanges(met.chr9)[values(met.chr9)[,
                        "status.Glioblastoma.Multiforme.Brain.Lower.Grade.
        Glioma"
                        ]%in% 
                        c
                        (
                        "Hypermethylated"
                        ,
                        "Hypomethylated"
                        ),]
 
                        6 
                        # Get hypo
                        /
                        hyper methylated probes and make a 200bp window
 
                        7 
                        # surrounding each probe.
 
                        8 
                        sequence <– 
                        RangedData(space=
                        as
                        .
                        character
                        (probes@seqnames),
 
                        9                        IRanges(
                        start
                        =probes@ranges@start – 100,
10                                   
                        end
                        =probes@ranges@start + 100), strand=
                        "
                        ∗
                        "
                        )
11 
                        #look for motifs

                        12 gadem 
                        <– 
                        GADEM(
                        sequence
                        , verbose=FALSE, genome=Hsapiens)
13
14 
                        # How many motifs were found?

                        15 
                        length
                        (gadem@motifList)
16
17 
                        # get the number of occurences

                        18 nOccurrences(gadem)
19
20 
                        # view all sequences consensus

                        21 consensus(gadem)
22
23 
                        # print motif

                        24 pwm 
                        <– 
                        getPWM(gadem)
25 pfm  
                        <– new
                        (
                        "pfm"
                        ,
                        mat
                        =pwm[[1]],name=
                        "Novel Site 1"
                        )
26 plotMotifLogo(pfm)
27
28 
                        # Number of instances of motif 1?

                        29 
                        length
                        (gadem@motifList[[1]]@alignList)
                    


**Listing 21. rGADEM: *de novo* motif discovery**

**Figure 12.**
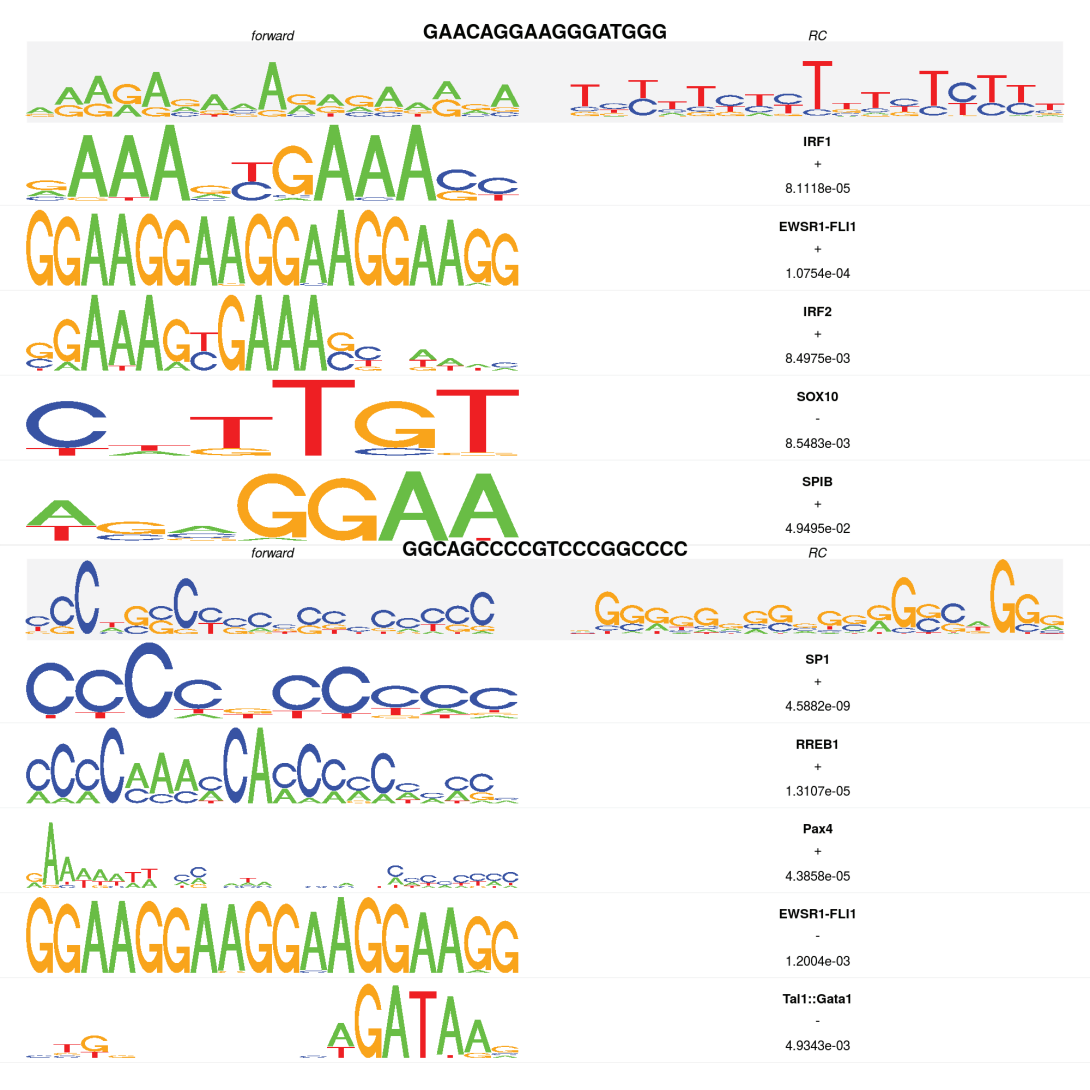
Identified transcription factors: the sequence logo, the name of the motif match and the p-value of the alignment.

After rGADEM returns it’s results, the user can use MotIV package ^49– 52^ to start the motif matching analysis (line 4 of Listing 22). The result is shown in Figure 12.


 
                        1 
                        library
                        (MotIV)
 
                        2
 
                        3 analysis.jaspar 
                        <– 
                        motifMatch(pwm)
 
                        4 
                        summary
                        (analysis.jaspar)
 
                        5 
                        plot
                        (analysis.jaspar, 
                        ncol
                        =1, top=5, 
                        rev
                        =FALSE,
 
                        6      main=
                        ""
                        , bysim=TRUE, cex=0.3)
 
                        7
 
                        8 
                        # visualize the quality of the results around the alignments
 
                        9 
                        # E–value give an estimation of the match.

                        10 alignment 
                        <– 
                        viewAlignments(analysis.jaspar )
11 
                        print
                        (alignment[[1]])
                    


**Listing 22. MotIV: motifs matches analysis.**

### Integrative (Epigenomic & Transcriptomic) analysis

Recent studies have shown that providing a deep integrative analysis can aid researchers in identifying and extracting biological insight from high through put data ^42, 53, 54^. In this section, we will introduce a Bioconductor package called ELMER to identify regulatory enhancers using gene expression + DNA methylation data + motif analysis. In addition, we show how to integrate the results from the previous sections with important epigenomic data derived from both the ENCODE and Roadmap.

**Figure 13.**
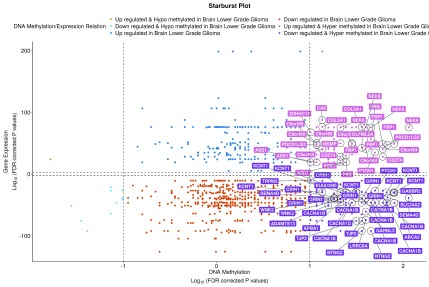
Starburst plot: x-axis is the log10 of the correct P-value for DNA methylation and the y-axis is the log10 of the correct P-value for the expression data. The starburst plot highlights nine distinct quadrants. Highlighted genes might have the potential for activation due to epigenetic alterations.

***Integration of DNA methylation & gene expression data.*** After finding differentially methylated CpG sites, one might ask whether nearby genes also undergo a change in expression either an increase or a decrease. DNA methylation at promoters of genes has been shown to be associated with silencing of the respective gene. The starburst plot is proposed to combine information from two volcano plots and is applicable for studies of DNA methylation and gene expression ^55^. Even though, being desirable that both gene expression and DNA methylation data are from the same samples, the starburst plot can be applied as a meta-analysis tool, combining data from different samples ^56^. We used the *TCGAvisualize_starburst* function to create a starburst plot. The *log*_10_ (FDR-corrected P value) for DNA methylation is plotted on the x axis, and for gene expression on the y axis, for each gene. The horizontal black dashed line shows the FDR-adjusted P value of 10 ^–2^ for the expression data and the vertical ones shows the FDR-adjusted P value of 10 ^–2^ for the DNA methylation data. The starburst plot for the Listing 23 is shown in Figure 13. While the argument met.p.cut and exp.p.cut controls the black dashed lines, the arguments *diffmean.cut* and *logFC.cut* will be used to highlight the genes that respects these parameters (circled genes in Figure 13). For the example below we set higher p.cuts trying to get the most significant list of pair gene/probes. But for the next sections we will use *exp.p.cut = 0.01* and *logFC.cut = 1* as the previous sections.


 
                        1 
                        #------------------- Starburst plot ------------------------------
 
                        2 starburst 
                        <– 
                        TCGAvisualize
                        _
                        starburst(met.chr9,    
                        # DNA methylation with results
 
                        3                                      dataDEGs,    
                        # DEG results
 
                        4                                      group1 = 
                        "Glioblastoma Multiforme"
                        ,
 
                        5                                      group2 = 
                        "Brain Lower Grade Glioma"
                        ,
 
                        6                                      filename = 
                        "starburst.png"
                        ,
 
                        7                                      met.p.
                        cut 
                        = 10^−2,
 
                        8                                          
                        exp
                        .p.
                        cut 
                        = 10^−2,
 
                        9                                      diffmean.
                        cut 
                        = 0.25,

                        10                                      logFC.
                        cut 
                        = 1,width = 15,height = 10,
11                                          
                        names 
                        = TRUE)
                    


**Listing 23. Starburst plot for comparison of DNA methylation and gene expression**

***ChIP-seq analysis.*** ChIP-seq is used primarily to determine how transcription factors and other chromatin-associated proteins influence phenotype-affecting mechanisms. Determining how proteins interact with DNA to regulate gene expression is essential for fully understanding many biological processes and disease states. The aim is to explore significant overlap datasets for inferring co-regulation or transcription factor complex for further investigation. A summary of the association of each histone mark is shown in Table 4. Besides, ChIP-seq data exists in the ROADMAP database and can be obtained through the AnnotationHub package ^57^ or from Roadmap web portal. The Table 5 shows the description for all the Roadmap files that are available through AnnotationHub. After obtaining the ChIP-seq data, we can then identify overlapping regions with the regions identified in the starburst plot. The narrowPeak files are the ones selected for this step.

**Table 4.** Histone marks that define genomic elements.

Histone marks	Role
Histone H3 lysine 4 trimethylation (H3K4me3)	Promoter regions ^58, 59^
Histone H3 lysine 4 monomethylation (H3K4me1)	Enhancer regions ^58^
Histone H3 lysine 36 trimethylation (H3K36me3)	Transcribed regions
Histone H3 lysine 27 trimethylation (H3K27me3)	Polycomb repression ^60^
Histone H3 lysine 9 trimethylation (H3K9me3)	Heterochromatin regions ^61^
Histone H3 acetylated at lysine 27 (H3K27ac)	Increase activation of genomic elements ^62– 64^
Histone H3 lysine 9 acetylation (H3K9ac)	Transcriptional activation ^65^

**Table 5.** ChIP-seq data file types available in AnnotationHub.

File	Description
fc.signal.bigwig	Bigwig File containing fold enrichment signal tracks
pval.signal.bigwig	Bigwig File containing -log10(p-value) signal tracks
hotspot.fdr0.01.broad.bed.gz	Broad domains on enrichment for DNase-seq for consolidated epigenomes
hotspot.broad.bed.gz	Broad domains on enrichment for DNase-seq for consolidated epigenomes
broadPeak.gz	Broad ChIP-seq peaks for consolidated epigenomes
gappedPeak.gz	Gapped ChIP-seq peaks for consolidated epigenomes
narrowPeak.gz	Narrow ChIP-seq peaks for consolidated epigenomes
hotspot.fdr0.01.peaks.bed.gz	Narrow DNasePeaks for consolidated epigenomes
hotspot.all.peaks.bed.gz	Narrow DNasePeaks for consolidated epigenomes
.macs2.narrowPeak.gz	Narrow DNasePeaks for consolidated epigenomes
coreMarks_mnemonics.bed.gz	15 state chromatin segmentations
mCRF_FractionalMethylation.bigwig	MeDIP/MRE(mCRF) fractional methylation calls
RRBS_FractionalMethylation.bigwig	RRBS fractional methylation calls
WGBS_FractionalMethylation.bigwig	Whole genome bisulphite fractional methylation calls

For a complete pipeline with Chip-seq data, Bioconductor provides excellent tutorials to work with ChIP-seq and we encourage our readers to review the following article ^66^.

The first step shown in Listing 24 is to download the chip-seq data. The function *query* received as argument the annotationHub database (ah) and a list of keywords to be used for searching the data, *EpigenomeRoadmap* is selecting the roadmap database, *consolidated* is selecting only the consolidate epigenomes, *brain* is selecting the brain samples, E068 is one of the epigenomes for brain (keywords can be seen in the summary table) and narrowPeak is selecting the type of file. The data downloaded is a processed data from an integrative Analysis of 111 reference human epigenomes ^67^.


 
                        1 
                        library
                        (AnnotationHub)
 
                        2 
                        library
                        (pbapply)
 
                        3 
                        #–––––––––––––––––– Working with ChipSeq data –––––––––––––––
 
                        4 
                        # Step 1: download histone marks for a brain samples.
 
                        5 
                        #––––––––––––––––––––––––––––––––––––––––––––––––––––––––––––
 
                        6 ah = AnnotationHub() 
                        # loading annotation hub database
 
                        7
 
                        8 
                        # Searching for brain consolidated epigenomes in the roadmap database
 
                        9 bpChipEpi
                        _
                        brain 
                        <– 
                        query(ah, 
                        c
                        (
                        "EpigenomeRoadMap"
                        ,
                        "narrowPeak"
                        ,
                        "chip"
                        ,
                        "consolidated"
                        ,
                        "brain"
                        ,
                        "E068"
                        ))
10
11 
                        # Get chip–seq data

                        12 histone.marks 
                        <– 
                        pblapply(
                        names
                        (bpChipEpi
                        _
                        brain), 
                        function
                        (x){ah[[x]]})
13 
                        names
                        (histone.marks) 
                        <– 
                        names
                        (bpChipEpi
                        _
                        brain)
                    


**Listing 24. Download chip-seq data**

The Chipseeker package ^68^ implements functions that uses Chip-seq data to retrieve the nearest genes around the peak, to annotate genomic region of the peak, among others. Also, it provides several visualization functions to summarize the coverage of the peak, average profile and heatmap of peaks binding to TSS regions, genomic annotation, distance to TSS and overlap of peaks or genes.

**Figure 14.**
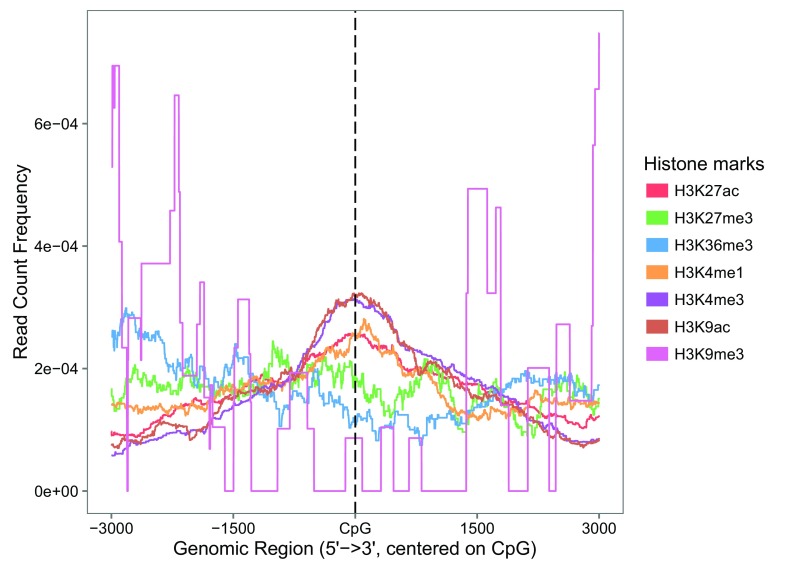
Average profiles for histone markers H3K27ac, H3K27me3, H3K36me3, H3K4me1, H3K4me3, H3K9ac, and H3K9me3. The figure indicates that the differentially methylated regions overlaps regions of enhancers, promoters and increased activation of genomic elements.

After downloading the histone marks (see Listing 24), it is useful to verify the average profile of peaks binding to hypomethylated and hypermethylated regions, which will help the user understand better the regions found. Listing 25 shows an example of code to plot the average profile. Figure 14 shows the result.

**Figure 15.**
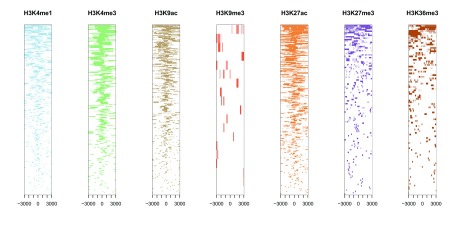
Heatmap of histone marks H3K4me1, H3K4me3, H3K27ac, H3K9ac, H3K9me3, H3K27me3 and H3K36me3 for brain tissues. The figure indicates that the most of the peaks that overlaps the probes are not brain specific.

To help the user better understand the regions found in the DMR analysis, we downloaded histone marks specific for brain tissue using the AnnotationHub package that can access the Roadmap database ( Listing 24). Next, the Chipseeker was used to visualize how histone modifications are enriched onto hypomethylated and hypermethylated regions, ( Listing 25). The enrichment heatmap and the average profile of peaks binding to those regions is shown in Figure 14 and Figure 15 respectively.

The hypomethylated and hypermethylated regions are enriched for H3K4me3, H3K9ac, H3K27ac and H3K4me1 which indicates regions of enhancers, promoters and increased activation of genomic elements. However, these regions are associated neither with transcribed regions nor Polycomb repression as the H3K36me3 and H3K27me3 heatmaps does not show an enrichment nearby the position 0, and the average profile also does not show a peak at position 0.


 
                        1 
                        library
                        (ChIPseeker)
 
                        2 
                        library
                        (pbapply)
 
                        3 
                        library
                        (SummarizedExperiment)
 
                        4 
                        library
                        (GenomeInfoDb)
 
                        5 
                        library
                        (ggplot2)
 
                        6 
                        library
                        (AnnotationHub)
 
                        7
 
                        8 
                        # Create a GR object based on the hypo
                        /
                        hypermethylated probes.
 
                        9 probes 
                        <– 
                        keepStandardChromosomes(rowRanges(met.chr9)[values(met.chr9)[,
                        "status.Glioblastoma 
        Multiforme.Brain Lower Grade Glioma"
                        ] %in% 
                        c
                        (
                        "Hypermethylated", "Hypomethylated"
                        ),])

                        10 
                        # Defining a window of 3kbp – 3kbp
                        _
                        probe
                        _
                        3kbp

                        11 probes@ranges 
                        <– 
                        IRanges(
                        start 
                        = 
                        c
                        (probes@ranges@start – 3000), 
                        end 
                        = 
                        c
                        (probes@ranges@start + 3000))
12
13 
                        ### Profile of ChIP peaks binding to TSS regions

                        14 
                        # First of all, for calculate the profile of ChIP peaks binding to TSS regions, we should

                        15 
                        # prepare the TSS regions, which are defined as the flanking sequence of the TSS sites.

                        16 
                        # Then align the peaks that are mapping to these regions, and generate the tagMatrix.

                        17 tagMatrixList 
                        <– 
                        pblapply(histone.marks, 
                        function
                        (x) {
18     getTagMatrix(keepStandardChromosomes(x), windows = probes, weightCol = 
                        "score"
                        )
19 })
20 
                        names
                        (tagMatrixList) 
                        <– basename
                        (bpChipEpi
                        _
                        brain
                        $title
                        )
21 
                        names
                        (tagMatrixList) 
                        <– gsub
                        (
                        ".narrowPeak.gz"
                        ,
                        ""
                        ,
                        names
                        (tagMatrixList)) 
                        # remove file type from name

                        22 
                        names
                        (tagMatrixList) 
                        <– gsub
                        (
                        "E068–"
                        ,
                        ""
                        ,
                        names
                        (tagMatrixList)) 
                        # remove file type from name

                        23
24 pdf(
                        "chip
                        _
                        heatmap.pdf"
                        , height = 5, width = 10)
25 tagHeatmap(tagMatrixList, xlim=
                        c
                        (–3000, 3000),color = NULL)
26 
                        dev
                        .
                        off
                        ()
27
28 p 
                        <– 
                        plotAvgProf(tagMatrixList, xlim = 
                        c
                        (–3000,3000), xlab = 
                        "Genomic Region (5'–>3', centered on CpG)"
                        )
29 
                        # We are centreing in the CpG instead of the TSS. So we’ll change the labels manually

                        30 p 
                        <– 
                        p + 
                        scale_
                        x
                        _
                        continuous(breaks=
                        c
                        (–3000,–1500,0,1500,3000),
                        labels
                        =
                        c
                        (–3000,–1500,
                        "CpG"
                        ,1500,3000))
31 
                        library
                        (ggthemes)
32 pdf(
                        "chip–seq.pdf"
                        , height = 5, width = 7)
33 p + theme
                        _
                        few() + 
                        scale_
                        colour
                        _
                        few(name=
                        "Histone marks"
                        ) +guides(colour = guide
                        _legend
                        (override.aes =
           
                        list
                        (size=4)))
34 
                        dev.off
                        ()
                    


**Listing 25. Average profile plot**

To annotate the location of a given peak in terms of genomic features, annotatePeak assigns peaks to genomic annotation in “annotation” column of the output, which includes whether a peak is in the TSS, Exon, 5’ UTR, 3’ UTR, Intronic or Intergenic location.


 
                        1 
                        require
                        (TxDb.Hsapiens.UCSC.hg19.knownGene)
 
                        2 txdb 
                        <– 
                        TxDb.Hsapiens.UCSC.hg19.knownGene
 
                        3 peakAnno 
                        <– 
                        annotatePeak(probes, tssRegion=
                        c
                        (−3000, 3000), TxDb=txdb, annoDb=
                        "org.Hs.eg.db"
                        )
 
                        4 plotAnnoPie(peakAnno)
                    


**Listing 26. Annotate the location of a given peak in terms of genomic features**

**Figure 16.**
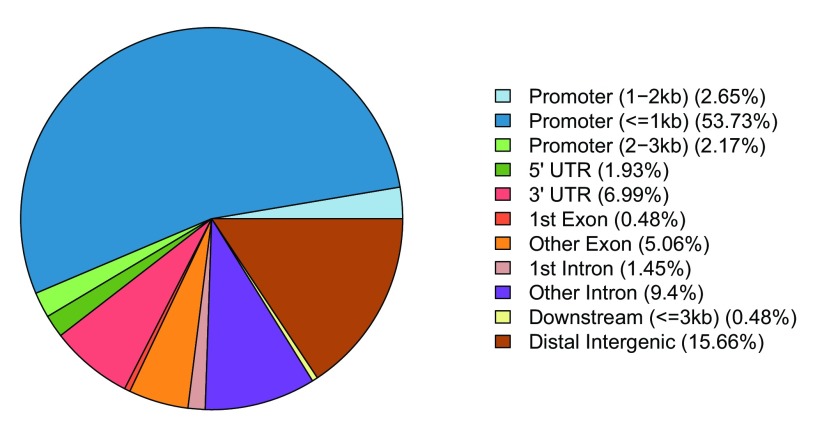
Feature distribution: annotation the region of the probes.

***Identification of Regulatory Enhancers.*** Recently, many studies suggest that enhancers play a major role as regulators of cell-specific phenotypes leading to alteration in transcriptomes realated to diseases ^69– 72^. In order to investigate regulatory enhancers that can be located at long distances upstream or downstream of target genes Bioconductor offer the Enhancer Linking by Methylation/Expression Relationship (ELMER) package. This package is designed to combine DNA methylation and gene expression data from human tissues to infer multi-level cis-regulatory networks. It uses DNA methylation to identify enhancers and correlates their state with expression of nearby genes to identify one or more transcriptional targets. Transcription factor (TF) binding site analysis of enhancers is coupled with expression analysis of all TFs to infer upstream regulators. This package can be easily applied to TCGA public available cancer data sets and custom DNA methylation and gene expression data sets ^73^.

ELMER analysis have 5 main steps:

1. Identify distal enhancer probes on HM450K.
2. Identify distal enhancer probes with significantly different DNA methylation level in control group and experiment group.
3. Identify putative target genes for differentially methylated distal enhancer probes.
4. Identify enriched motifs for the distal enhancer probes which are significantly differentially methylated and linked to putative target gene.
5. Identify regulatory TFs whose expression associate with DNA methylation at motifs.

This section shows how to use ELMER to analyze TCGA data using as example LGG and GBM samples.

***Preparing the data for ELMER package.*** After downloading the data with TCGAbiolinks package, some steps are still required to use TCGA data with ELMER. These steps can be done with the function *TCGAprepare_elmer*. This function for the DNA methylation data will remove probes with NA values in more than 20% samples and remove the annotation data, for RNA expression data it will take the log2(expression + 1) of the expression matrix in order to linearize the relation between DNA methylation and expression. Also, it will prepare the row names of the matrix as required by the package.

The Listing 27 shows how to use TCGAbiolinks ^8^ to search, download and prepare the data for the ELMER package. Due to time and memory constraints, we will use in this example only data from 10 LGG patients and 10 GBM patients that have both DNA methylation and gene expression data. This samples are the same used in the previous steps.


 
                        1 
                        #----------- 8.3  Identification of Regulatory Enhancers -------
 
                        2 
                        library
                        (TCGAbiolinks)
 
                        3 
                        library
                        (stringr)
 
                        4 
                        # Samples: primary solid tumor w
                        / 
                        DNA methylation and gene expression
 
                        5 matched
                        _
                        met
                        _exp <- function
                        (project, n = NULL){
 
                        6      
                        # get primary solid tumor samples: DNA methylation
 
                        7      
                        message
                        (
                        "Download DNA methylation information"
                        )
 
                        8      
                        met450k 
                        <- 
                        GDCquery(project = project,
 
                        9			      
                        data
                        .
                        category 
                        = 
                        "DNA methylation",

                        10			      
                        platform = 
                        "Illumina Human Methylation 450",

                        11			      
                        legacy = TRUE,

                        12			      
                        sample
                        .type = 
                        c
                        (
                        "Primary solid Tumor"
                        ))

                        13     met450k.tp 
                        <-  
                        met450k
                        $
                        results[[1]]
                        $
                        cases

                        14

                        15     
                         # get primary solid tumor samples: RNAseq

                        16      
                        message(
                        "Download gene expression information"
                        )

                        17      
                        exp <- 
                        GDCquery(project = project,

                        18			  
                        data
                        .
                        category 
                        = 
                        "Gene expression"
                        ,

                        19			  
                        data
                        .type = 
                        "Gene expression quantification"
                        ,

                        20		        platform = 
                        "Illumina HiSeq"
                        ,

                        21			  
                        file
                        .type =  
                        "results"
                        ,

                        22			  
                        sample
                        .type = 
                        c(
                        "Primary solid Tumor")
                        ,

                        23		       legacy = TRUE)

                        24      
                        exp
                        .tp 
                        <- exp
                        $
                        results[[1]]
                        $
                        cases

                        25      
                        print
                        (
                        exp
                        .tp[1:10])

                        26      
                        # Get patients with samples in both platforms

                        27      patients 
                        <- unique(
                        substr(
                        exp
                        .tp,1,15)[
                        substr(
                        exp
                        .tp,1,12) %in% 
                        substr
                        (met450k.tp,1,12)])

                        28      
                        if
                        (
                        !is
                        .
                        null
                        (n)) patients 
                        <- 
                        patients[1:n]
                         # get only n samples

                        29      
                        return
                        (patients)

                        30 }

                        31 lgg.samples 
                        <- 
                        matched
                        _
                        met
                        _exp
                        (
                        "TCGA-LGG"
                        , n = 10)

                        32 gbm.samples 
                        <- 
                        matched
                        _
                        met
                        _exp
                        (
                        "TCGA-GBM"
                        , n = 10)

                        33 samples 
                        <- c
                        (lgg.samples,gbm.samples)

                        34

                        35 
                        #-----------------------------------

                        36 
                        # 1 -  Methylation

                        37
                         #----------------------------------

                        38 query.lgg 
                        <- 
                        GDCquery(project = 
                        "TCGA-LGG"
                        ,

                        39                         
                        data.
                        category 
                        = 
                        "DNA methylation"
                        ,

                        40                       platform = 
                        "Illumina Human Methylation 450"
                        ,

                        41                       legacy = TRUE,

                        42                       barcode = lgg.samples)

                        43 GDCdownload(query.lgg)

                        44 met.lgg  
                        <-GDCprepare(query.lgg,   
                        save 
                        = FALSE)

                        45

                        46 query.gbm 
                        <- 
                        GDCquery(project = 
                        "TCGA-GBM"
                        ,

                        47                          
                        data.
                        category = 
                        "DNA methylation",

                        48                       platform = 
                        "Illumina Human Methylation 450",

                        49                       legacy = TRUE,

                        50                       barcode = gbm.samples)

                        51 GDCdownload(query.gbm)

                        52 met.gbm 
                        <- 
                        GDCprepare(query.gbm, 
                        save
                         = FALSE)

                        53 met.elmer 
                        <- 
                        SummarizedExperiment::
                        cbind
                        (met.lgg, met.gbm)

                        54 met.elmer 
                        <- TCGAprepare
                        _
                        elmer(met.elmer,
55                               platform = 
                        "HumanMethylation450"
                        )

                        56

                        57 
                        #-----------------------------------

                        58 
                        # 2 - Expression

                        59 
                        # ----------------------------------

                        60 query.
                        exp
                        .lgg 
                        <- 
                        GDCquery(project = 
                        "TCGA-LGG"
                        ,
61                   
                        data.
                        category = 
                        "Gene expression",

                        62                   
                        data
                        .type = 
                        "Gene expression quantification"
                        ,
63                 platform = 
                        "Illumina HiSeq"
                        ,
64                   
                        file
                        .type = 
                        "results"
                        ,
65                 legacy = TRUE, barcode = lgg.samples )
66 GDCdownload(query.
                        exp
                        .lgg)
67 
                        exp
                        .lgg 
                        <- 
                        GDCprepare(query.
                        exp
                        .lgg, 
                        save 
                        = FALSE)
68
69 query.
                        exp.gbm 
                        <- GDCquery(project = 
                        "TCGA-GBM"
                        ,
70                  
                        data.
                        category = 
                        "Gene expression"
                        ,
71                  
                        data.type = 
                        "Gene expression quantification",

                        72                 platform = 
                        "Illumina HiSeq"
                        ,
73                  
                        file.type = 
                        "results"
                        ,
74                legacy = TRUE, barcode = gbm.samples)
75 GDCdownload(query.
                        exp
                        .gbm)
76 
                        exp.gbm 
                        <-GDCprepare(query.
                        exp.gbm, 
                        save 
                        = FALSE)
77 
                        exp.elmer 
                        <- 
                        SummarizedExperiment::
                        cbind(
                        exp
                        .lgg, 
                        exp
                        .gbm)
78 
                        exp
                        .elmer 
                        <- 
                        TCGAprepare
                        _
                        elmer(
                        exp
                        .elmer, platform = 
                        "IlluminaHiSeq
                        _
                        RNASeqV2")


**Listing 27. Preparing TCGA data for ELMER’s mee object**

Finally, the ELMER input is a mee object that contains a DNA methylation matrix, an gene expression matrix, a probe information GRanges, the gene information GRanges and a data frame summarizing the data. It should be highlighted that samples without both the gene expression and DNA methylation data will be removed from the mee object.

By default the function *fetch.mee* that is used to create the mee object will separate the samples into two groups, the control group (normal samples) and the experiment group (tumor samples), but the user can relabel the samples to compare different groups. For the next sections, we will work with both the experimental group (LGG) and control group (GBM).


 
                        1 
                        library
                        (ELMER)
 
                        2 geneAnnot 
                        <– 
                        txs()
 
                        3 geneAnnot
                        $
                        GENEID 
                        <– 
                        paste0(
                        "ID"
                        ,geneAnnot
                        $
                        GENEID)
 
                        4 geneInfo 
                        <– 
                        promoters(geneAnnot,upstream = 0, downstream = 0)
 
                        5 probe 
                        <– get
                        .feature.probe()
 
                        6
 
                        7 
                        # create mee object, use @ to access the matrices inside the object
 
                        8 mee 
                        <– 
                        fetch.mee(meth = met.elmer, 
                        exp 
                        = 
                        exp
                        .elmer,
 
                        9 		    TCGA = TRUE, probeInfo = probe, geneInfo = geneInfo)
10
11 
                        # Relabel GBM samples in the mee object: GBM is control

                        12 mee@sample
                        $
                        TN[mee@sample
                        $
                        ID %in% lgg.samples] 
                        <– 
                        "Control"
                    


**Listing 28. Creating mee object with TCGA data to be used in ELMER**

***ELMER analysis.*** After preparing the data into a *mee* object, we executed the five ELMER steps for both the hypo (distal enhancer probes hypomethylated in the LGG group) and hyper (distal enhancer probes hypermethylated in the LGG group) direction. The code is shown below. A description of how these distal enhancer probes are identified is found in the ELMER.data vignette.


 
                        1 
                        library
                        (parallel)
 2 
                        # Available directions are hypo and hyper, we will use only hypo
 
                        3 
                        # due to speed constraint
 
                        4 direction 
                        <– c
                        (
                        "hyper"
                        )
 5
 6 
                        for
                         (j in direction){
 7     
                        print
                        (j)
 8     
                        dir
                        .out 
                        <– 
                        paste0(
                        "elmer
                        /
                        "
                        ,j)
 9     
                        dir
                        .
                        create
                        (
                        dir
                        .out, recursive = TRUE)
10     
                        #––––––––––––––––––––––––––––––––––––––

                        11     
                        # STEP 3: Analysis                     |

                        12     
                        #––––––––––––––––––––––––––––––––––––––

                        13     
                        # Step 3.1: Get diff methylated probes |

                        14     
                        #––––––––––––––––––––––––––––––––––––––

                        15    Sig.probes 
                        <– get
                        .
                        diff
                        .meth(mee, cores=detectCores(),

                        16     				      
                        dir
                        .out =
                        dir
                        .out,

                        17     				      
                        diff
                        .
                        dir
                        =j,

                        18     				  pvalue = 0.01)

                        19

                        20     
                        #–––––––––––––––––––––––––––––––––––––––––––––––––––––––––––––

                        21     
                        # Step 3.2: Identify significant probe–gene pairs            |

                        22     
                        #–––––––––––––––––––––––––––––––––––––––––––––––––––––––––––––

                        23     
                        # Collect nearby 20 genes for Sig.probes

                        24     nearGenes 
                        <– 
                        GetNearGenes(TRange=getProbeInfo(mee, probe=Sig.probes
                        $
                        probe),

                        25    				 cores=detectCores(),

                        26    				 geneAnnot=getGeneInfo(mee))

                        27

                        28     pair 
                        <– get
                        .pair(mee=mee,

                        29                      probes=
                        na
                        .
                        omit
                        (Sig.probes
                        $
                        probe),

                        30                      nearGenes=nearGenes,

                        31                      permu.
                        dir
                        =paste0(
                        dir
                        .out,
                        "
                        /
                        permu"
                        ),
32                         
                        dir
                        .out=
                        dir
                        .out,

                        33                      cores=detectCores(),

                        34                      label= j,

                        35                      permu.size=100, 
                        # For significant results use 10000

                        36                      Pe = 0.01) 
                        # For significant results use 0.001

                        37

                        38     Sig.probes.paired 
                        <– 
                        fetch.pair(pair=pair,

                        39   		    		       probeInfo = getProbeInfo(mee),

                        40      			       geneInfo = getGeneInfo(mee))

                        41     Sig.probes.paired 
                        <–read
                        .
                        csv
                        (paste0(
                        dir
                        .out,
42						
                        "
                        /
                        getPair."
                        ,j,

                        43	       
                        ".pairs.significant.csv"
                        ),

                        44     				    stringsAsFactors=FALSE)[,1]

                        45

                        46		

                        47     
                        #–––––––––––––––––––––––––––––––––––––––––––––––––––––––––––––

                        48     
                        # Step 3.3: Motif enrichment analysis on the selected probes |

                        49     
                        #–––––––––––––––––––––––––––––––––––––––––––––––––––––––––––––

                        50     
                        if
                        (
                        length
                        (Sig.probes.paired) > 0){
51          
                        #–––––––––––––––––––––––––––––––––––––––––––––––––––––––––––––

                        52          
                        # Step 3.3: Motif enrichment analysis on the selected probes |

                        53          
                        #–––––––––––––––––––––––––––––––––––––––––––––––––––––––––––––

                        54         enriched.motif 
                        <– get
                        .enriched.motif(probes=Sig.probes.paired,

                        55     						      
                        dir
                        .out=
                        dir
                        .out, label=j,	

                        56     					        background.probes = probe
                        $
                        name)

                        57         motif.enrichment 
                        <– read
                        .
                        csv
                        (paste0(
                        dir
                        .out,
58					     
                        "
                        /
                        getMotif."
                        ,j,

                        59		  
                        ".motif.enrichment.csv"
                        ),

                        60     				       stringsAsFactors=FALSE)

                        61          
                        if
                        (
                        length
                        (enriched.motif) > 0){

                        62               
                        #–––––––––––––––––––––––––––––––––––––––––––––––––––––––––––––

                        63               
                        # Step 3.4: Identifying regulatory TFs                        |

                        64               
                        #–––––––––––––––––––––––––––––––––––––––––––––––––––––––––––––

                        65               
                        print
                        (
                        "get.TFs"
                        )

                        66

                        67             TF 
                        <– get
                        .TFs(mee = mee,

                        68     			     enriched.motif = enriched.motif,

                        69     			         
                        dir
                        .out = 
                        dir
                        .out,

                        70     			     cores = detectCores(), label = j)

                        71             TF.meth.
                        cor <– get
                        (
                        load
                        (paste0(
                        dir
                        .out,

                        72					     
                        "
                        /
                        getTF."
                        ,j,

                        73		  
                        ".TFs.with.motif.pvalue.rda"
                        )))

                        74               
                        save
                        (TF, enriched.motif, Sig.probes.paired,

                        75                  pair, nearGenes, Sig.probes, 

                        76                  motif.enrichment, TF.meth.
                        cor
                        ,

                        77                    
                        file
                        =paste0(
                        dir
                        .out,
                        "
                        /
                        ELMER
                        _
                        results
                        _
                        "
                        ,j,
                        ".rda"
                        ))

                        78         }

                        79     }

                        80 }
                    


**Listing 29. Running ELMER analysis**

**Figure 17.**
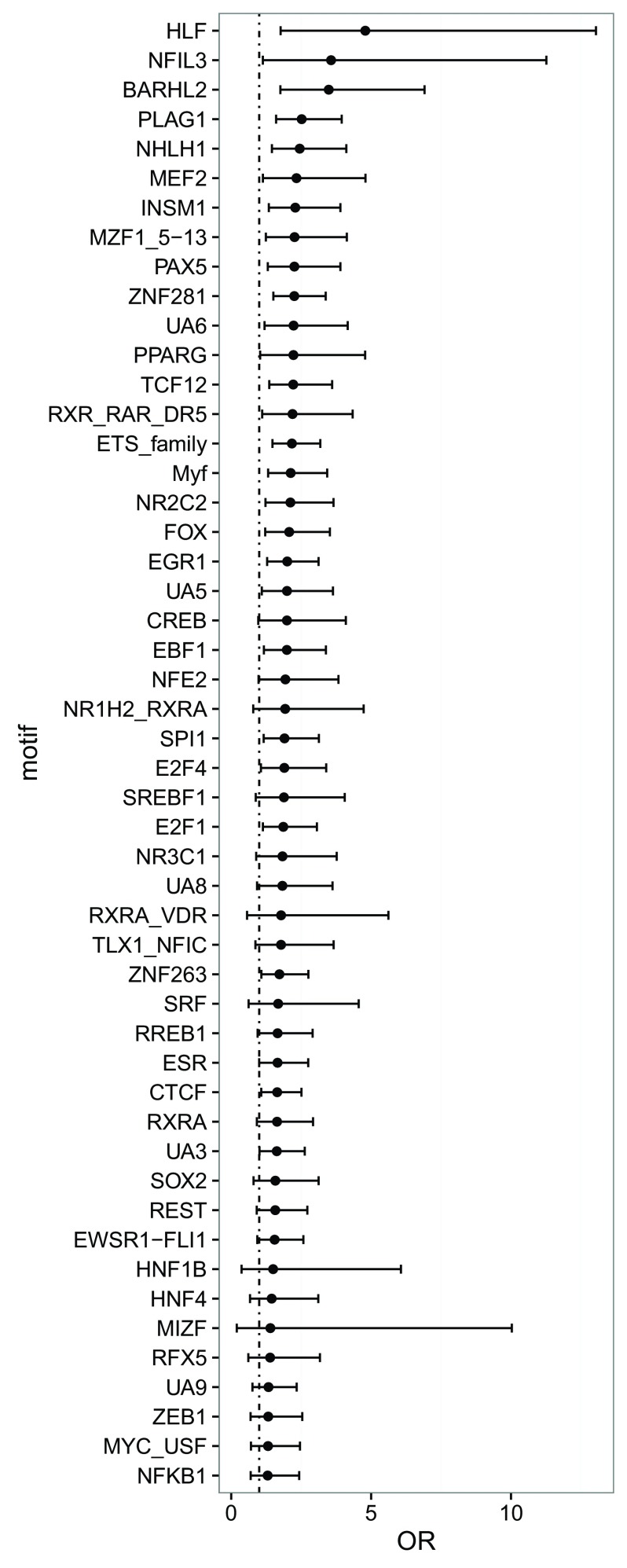
The plot shows the Odds Ratio (x axis) for the selected motifs. The range shows the 95% confidence interval for each Odds Ratio.

When ELMER Identifies the enriched motifs for the distal enhancer probes which are significantly differentially methylated and linked to putative target gene, it will plot the Odds Ratio (x axis) for the each motifs found. The list of motifs for the hyper direction (probes hypermethylated in LGG group compared to the GBM group) is found in the Figure 17. We selected motifs that had a minimum incidence of 10 in the given probes set and the smallest lower boundary of 95% confidence interval for Odds Ratio of 1.1. These both values are the default from the ELMER package.

**Figure 18.**
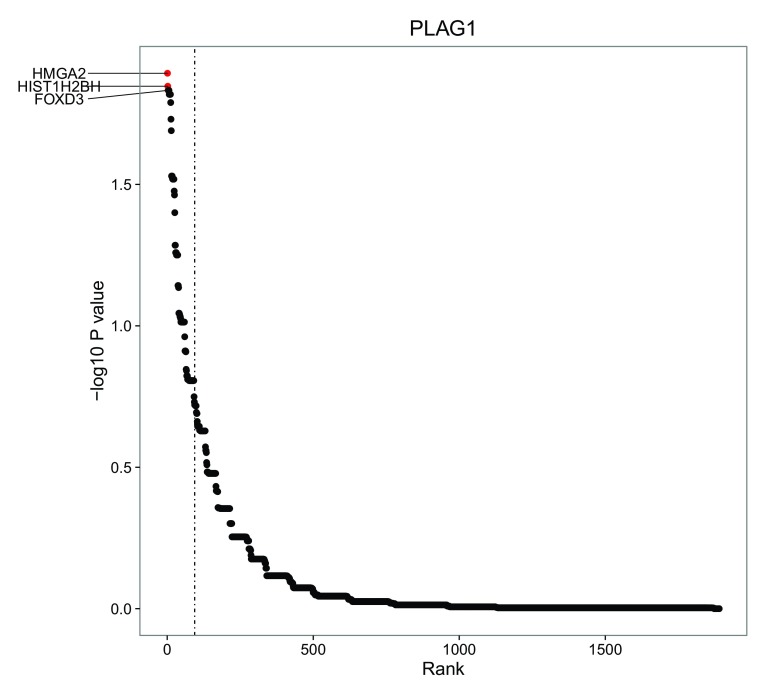
TF ranking plots based on the score ( *-log*( *Pvalue*)) of association between TF expression and DNA methylation of the FOX motif. The dashed line indicates the boundary of the top 5% association scores and the TFs within this boundary were considered candidate upstream regulators. The top three associated TFs and the TF family members (dots in red) that are associated with that specific motif are labeled in the plot.

The analysis found 16 enriched motifs for the hyper direction and no enriched motifs for the hypo direction. After finding the enriched motifs, ELMER identifies regulatory transcription factors (TFs) whose expression is associated with DNA methylation at motifs. ELMER automatically creates a TF ranking plot for each enriched motif. This plot shows the TF ranking plots based on the association score ( *−log*( *Pvalue*)) between TF expression and DNA methylation of the motif. We can see in Figure 18 that the top three TFs that are associated with that FOX motif are FOXD3, HMGA2 and HIST1H2BH.

The output of this step is a data frame with the following columns:

1. motif: the names of motif.
2. top.potential.TF: the highest ranking upstream TFs which are known recognized the motif.
3. potential.TFs: TFs which are within top 5% list and are known recognized the motif. top5percent: all TFs which are within top 5% list considered candidate upstream regulators

**Figure 19.**
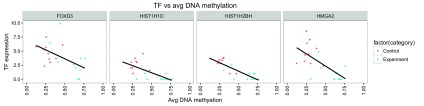
Each scatter plot shows the average DNA methylation level of sites with the FOX motif plotted against the expression of the TFs FOXD3, HIST1H1D, HIST1H2BH, HMGA2 respectively.


 
Also, for each motif we can take a look at the three most relevant TFs. For example, Figure 19 shows the average DNA methylation level of sites with the FOX motif plotted against the expression of the TFs FOXD3, HIST1H1D, HIST1H2BH and HMGA2. We can see that the experiment group (LGG samples) has a lower average methylation level of sites with the FOX motif plotted and a higher average expression of the TFs.

                        1 scatter.
                        plot
                        (mee, 
                        category
                        =
                        "TN"
                        , 
                        save
                        =T, 
                        lm_
                        line=TRUE,
 
                        2              byTF=
                        list
                        (TF=
                        c
                        (
                        "FOXD3"
                        ,
                        "HIST1H1D"
                        ,
                        "HIST1H2BH"
                        ,
                        "HMGA2"
                        ),
 
                        3              probe=enriched.motif[[
                        "FOX"
                        ]]))
                    


**Listing 30. Visualizing the average DNA methylation level of sites with a chosen motif vs TF expression**

And for each relevant TF we will use the clinical data to analyze the survival curves for the 30% patients with higher expression of that TF versus the 30% with lower expression. The code below shows that analysis.


 
                        1 TCGAsurvival
                        _
                        TFplot 
                        <– function
                        (TF,mee, clinical, percentage = 0.3){
 
                        2
 
                        3    
                        # For the transcription factor, gets it getGeneID
 
                        4    gene 
                        <– 
                        getGeneID(mee,
                        symbol
                        =TF)
 
                        5    
                        # Get the expression values for the genes.
 
                        6    
                        # (getExp is a ELMER function)
 
                        7    
                        exp <– 
                        getExp(mee,geneID=gene)
 
                        8
 
                        9    
                        # Get the names of the 30% patients with lower expression

                        10    g1 
                        <– names
                        (
                        sort
                        (
                        exp
                        )[1:
                        ceiling
                        (
                        length
                        (
                        exp
                        ) 
                        ∗ 
                        percentage)])
11
12   
                         # Get the names of the 30% patients with higher expression

                        13    g2 
                        <– names
                        (
                        sort
                        (
                        exp
                        , decreasing = T)[1:
                        ceiling
                        (
                        length
                        (
                        exp
                        ) 
                        ∗ 
                        percentage)])
14
15    
                        # get the data of only these patients

                        16    idx 
                        <– 
                        clinical
                        $
                        bcr
                        _
                        patient
                        _
                        barcode %in% 
                        substr
                        (
                        c
                        (g1, g2),1,12)

                        17    clinical 
                        <– 
                        clinical[idx,]
18    
                        # Create the labels for each sample

                        19    clinical
                        $
                        tf
                        _
                        groups 
                        <– 
                        "high"

                        20    low.idx 
                        <– 
                        clinical
                        $
                        bcr
                        _
                        patient
                        _
                        barcode %in%  
                        substr
                        (
                        c
                        (g1),1,12)

                        21    clinical[low.idx,]
                        $
                        tf
                        _
                        groups 
                        <– 
                        "low"

                        22
23    
                        # Use TCGAbiolinks to create the survival curve

                        24    TCGAanalyze
                        _
                        survival(clinical,
                        "tf
                        _
                        groups"
                        ,
25                           
                        legend
                        =paste0(TF,
                        "Exp level"
                        ),
26                         filename = paste0(TF,
                        ".pdf"
                        ))
27 }
28
29 
                        # get clinical patient data for GBM samples

                        30 gbm
                        _
                        clin 
                        <– 
                        GDCquery
                        _
                        clinic(
                        "TCGA-GBM"
                        ,
                        "Clinical"
                        )
31
32 
                        # get clinical patient data for LGG samples

                        33 lgg
                        _
                        clin 
                        <– 
                        GDCquery
                        _
                        clinic(
                        "TCGA-LGG"
                        ,
                        "Clinical"
                        )
34
35 
                        # Bind the results, as the columns might not be the same,

                        36 
                        # we will will plyr rbind.fill, to have all columns from both files

                        37 clinical 
                        <– 
                        plyr::
                        rbind
                        .fill(gbm
                        _
                        clin,lgg
                        _
                        clin)
38 
                        # Call the function we created

                        39 TCGAsurvival
                        _
                        TFplot(
                        "ELF4"
                        ,mee,clinical)
40 TCGAsurvival
                        _
                        TFplot(
                        "HMGA2"
                        ,mee,clinical)
                        
41 TCGAsurvival
                        _
                        TFplot(
                        "FOXD3"
                        ,mee,clinical)
                    


**Listing 31. Survival analysis for samples with both lower and higher expression of regulatory TFs.**

**Figure 20.**
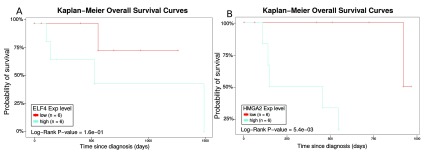
**A**) Survival plot for the 30% patients with high expression and low expression of ELF4 TF. **B**) Survival plot for the 30% patients with high expression and low expression of HGMA2 TF.

The Figure 20, shows that the samples with lower expression of some of these TFs have a better survival than those with higher expression.

## Conclusion

This workflow outlines how one can use specific Bioconductor packages for the analysis of cancer genomics and epigenomics data derived from the TCGA. In addition, we highlight the importance of using ENCODE and Roadmap data to inform on the biology of the non-coding elements defined by functional roles in gene regulation. We introduced TCGAbiolinks and RTCGAToolbox Bioconductor packages in order to illustrate how one can acquire TCGA specific data, followed by key steps for genomics analysis using GAIA package, for transcriptomic analysis using TCGAbiolinks, dnet, pathview packages and for DNA methylation analysis using TCGAbiolinks package. An inference of gene regulatory networks was also introduced by MINET package. Finally, we introduced Bioconductor packages AnnotationHub, ChIPSeeker, ComplexHeatmap, and ELMER to illustrate how one can acquire ENCODE/Roadmap data and integrate these data with the results obtained from analyzing TCGA data to identify and characterize candidate regulatory enhancers associated with cancer.

## Data and software availability

This workflow depends on various packages from version 3.2 of the Bioconductor project, running on R version 3.2.2 or higher. It requires a number of software packages, including AnnotationHub, ChIPSeeker, ELMER, ComplexHeatmap, GAIA, rGADEM, MotIV, MINET, RTCGAToolbox and TCGAbiolinks. Version numbers for all packages used are in section "Session Information". Listing 32 shows how to install all the required packages.


 
                    1 
                    source
                    (
                    "https:
                    //
                    bioconductor.org
                    /
                    biocLite.R"
                    )
 
                    2 
                    packages <– c
                    (
                    "TCGAbiolinks"
                    ,
                    "ELMER"
                    ,
                    "gaia"
                    ,
                    "ChIPseeker"
                    ,
 
                    3 		   
                    "AnnotationHub"
                    , 
                    "ComplexHeatmap"
                    ,
 
                    4 		   
                    "clusterProfiler"
                    , 
                    "RTCGAToolbox"
                    ,
 
                    5 		   
                    "minet"
                    ,
                    "biomaRt"
                    ,
                    "pathview"
                    , 
                    "MotifDb"
                    ,
 
                    6 		   
                    "MotIV"
                    ,
                    "motifStack"
                    ,
                    "rGADEM"
                    )
 
                    7 
                    new.packages <– packages
                    [
                    !
                    (
                    packages 
                    %in% 
                    installed
                    .
                    packages
                    ()
                    [,
                    "Package"
                    ])]
 
                    8 
                    if
                    (
                    length
                    (
                    new
                    .
                    packages
                    )) biocLite(
                    new
                    .
                    packages
                    )
 
                    9 
                    if
                    (
                    !require
                    (
                    "dnet"
                    )) 
                    install
                    .
                    packages
                    (
                    "dnet"
                    )
10 
                    if
                    (
                    !require
                    (
                    "circlize"
                    )) 
                    install
                    .
                    packages
                    (
                    "circlize"
                    )
11 
                    if
                    (
                    !require
                    (
                    "VennDiagram"
                    )) 
                    install
                    .
                    packages
                    (
                    "VennDiagram"
                    )
12 
                    if
                    (
                    !require
                    (
                    "c3net"
                    )) 
                    install
                    .
                    packages
                    (
                    "c3net"
                    )
13 
                    if
                    (
                    !require
                    (
                    "pbapply"
                    )) 
                    install
                    .
                    packages
                    (
                    "pbapply"
                    )
14 
                    if
                    (
                    !require
                    (
                    "gplots"
                    )) 
                    install
                    .
                    packages
                    (
                    "gplots"
                    )
                


**Listing 32. Installing packages**

All data used in this workflow is freely available and some of them can be accessed using a R/Bioconductor package. There are three main sources of data: The NCI Genomic Data Commons (GDC), a supplementary data repository with processed datasets from the Roadmap Epigenomics Project and from The Encyclopedia of DNA Elements (ENCODE) ^67^ and the BioGRID database with physical and genetic interactions information. For the first, a summary of the data available can be seen in https://gdc-portal.nci.nih.gov/ and its data can be accessed using the R/Bioconductor TCGAbiolinks package. For the second, a summary of the data available can be found in this spread sheet and the data can be accessed using the R/Bioconductor AnnotationHub package. For the third, the data should be directly download from the data portal.

## Session information


  
                    1 
                    R 
                    version 3.3.0 (2016–05–03)
  
                    2 
                    Platform
                    : x86
                    _
                    64–pc–linux–gnu (64–bit )
  
                    3 Running under: Ubuntu 16.04.1 LTS
  
                    4
  
                    5 locale:
  
                    6  [1] LC
                    _
                    CTYPE=
                    pt_
                    BR.UTF–8       LC
                    _
                    NUMERIC=
                    C
  
                    7  [3] LC
                    _
                    TIME=
                    pt_
                    BR.UTF–8        LC
                    _
                    COLLATE=en
                    _
                    US.UTF–8
  
                    8  [5] LC
                    _
                    MONETARY=
                    pt_
                    BR.UTF–8    LC
                    _
                    MESSAGES=en
                    _
                    US.UTF–8
  
                    9  [7] LC
                    _
                    PAPER=
                    pt_
                    BR.UTF–8       LC
                    _
                    NAME=
                    C
 
                    10  [9] LC
                    _
                    ADDRESS=
                    C                 
                    LC
                    _
                    TELEPHONE=
                    C
 
                    11 [11] LC
                    _
                    MEASUREMENT=
                    pt_
                    BR.UTF–8 LC
                    _
                    IDENTIFICATION=
                    C
 
                    12
 13 attached base 
                    packages
                    :
 14  [1] 
                    stats4    parallel   
                    grid      
                    stats      
                    graphics  
                    grDevices utils
 15  [8] datasets  
                    methods    
                    base
 16
 17 other attached 
                    packages
                    :
 18  
                    [1] rGADEM
                    _
                    2.20.0
 19  [2] seqLogo
                    _
                    1.38.0
 20  [3] BSgenome
                    _
                    1.40.1
 21  [4] rtracklayer
                    _
                    1.32.1
 22  [5] motifStack
                    _
                    1.16.2
 23  [6] ade4
                    _
                    1.7–4
 24  [7] grImport
                    _
                    0.9–0
 25  [8] XML
                    _
                    3.98–1.4
 26  [9] MotIV
                    _
                    1.28.0
 27 [10] pathview
                    _
                    1.12.0
 28 [11] biomaRt
                    _
                    2.28.0
 29 [12] minet
                    _
                    3.30.0
 30 [13] clusterProfiler
                    _
                    3.0.4
 31 [14] DOSE
                    _
                    2.10.7
 32 [15] AnnotationHub
                    _
                    2.4.2
 33 [16] ChIPseeker
                    _
                    1.8.7
 34 [17] ELMER
                    _
                    1.4.2
 35 [18] ELMER.
                    data_
                    1.2.2
 36 [19] Homo.sapiens
                    _
                    1.3.1
 37 [20] TxDb.Hsapiens.UCSC.hg19.knownGene
                    _
                    3.2.2
 38 [21] org.Hs.eg.db
                    _
                    3.3.0
 39 [22] GO.db
                    _
                    3.3.0
 40 [23] OrganismDbi
                    _
                    1.14.1
 41 [24] GenomicFeatures
                    _
                    1.24.5
 42 [25] AnnotationDbi
                    _
                    1.34.4
 43 [26] IlluminaHumanMethylation450kanno.ilmn12.hg19
                    _
                    0.2.1
 44 [27] minfi
                    _
                    1.18.2
 45 [28] bumphunter
                    _
                    1.12.0
 46 [29] locfit
                    _
                    1.5–9.1
 47 [30] iterators
                    _
                    1.0.8
 48 [31] foreach
                    _
                    1.4.3
 49 [32] Biostrings
                    _
                    2.40.2
 50 [33] XVector
                    _
                    0.12.1
 51 [34] SummarizedExperiment
                    _
                    1.2.3
 52 [35] GenomicRanges
                    _
                    1.24.2
 53 [36] GenomeInfoDb
                    _
                    1.8.3
 54 [37] IRanges
                    _
                    2.6.1
 55 [38] S4Vectors
                    _
                    0.10.2
 56 [39] lattice
                    _
                    0.20–33
 57 [40] Biobase
                    _
                    2.32.0
 58 [41] BiocGenerics
                    _
                    0.18.0
 59 [42] ComplexHeatmap
                    _
                    1.10.2
 60 [43] dnet
                    _
                    1.0.9
 61 [44] supraHex
                    _
                    1.10.0
 62 [45] hexbin
                    _
                    1.27.1
 63 [46] igraph
                    _
                    1.0.1
 64 [47] circlize
                    _
                    0.3.7
 65 [48] pbapply
                    _
                    1.2–1
 66 [49] gplots
                    _
                    3.0.1
 67 [50] gaia
                    _
                    2.16.0
 68 [51] readr
                    _
                    0.2.2
 69 [52] downloader
                    _
                    0.4
 70 [53] TCGAbiolinks
                    _
                    2.1.0
 
                    71
 72 loaded via a namespace (and not attached):
 73   [1] SparseM
                    _
                    1.7			ggthemes
                    _
                    3.2.0
 74   [3] prabclus
                    _
                    2.2–6		GGally
                    _
                    1.2.0
 75   [5] 
                    R
                    .methodsS3
                    _
                    1.7.1		pkgmaker
                    _
                    0.22
 76   [7] tidyr
                    _
                    0.5.1			ggplot2
                    _
                    2.1.0
 77   [9] knitr
                    _
                    1.13			aroma.light
                    _
                    3.2.0
 78  [11] multcomp
                    _
                    1.4–6 		     
                    R
                    .utils
                    _
                    2.3.0
 79  [13] 
                    data
                    .
                    table_
                    1.9.6		hwriter
                    _
                    1.3.2
 80  [15] KEGGREST
                    _
                    1.12.2		RCurl
                    _
                    1.95–4.8
 81  [17] GEOquery
                    _
                    2.38.4		doParallel
                    _
                    1.0.10
 82  [19] preprocessCore
                    _
                    1.34.0		cowplot
                    _
                    0.6.2
 83  [21] TH.
                    data_
                    1.0–7			RSQLite
                    _
                    1.0.0
 84  [23] chron
                    _
                    2.3–47			xml2
                    _
                    1.0.0
 85  [25] httpuv
                    _
                    1.3.3			assertthat
                    _
                    0.1
 86  [27] BiocInstaller
                    _
                    1.22.3		DEoptimR
                    _
                    1.0–6
 87  [29] caTools
                    _
                    1.17.1		dendextend
                    _
                    1.2.0
 88  [31] Rgraphviz
                    _
                    2.16.0		DBI
                    _
                    0.4–1
 89  [33] geneplotter
                    _
                    1.50.0		reshape
                    _
                    0.8.5
 90  [35] stringdist
                    _
                    0.9.4.1		EDASeq
                    _
                    2.6.2
 91  [37] matlab
                    _
                    1.0.2			dplyr
                    _
                    0.5.0
 92  [39] trimcluster
                    _
                    0.1–2		annotate
                    _
                    1.50.0
 93  [41] gridBase
                    _
                    0.4–7		robustbase
                    _
                    0.92–6
 94  [43] GenomicAlignments
                    _
                    1.8.4	mclust
                    _
                    5.2
 95  [45] mnormt
                    _
                    1.5–4			cluster
                    _
                    2.0.4
 96  [47] ape
                    _
                    3.5			genefilter
                    _
                    1.54.2
 97  [49] edgeR
                    _
                    3.14.0			nlme
                    _
                    3.1–128
 98  [51] nnet
                    _
                    7.3–12			diptest
                    _
                    0.75–7
 99  [53] sandwich
                    _
                    2.3–4		registry
                    _
                    0.3
100  [55] affyio
                    _
                    1.42.0			matrixStats
                    _
                    0.50.2
101  [57] graph
                    _
                    1.50.0			rngtools
                    _
                    1.2.4
102  [59] base64
                    _
                    2.0			Matrix
                    _
                    1.2–6
103  [61] boot
                    _
                    1.3–18			zoo
                    _
                    1.7–13
104  [63] whisker
                    _
                    0.3–2			GlobalOptions
                    _
                    0.0.10
105  [65] png
                    _
                    0.1–7			rjson
                    _
                    0.2.15
106  [67] bitops
                    _
                    1.0–6 		     
                    R
                    .oo
                    _
                    1.20.0
107  [69] ConsensusClusterPlus
                    _
                    1.36.0 	KernSmooth
                    _
                    2.23–15
108  [71] doRNG
                    _
                    1.6 			shape
                    _
                    1.4.2
109  [73] stringr
                    _
                    1.0.0			qvalue
                    _
                    2.4.2
110  [75] nor1mix
                    _
                    1.2–1			coin
                    _
                    1.1–2
111  [77] ShortRead
                    _
                    1.30.0		scales
                    _
                    0.4.0
112  [79] GSEABase
                    _
                    1.34.0		magrittr
                    _
                    1.5
113  [81] plyr
                    _
                    1.8.4			gdata
                    _
                    2.17.0
114  [83] zlibbioc
                    _
                    1.18.0		RColorBrewer
                    _
                    1.1–2
115  [85] illuminaio
                    _
                    0.14.0		plotrix
                    _
                    3.6–3
116  [87] KEGGgraph
                    _
                    1.30.0		lme4
                    _
                    1.1–12
117  [89] Rsamtools
                    _
                    1.24.0		affy
                    _
                    1.50.0
118  [91] MASS
                    _
                    7.3–45			stringi
                    _
                    1.1.1
119  [93] GOSemSim
                    _
                    1.30.3		latticeExtra
                    _
                    0.6–28
120  [95] ggrepel
                    _
                    0.5 			tools
                    _
                    3.3.0
121  [97] gridExtra
                    _
                    2.2.1		sjPlot
                    _
                    2.0.1
122  [99] digest
                    _
                    0.6.9			shiny
                    _
                    0.13.2
123 [101] quadprog
                    _
                    1.5–5		fpc
                    _
                    2.1–10
124 [103] Rcpp
                    _
                    0.12.6			siggenes
                    _
                    1.46.0
125 [105] httr
                    _
                    1.2.1			psych
                    _
                    1.6.6
126 [107] kernlab
                    _
                    0.9–24 		     
                    effects_
                    3.1–1
127 [109] sjstats
                    _
                    0.2.0			colorspace
                    _
                    1.2–6
128 [111] rvest
                    _
                    0.3.2			topGO
                    _
                    2.24.0
129 [113] splines
                    _
                    3.3.0			RBGL
                    _
                    1.48.1
130 [115] multtest
                    _
                    2.28.0		flexmix
                    _
                    2.3–13
131 [117] xtable
                    _
                    1.8–2			jsonlite
                    _
                    1.0
132 [119] nloptr
                    _
                    1.0.4			UpSetR
                    _
                    1.2.2
133 [121] modeltools
                    _
                    0.2–21		R6
                    _
                    2.1.2
134 [123] htmltools
                    _
                    0.3.5		mime
                    _
                    0.5
135 [125] minqa
                    _
                    1.2.4			BiocParallel
                    _
                    1.6.3
136 [127] DESeq
                    _
                    1.24.0 		     
                    class_
                    7.3–14
137 [129] interactiveDisplayBase
                    _
                    1.10.3	beanplot
                    _
                    1.2
138 [131] codetools
                    _
                    0.2–14		mvtnorm
                    _
                    1.0–5
139 [133] tibble
                    _
                    1.1			gtools
                    _
                    3.5.0
140 [135] openssl
                    _
                    0.9.4			survival
                    _
                    2.39–5
141 [137] limma
                    _
                    3.28.17			munsell
                    _
                    0.4.3
142 [139] DO.db
                    _
                    2.9			GetoptLong
                    _
                    0.1.3
143 [141] sjmisc
                    _
                    1.8			haven
                    _
                    0.2.1
144 [143] reshape2
                    _
                    1.4.1		gtable
                    _
                    0.2.0


