# Supplementary material for: TCGA Workflow: Analyze cancer genomics and epigenomics data using Bioconductor packages
Source: F1000Res. 2016 Dec 28;5:1542. Originally published 2016 Jun 29. [Version 2] doi: 10.12688/f1000research.8923.2 (PMC5302158; doi:10.12688/f1000research.8923.2)
Supplement: Peer review history

# Peer review: Referee response for version 2

**DOI:** 10.5256/f1000research.11063.r19656

**Published:** 2017-02-06

**Authors:** Charlotte Soneson

**Affiliations:** 1 Institute of Molecular Life Sciences, University of Zurich (UZH), Zürich, Switzerland

This article provides a comprehensive overview of how to query, download and analyze data from large cancer consortia using R and Bioconductor. Overall, I find it a useful contribution to the field, and it illustrates many of the use cases that will likely be of interest to other researchers. However, as it currently is, I find that it suffers from a few problems that I think that the authors could relatively easily fix.

 Most importantly, it should be asserted that all the code actually runs. For example, the code in listings 2 and 4 will not run since the functions are capitalized in the wrong way, and the code in listing 7 will not work since the "lastDate" variable is not defined. Also, the session info does not seem to correspond to the same session as was used in the workflow since, for example, the RTCGAToolbox package is not listed as being loaded. 

 Also, as the code is currently provided (in listings, with row numbers), I could not find a way to directly cut and paste it into an R session. Given the amount of code included in this workflow, it would be good if that could be resolved. 

 Since the workflow contains a lot of code, it would be useful to add a bit more comments, explaining what each code chunk, and each part of a chunk, is doing. Moreover, the different code chunks have different formatting styles, and it would be easier to read if this was harmonized. For example, comments are formatted differently, spaces are used inconsistently, and even within a listing (e.g., listing 2) the function calls use different formatting (with or without named arguments). Finally, not all code chunks are in "listings", and it is not clear to me why. 

 Throughout the text, the authors should also make sure to be consistent in terms of capitalization. In several places there are differences in capitalization between the text and the code (e.g., GDCquery_Maf()), or between different places in the text. There are also discrepancies in whether function names are italicized or not. The function GDCquery_clinic is referred to as GDCquery_clinical in the text. Within one section, the AAIC is spelled out as "Array Array", "Array-array" and "array-array".

 Just after listing 2, it is stated that you can use "rowRanges to gets the range of values in each row" of the SummarizedExperiment object. This should be clarified, since it returns a GRanges object describing the features in the rows. 

 The "p-value corrected FDR" should also be replaced by e.g. "corrected (or adjusted) p-value" or "FDR". 

 Bioconductor should be capitalized. 

 The "broadinstitute website" should be changed to "Broad Institute website" and include a link.

 The "nce" is not defined as far as I could see, and the sentence just before the "Retrieving known interactions" seems unfinished. It is not clear to me how the information that "There are 3,941 unique interactions between the 2,901 differentially expressed genes" was obtained, since the function in the code chunk above is not run until later. 

 It would be easier to get an overview of the text of the headers were more consistent. For example, the gene regulatory network section has subheaders that are larger than the main section header. 

 Finally, as mentioned by one of the other reviewers, the paper would benefit from modifications to the text in order to make it more readable. There are many grammatical errors, long and complicated sentences, and sentences that are unfinished and thus difficult to understand. 

 I believe that if the authors can make sure that all the code in the workflow runs as expected, and the text and code is clarified and harmonized throughout the manuscript, this can be a very valuable addition to the literature.

I have read this submission. I believe that I have an appropriate level of expertise to confirm that it is of an acceptable scientific standard, however I have significant reservations, as outlined above.

# Peer review: Referee response for version 1

**DOI:** 10.5256/f1000research.9601.r15858

**Published:** 2016-09-21

**Authors:** Elena Papaleo

**Affiliations:** 1 Computational Biology Laboratory, Unit of Statistics, Bioinformatics and Registry, Danish Cancer Society Research Center, Copenhagen, Denmark

As it has been pointed out already by the first reviewer, it is important to verify that the pipeline is updated according to the data migration to the GCD server.

 Apart from this, I find this manuscript very nice and the workflow an important contribution in the field, allowing to make accessible large datasets with genomics profiling of cancer patients to the community and even for not advanced R-users. I would like to compliment with the authors to make such a comprehensive workflow available.

 Some minor points before final publication is indexed:

- The main issue with this manuscript is that it will need an extensive revision by a native English speaker since some sentences are hard to read and very often they risk to convey the wrong message or to be misinterpreted. One example from the abstract, "we provide a series of biologically focused integrative downstream analyses of different molecular data" includes to many adjectives. Few lines below "we provide a workplan to identify candidate biologically relevant functional epigenomic elements associated with cancer", this sentence does not sound that right in English.
- Be also careful in the style use to call the software or Bioconductor packages in the text since sometimes they are italics and other times they are not.
- There is also a lot of redundancy on some of the statements in the introduction that if removed will improve the readability. For example the sentence at the end of the first paragraph of the introduction starting with " Here we describe a comprehensive workflow that integrates many bioconductor packages..." is probably not needed since the same concept is provided at the end of the introduction in the same page.
- In the Introduction when TCGA data are illustrated I would also mention metastatic samples (they are there, isn't it? or may be I don't recall correctly...)
- I suggest to change the title of the first section of the methods from 'Experimental data' to 'Access to the data' or something similar so that it will better reflect the content of the paragraph.
- I felt that sometimes the manuscript sounds more a user guide than an article, so I would suggest taking as an example the section on methylation to improve the discussion of the analyses outcome and aims in the other paragraphs.
- Also please be careful to make things accessible to any readers for example at page 4, the authors introduce the GISTIC data without explaining them in details.
- The sentence at page 4 "the data used in this workflow are published data and freely available" might be redundant so the authors could remove it.
- Page 4 summarizedExperiment object is missing the reference to the original paper about summarizedExperiment format.
- Page 5: in the text the authors illustrate assay, rowRanges and colData but in the example the order is different, i.e. assay, colData, rowRanges - I would suggest to keep consistency in the order between main text and examples (it also holds for other places in the paper).
- I am not sure I can understand the sentence at page 5, "the users should use all the follow up files in your analyses, not just the latest version".
- Page 6: the sentence beginning with "Finally, the Cancer Genome Atlas (TCGA) Research Network has reported integrated genome-wide studies of various diseases, in what is called 'Pan Cancer'..." I am not sure fits within the paragraph where the main focus is on subtypes. It sounds a bit confusing for the reader.
- Not sure that Listing 5 also fits there... why not mention it before together with the others summ.exp. accessors.
- In writing it is important that the authors really pay attention to convey the message that it is an integrative approach ... at the very first reading of the manuscript I felt that it was more a list of tools. and the integrative part of it was missing
- Page 18: a reference is missing for the AAIC method and an explanation to the reader about the choice of the 0.6 correlation cutoff.
- In the list at page 18 with the normalization, I believe that there are some repetitions (i.e. global scaling and full-quantile are appearing twice, also there are some typos).
- Page 19, I might have missed something but I cannot find in maintext any explanation of the DEA pipeline and more comments would be needed there for a new user to really appreciate it and its importance.
- Page 20 on the second line I believe a ). is missing.
- On PEA, I was wondering why for example the R package for Reactome was not integrated here and if the authors could comment on this.
- Page 23, why for protein-protein interactions only BioGrid is used and not for example a more comprehensive resource such as I2D (Interologous Interaction Database).

I have read this submission. I believe that I have an appropriate level of expertise to confirm that it is of an acceptable scientific standard.

# Response

**Published:** 2016-12-28

**Authors:** Tiago Chedraoui Silva

**Affiliations:** University of São Paulo, Brazil

Dear Elena Papaleo,  

 First, we would like to thank you for your review and for providing a detailed feedback for our workflow. We have made several changes in this new version (2) of the workflow. The changes and responses of all authors to these points are described below:

- "As it has been pointed out already by the first reviewer, it is important to verify that the pipeline is updated according to the data migration to the GCD server."
  - TCGAbiolinks package was entirely redesigned to query, download and prepare data from the GDC NCI data portal ( https://gdc.nci.nih.gov/). All the codes are working and we will submit very soon the workflow to Bioconductor. For the moment, the RMarkdown can be found in our Github repository ( https://github.com/BioinformaticsFMRP/f1000_TCGA_Workflow/blob/master/f1000.Rmd).
- "Be also careful in the style use to call the software or Bioconductor packages in the text since sometimes they are italics and other times they are not."
  - We applied the following pattern: name of the packages normal with a link to the package, name of functions and objects in italics.
- "I felt that sometimes the manuscript sounds more a user guide than an article, so I would suggest taking as an example the section on methylation to improve the discussion of the analyses outcome and aims in the other paragraphs."
  - Our main focus was on using the tools (in a reasonable time) rather than analyzing the results. The analysis itself can be verified with some articles of our group, for example, the DNA methylation analysis was described in Ceccarelli, Michele, *et al.* " Molecular profiling reveals biologically discrete subsets and pathways of progression in diffuse glioma." Cell 164.3 (2016): 550-563 as well as the analysis performed by the ELMER tool which is described in Yao, Lijing, *et al.* " Inferring regulatory element landscapes and transcription factor networks from cancer methylomes." Genome biology 16.1 (2015): 1.
- "In writing it is important that the authors really pay attention to convey the message that it is an integrative approach ... at the very first reading of the manuscript I felt that it was more a list of tools. and the integrative part of it was missing"
  - We agree with you. The article starts with specific analysis, which might cover 65% of the content and unfortunately it might lead to the feeling of the missing integrative approach. Also, we decided not to go much deeper into the analyzes because they already exist in the referenced articles and we focus more on the execution of the analyzes with the tools of the bioconductor project, something that was not covered by the cited articles, which might also give the feeling of  a "list of tools". But, the integrative analysis is shown strongly in the last two sections. We may highlight that ELMER, for example, uses DNA methylation, gene expression, histone marks, motif information and clinical data for its analysis. Also, after the DNA methylation analysis available in TCGAbiolinks package, we do a motif analysis on those regions and integrated the roadmap histone marks data to evaluate them. Finally, another integrative analysis presented in the TCGAbiolinks package is the starburst plot which integrates the differential expression analysis results with the DNA methylation results and we try to identify if the nearest gene might have been affected by the change of the DNA methylation.
- "Page 18: a reference is missing for the AAIC method and an explanation to the reader about the choice of the 0.6 correlation cutoff."
  - Array-array intensity correlation plot (AAIC) is a re-adaptation of a affyQCReport::correlationPlot working with data from gene expression of class RangedSummarizedExperiment as output of GDCprepare.Reference from https://www.bioconductor.org/packages/devel/bioc/vignettes/affyQCReport/inst/doc/affyQCReport.pdf. AAIC shows an heat map of the array-array Spearman / Pearson rank correlation coefficients. The arrays are ordered using the phenotypic data (if available) in order to place arrays with similar samples adjacent to each other. Self-self correlations are on the diagonal and by definition have a correlation coefficient of 1.0. Data from similar tissues or treatments will tend to have higher coefficients. This plot is useful for detecting outliers, failed hybridizations, or mistracked samples.
  - The 0.6 threshold came out from unsupervised hierarchical clustering with ward methodology that obtained distinct groups of samples and first one had pearson correlation lower than 0.6 considering them as possible outliers.
- "Page 19, I might have missed something but I cannot find in maintext any explanation of the DEA pipeline and more comments would be needed there for a new user to really appreciate it and its importance."
  - For DEA pipeline we used the TCGAanalyze_DEA function from our package TCGAbiolinks, that allows user to perform Differentially expression analysis (DEA), using edgeR package to identify differentially expressed genes (DEGs). It is possible to do a two-class analysis.
  - TCGAanalyze_DEA performs DEA using following functions from edgeR:
    - edgeR::DGEList converts the count matrix into an edgeR object.
    - edgeR::estimateCommonDisp each gene gets assigned the same dispersion estimate.
    - edgeR::exactTest performs pair-wise tests for differential expression between two groups.
    - edgeR::topTags takes the output from exactTest(), adjusts the raw p-values using the False Discovery Rate (FDR) correction, and returns the top differentially expressed genes.
- "On PEA, I was wondering why for example the R package for Reactome was not integrated here and if the authors could comment on this."
  - Thank you for this suggestion we are working to integrate Reactome as source of genes annotated within pathways.
  - Our PEA provided only one plot to show Gene ontologies and pathways enriched by a list of genes to have an overview of top biological functions and pathways altered by molecules inside the gene signature. We focused mainly on Bioconductor packages, but in particular we are interested to add wrapping to some functionalities of the ReactomePA package.
- "Page 23, why for protein-protein interactions only BioGrid is used and not for example a more comprehensive resource such as I2D (Interologous Interaction Database)."
  - Different sources for protein-protein interactions are available. We used BioGrid as an example, but the users can chose their preferred database such as I2D

Regarding the other points:

- We revised the text to correct links, references and remove redundancies.
- We added metastatic samples as one of the available sample types
- We changed the name of the section 'Experimental data' to 'Access to the data'
- We included a paragraph introducing GISTIC data

# Peer review: Referee response for version 1

**DOI:** 10.5256/f1000research.9601.r14695

**Published:** 2016-07-18

**Authors:** Kyle Ellrott

**Affiliations:** 1 Oregon Health & Science University, Portland, OR, USA

This review comes at a very inopportune moment. The entire software pipeline is based on the TCGAbiolinks tool kit, which downloads files from the TCGA DCC service. Unfortunately, just as this paper was being sent for review, the NCI began their migration to the GDC service. This means that all of the data services at DCC TCGA data portal (https://tcga-data.nci.nih.gov) are no longer active, and users are being directed to the GDC NCI data portal (https://gdc.nci.nih.gov/). This means that the TCGAbiolinks tool kit is broken. My attempts to run some of the examples listed in the paper where stopped by this issue. Many parts of the TCGAbiolinks will have to be fixed and/or re-written to deal with this change. This isn’t the fault of the authors, but it does render the example and pipelines described in the paper inoperable.

 There are a few technical issues that need to be addressed. The package ‘RTCGAtoolbox’ is mentioned, but actually its name is ‘RTCGAToolbox’. (lower-case T to upper-case), and because the bioconductor website is case-sensitive the provided URL ( http://bioconductor.org/packages/RTCGAtoolbox/ ) doesn’t work. On page 3, ‘TCGA Wikipedia’ should be ‘TCGA Wiki’. In the methods section on page 3, where the various data levels of TCGA are explained, on the most important aspects about the different levels is of access requirements. Data levels 1 and 2 cannot be accessed without dbGap, while level 3 and 4 are generally available for public access. 

 There is also the question of whether or not this paper fits the criteria of the Software Tools Article guidelines. The authors have presented a loose set of examples that utilize various existing, and previously published, tools. In the guidelines of F1000Research's Software Tool Articles, the criteria for a paper is “We welcome the description of new software tools. A Software Tool Article should include the rationale for the development of the tool and details of the code used for its construction.”  The authors refer to the code included in the paper as a workflow, but reads more like a series of point example that the reader can copy and alter for their own research. In introducing a new piece of software, one expects that the authors of the article are responsible for the software being presented. And while this article mentioned an extensive number of R packages, I believe only one of them, TCGAbiolinks, was written by the authors, and was published last year. While the analysis they present is very detailed and covers an expansive number of topics, I’m not sure if this should be classified as ‘Software Tool Article’. On my first read through, my assumption was that the authors were responsible for all of the tools mentioned in the abstract. And this wasn’t clarified in the text. For example, in the conclusion section on page 43, they include the phrase ‘We introduced TCGAbiolinks and RTCGAtoolbox bioconductor packages in order to illustrate how one can acquire TCGA specific data’, it wasn’t until later, that I realized that the author of the RTCGAToolbox wasn’t in the author list of the paper. 

 Of course, this is at the discretion of the editors. I would feel that this material would better be presented as a review article covering the different methods of TCGA data analysis, with better notation and attributions about the source of different pieces of software.

I have read this submission. I believe that I have an appropriate level of expertise to confirm that it is of an acceptable scientific standard, however I have significant reservations, as outlined above.

# Response

**Published:** 2016-12-28

**Authors:** Tiago Chedraoui Silva

**Affiliations:** University of São Paulo, Brazil

Dear Kyle Ellrott,

 Thank you for your comments and suggestions. We made several changes in the version 2 of the workflow, some of the changes and answers to your points are below:

- TCGAbiolinks package was entirely redesigned to query, download and prepare data from the GDC NCI data portal ( https://gdc.nci.nih.gov/). All the codes are working and we will submit very soon the workflow to Bioconductor. For the moment, the RMarkdown can be found in our Github repository ( https://github.com/BioinformaticsFMRP/f1000_TCGA_Workflow/blob/master/f1000.Rmd).
- We also highlighted the difference between open (TCGA level 3 and 4 data) and controlled data (TCGA level 1 and 2 data) and we added some useful sources that can help the user request access to the controlled data.
- Despite the description of a software article, we decided by this type of article simply because there were already other workflows with that choice and we did not find any other possibility that best described this type of article. In addition, our main focus was on using the tools (in a reasonable time) rather than analyzing the results. The analysis itself can be verified with some articles of our group, for example, the DNA methylation analysis was described in **Ceccarelli, Michele, *et al*. " Molecular profiling reveals biologically discrete subsets and pathways of progression in diffuse glioma." Cell 164.3 (2016): 550-563** as well as the analysis performed by the ELMER tool which is described in **Yao, Lijing, *et al.* " Inferring regulatory element landscapes and transcription factor networks from cancer methylomes." Genome biology 16.1 (2015): 1.**
- We added to "Author contributions" that we are the authors from the R/Bioconductor packages TCGAbiolinks and GAIA. Also, I'm working on a new version of the ELMER package.
- We corrected links and references such as**** RTCGAToolbox.
